# Limited Evidence for Parallel Molecular Adaptations Associated with the Subterranean Niche in Mammals: A Comparative Study of Three Superorders

**DOI:** 10.1093/molbev/msy161

**Published:** 2018-08-20

**Authors:** Kalina T J Davies, Nigel C Bennett, Chris G Faulkes, Stephen J Rossiter

**Affiliations:** 1 School of Biological & Chemical Sciences, Queen Mary University of London, London, United Kingdom; 2 Department of Zoology & Entomology, Mammal Research Institute, University of Pretoria, Pretoria, South Africa

**Keywords:** convergent evolution, subterranean, mammals, selection, adaptation

## Abstract

Among mammals, several lineages have independently adapted to a subterranean niche and possess similar phenotypic traits for burrowing (e.g., cylindrical bodies, short limbs, and absent pinnae). Previous research on mole-rats has revealed molecular adaptations for coping with reduced oxygen, elevated carbon dioxide, and the absence of light. In contrast, almost nothing is known regarding molecular adaptations in other subterranean lineages (e.g., true moles and golden moles). Therefore, the extent to which the recurrent phenotypic adaptations of divergent subterranean taxa have arisen via parallel routes of molecular evolution remains untested. To address these issues, we analyzed ∼8,000 loci in 15 representative subterranean taxa of four independent transitions to an underground niche for signatures of positive selection and convergent amino acid substitutions. Complementary analyses were performed in nonsubterranean “control” taxa to assess the biological significance of results. We found comparable numbers of positively selected genes in each of the four subterranean groups; however, correspondence in terms of gene identity between gene sets was low. Furthermore, we did not detect evidence of more convergent amino acids among subterranean species pairs compared with levels found between nonsubterranean controls. Comparisons with nonsubterranean taxa also revealed loci either under positive selection or with convergent substitutions, with similar functional enrichment (e.g., cell adhesion, immune response, and coagulation). Given the limited indication that positive selection and convergence occurred in the same loci, we conclude that selection may have acted on different loci across subterranean mammal lineages to produce similar phenotypes.

## Introduction

The independent colonization of the same niche (e.g., marine, high-altitude, desert regions) by divergent mammalian lineages has led to pervasive and visible recurrent evolution of similar phenotypic traits (Uhen 2007; Schipper et al. 2008; Kelley and Motani 2015). By comparison, the answer to the question of whether similar traits have been shaped by selection acting on the same set of genes and regulatory pathways has, until recently, remained unobtainable. High-throughput sequencing provides the means to perform comparative genome-wide screens of positive selection across taxa (Qiu et al. 2012; Hendrickson 2013; Tsagkogeorga et al. 2015). This expanding field of comparative genomics has revealed cases of parallel molecular adaptions associated with ecological specialization. For example, high-altitude species living at low oxygen levels have been reported to show parallel molecular adaptations in hypoxia-resistance genes (Qiu et al. 2012; Hendrickson 2013; Foll et al. 2014). Similarly, recent studies have described parallel amino acid substitutions and positive selection in unrelated groups of marine mammals, which have evolved superficially similar body plans and also face comparable demands for oxygen storage (Foote et al. 2015; Zhou et al. 2015). However, despite the presence of shared substitutions in many loci, both of these studies concluded that there was limited evidence for widespread adaptive convergence related definitively to the aquatic niche.

Among mammals, several lineages have independently undergone ecological shifts from above ground to a subterranean niche. While living below ground offers protection from predators and extreme heat, it also poses particular challenges, including reduced oxygen levels (hypoxia), elevated carbon dioxide levels (hypercapnia), and a highly restrictive substrate. Despite these harsh conditions, members of at least five mammalian families, spanning the placental orders Rodentia (Bathyergidae and Spalacidae), Afrosoricida (Chrysochloridae), and Eulipotyphla (Talpidae), together with the marsupial order Notoryctemorphia (Notoryctidae), are almost exclusively subterranean, while the families Cricetidae (Rodentia) and Chlamyphoridae (Cingulata) include subterranean genera (*Ellobius* and *Chlamyphorus*, respectively). Evolutionarily, these subterranean families have been highly successful, comprising 20–30 species each and showing wide geographical ranges (Reeder et al. 2007). While the Bathyergidae (African mole-rats) and Chrysochloridae (golden moles) occur only in Africa, members of the Spalacidae (spalacids) occur in Africa, Asia and Southern Europe, and the Talpidae (including the true moles) are found across the Northern Hemisphere (Reeder et al. 2007). Although African mole-rats and spalacids are both rodents, they represent two independent subterranean families.

All extant subterranean mammals have evolved broadly similar body plans for movement through tunnels, characterized by cylindrical bodies and shortened limbs, strong claws, the absence of pinnae and reduced eyes. Additionally, golden moles and spalacids possess tough nose pads, while African mole-rats, spalacids, and *Ellobius* show highly modified, enlarged incisors that they use for digging and feeding (Zuri et al. 1999). Adaptations for burrowing might include elastic skin, which in the naked mole-rat (*Heterocephalus glaber*, Bathyergidae), appears to be conferred by high-molecular-mass hyaluronan, a molecule also implicated in cancer resistance (Tian et al. 2013). Several subterranean taxa have developed highly derived sensory traits, including highly tactile noses in star-nosed moles (*Condylura cristata*, Talpidae) (Catania 1999) and degenerate eyes in both blind mole-rats (Spalacidae) and golden moles (Chrysochloridae) (Emerling and Springer 2014). Other documented adaptations in these groups relate to their putative ability to cope with hypoxia and hypercapnia, such as the insensitivity of naked mole-rats to certain pain stimuli (Park et al. 2008; Smith et al. 2011). The high carbon dioxide levels that often occur in underground burrows are equivalent to those experienced by some hibernating mammals, and many independent hibernating lineages have evolved functionally convergent amino acid motifs in the NaV1.7 sodium ion channel encoded by *SCN9A* (Liu, Wang, et al. 2014). Finally, as a consequence of living in confined close proximity, subterranean species may be expected to face strong selection pressures from pathogens.

Several previous studies have investigated parallel molecular evolution in subterranean mammals. These have revealed convergent evolution in genes relating to hypoxia between plateau zokors (Spalacidae) and naked mole-rats (Bathyergidae) (Shao et al. 2015), and comparisons of subterranean and closely related terrestrial rodents found significant differences in substitution rates of protein-coding genes between the two groups (Du et al. 2015). Additionally, two genome-wide screens for changes in evolutionary rates identified enrichment in vision- and skin-related loci (Prudent et al. 2016; Partha et al. 2017). However, the majority of these highlighted loci were thought to exhibit accelerated evolution due to relaxation of selective constraints, rather than adaptive evolution.

In this study, we therefore aimed to determine for the first time the extent to which multiple divergent groups of subterranean mammals have experienced adaptive substitutions in the same sets of loci. We analyzed genome-wide coding DNA sequences (CDSs) of representative species from four independent subterranean mammal lineages, representing three superorders; golden moles (Chrysochloridae: Afrotheria), true moles (Talpidae: Laurasiatheria), African mole-rats (Bathyergidae: Euarchontoglires), and spalacids (Spalacidae: Euarchontoglires). To determine the extent of parallel molecular adaptation associated with ecological transitions to life below ground, we first performed selection tests on focal branches of the four subterranean groups, then compared sets of genes under positive selection, and tested for enrichment in shared functions. Second, for each gene, we quantified the number of convergent amino acid substitutions shared between pairs of focal branches. By combining the results of these two tests, we mapped the co-occurrence of positive selection and convergent substitutions in each subterranean lineage to assess whether any of the observed shared substitutions could be defined as adaptive. To estimate levels of parallel evolution that might be expected to occur by chance, and not due to shared phenotypes, we repeated the above analysis for “control” groups of nonsubterranean taxa.

We predicted that parallel molecular adaptation in the focal lineages would be most evident in loci associated with the key features and/or challenges characterizing the subterranean niche. Thus, we examined our results in the context of the following selection pressures: 1) hypoxia and oxygen carriage, 2) low or absent light, 3) a restrictive substrate, and 4) exposure to pathogens. We hypothesized that sets of positively selected and convergent loci would be enriched for roles, or involvement in pathways, that pertain to these ecological and physiological challenges.

## Results

### Taxonomic Representation and Alignment Construction

**Fig. 1.**
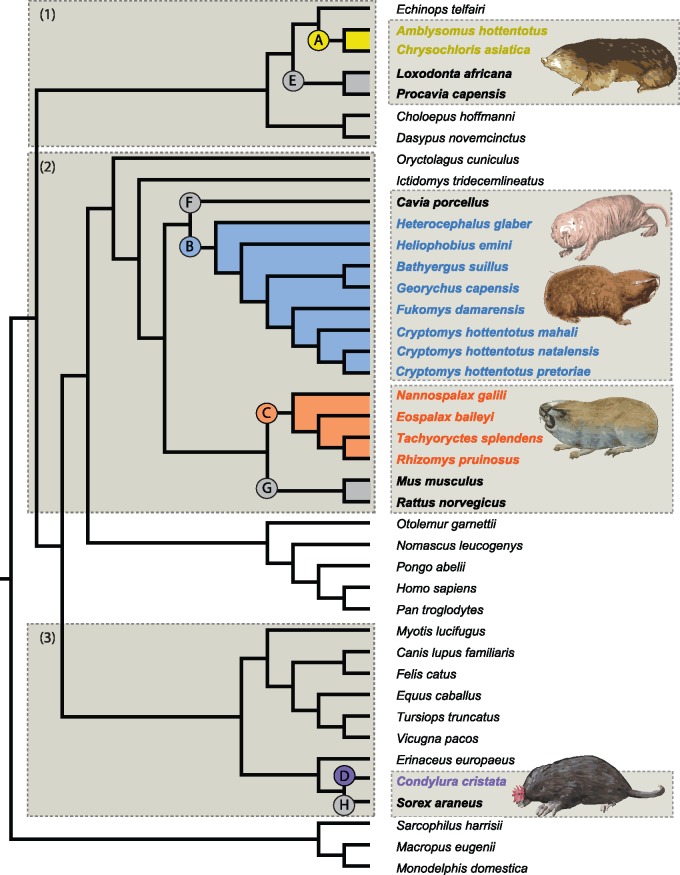
Species tree topology used for selection tests and inference of convergent sequence evolution. Shaded boxes (1–3) represent the pruned taxonomic data sets used for positive selection tests in each of the four focal subterranean clades (yellow—Chrysochloridae; blue—Bathyergidae; orange—Spalacidae; and purple—Talpidae). Foreground branches of interest (subterranean lineages) used for selection tests are labeled as follows: A—golden moles (Chrysochloridae); B—African mole-rats (Bathyergidae); C—spalacids (Spalacidae), and D—true moles (Talpidae); and nonsubterranean taxa used for comparisons of positive selection and convergent evolution are highlighted in bold font, and branches are labeled as follows: E—elephant + hyrax; F—guinea pig; G—mouse + rat, and H—common shrew. Images of representative subterranean species show the gross phenotypic convergence across divergent lineages.

We obtained genome-wide CDSs representing four independent groups of subterranean mammals (golden moles, African mole-rats, spalacids, and true moles, 15 species in total) and their nonsubterranean sister taxa (26 species). By performing de novo assembly of RNA-Seq data and combining this with published genomes/transcriptomes, we generated alignments representing >8,000 protein-coding genes in up to 41 species (fig. 1).

### Parallel Selection Pressures in Subterranean Lineages

#### Genes under Positive Selection

To test for site-specific positive selection in each of the four subterranean mammal lineages, we ran branch-site models (model MA) on ∼8,000 genes (fig. 1). Tests performed on the ancestral branch of each clade (represented by a single species of star-nosed mole, *C. cristata*, in talpid moles) revealed a total of 1,419 cases of positive selection, encompassing 1,267 positively selected genes (PSGs). Numbers of PSGs per focal branch ranged from 279 in Spalacidae to 483 in the star-nosed mole (table 1 andsupplementary tables S3–S6, Supplementary Material online). These numbers are comparable to the PSGs in the four control taxa also tested (303–763) (table 1, supplementary tables S7–S10, Supplementary Material online), there is minimal—though in some cases significant—overlap (<5%) between genes under positive selection in subterranean and nonsubterranean taxa (see supplementary fig. S4 and supplementary results, Supplementary Material online).

**Table 1.** Results of Branch-Site Models Used to Test for Positive Selection in Subterranean and Control Nonsubterranean Taxa.

Branch	Lineage Tested	∑ Alignments Excluded	∑ Alignments Retained for Branch-Site Test	PSGs *P *<* *0.05 [FDR *P *<* *0.05]	Enriched GO Terms (MF, CC, BP)
(A) Subterranean	Golden moles	441	7,870	356 [6]	32 (26), 24 (17), 114 (85)
(B) Subterranean	African mole-rats	236	8,075	301 [18]	39 (26), 21 (12), 132 (90)
(C) Subterranean	Spalacids	690	7,621	279 [11]	16 (16), 16 (13), 96 (82)
(D) Subterranean	Star-nosed mole (talpid)	799	7, 512	483 [37]	18 (15), 25 (23), 246 (161)
(E) Terrestrial	Elephant+hyrax	416	7,895	303 [32]	8 (8), 15 (11), 126 (88)
(F) Terrestrial	Guinea pig	920	7,391	742 [207]	38 (30), 40 (29), 248 (151)
(G) Terrestrial	Murids	185	8,126	462 [7]	30 (22), 36 (24), 154 (115)
(H) Terrestrial	Common shrew	993	7,318	763 [88]	36 (24), 22 (20), 110 (96)

Note.—From the initial 8,311 gene alignments, data were excluded from analyses if 1) *n* outgroup taxa = 0, 2) *n* focal taxa = 0, 3) ∑*n* taxa < 4, or 4) median interval of PSSs ≤ 10. Numbers of enriched GO terms reported are those detected with Fisher’s exact test using the classic algorithm, and numbers in brackets are those remaining significant under the elim algorithm.

**Fig. 2.**
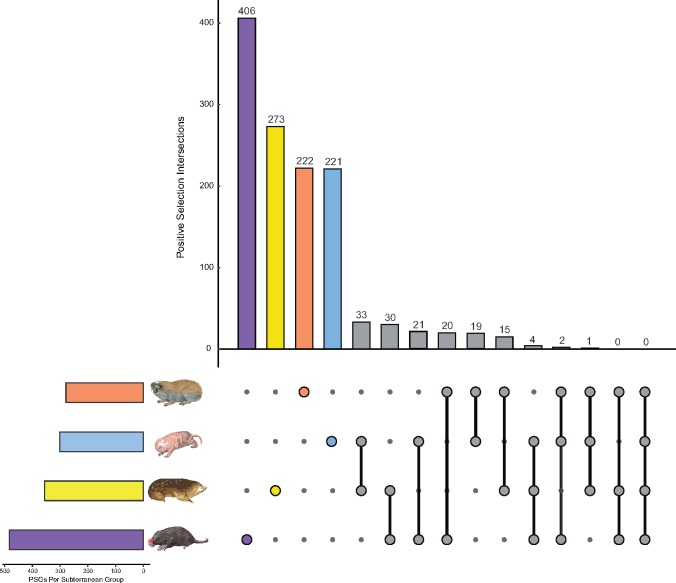
Number and overlap of PSGs in each of the four subterranean lineages, visualized as an UpSet plot. Subterranean lineages are represented as follows: yellow—Chrysochloridae; blue—Bathyergidae; orange—Spalacidae; and purple—Talpidae, and overlap in respective sets is indicated by gray symbols.

To determine the extent of parallel molecular adaptation among the four focal subterranean groups, we compared respective sets of PSGs and found that 145 loci were shared in at least two lineages (fig. 2), although none remained after applying a false discovery rate (FDR) threshold of 0.05. Of the 145 loci, none showed positive selection across all four lineages; however, seven loci (*ADGRE5, C3*, *GLS2*, *LCT*, *SLC29A3*, *SVIL*, and *TEX15*) showed positive selection in three lineages (supplementary fig. S5*A* and table S11, Supplementary Material online). A further 138 loci showed positive selection in any two subterranean groups, with this overlap spread relatively evenly among the different pairwise comparisons. Significance tests, using the supertest function (Wang et al. 2015), of these gene set intersections (against 6,536 background genes) revealed significant intersections between African mole-rats and spalacids (fold enrichment = 1.71, *P *=* *0.009), African mole-rats and golden moles (fold enrichment = 2.32, *P *=* *7.53 × 10^−7^), and African mole-rats and golden moles and the star nosed-mole (fold enrichment = 3.30, *P *=* *0.034) (supplementary table S12, Supplementary Material online).

Inspection of all 1,267 PSGs, including those shared by multiple groups, revealed many cases of molecular adaptation putatively associated with the key traits exhibited by subterranean mammals. Molecular adaptations related to living in low oxygen and/or high carbon dioxide level environments are suggested by >60 PSGs with known functions in either the vascular system and/or a response to hypoxic conditions (see Discussion for details). For example, *PARK7*, which may protect against reactive oxidative stress (Billia et al. 2013), was under positive selection in African mole-rats. Relatively few “sensory” genes were found to be under positive selection in multiple subterranean lineages; however, we detected positive selection in a number of sensory perception genes along the star-nosed mole branch, including genes with putative roles in vision (e.g., *CDHR1*, *IMPG2*, and *TTC8*), hearing (e.g., *ADGRV1, TECTB*, and *USH1C*), and sensory receptors (*TAS2R4*, *TRPV2*, and *PIEZO2*). Other PSGs in subterranean lineages included elastin-related genes which encode components of the extracellular matrix (e.g., *EMILIN1* and *ELANE*) (supplementary fig. S5*A* and tables S3–S6, Supplementary Material online). Life underground may present unique selection pressures from exposure to soil-specific pathogens, and many PSGs in the subterranean lineages are associated with the immune system (e.g., *C3*, *ITGA1* and *LGMN*). However, in the majority of cases, positive selection acting on the same gene across multiple lineages was not found (supplementary fig. S5*A*, Supplementary Material online).

Positive selection in many genes, or gene families, was not restricted to subterranean lineages (supplementary tables S7–S10, Supplementary Material online). For example, of the 17 genes previously linked to Fanconi anemia (and therefore, response to DNA damage) six and eight were found to be under positive selection in subterranean and nonsubterranean lineages, respectively. Similarly, multiple myosins, collagens and laminins were under positive selection in both subterranean and nonsubterranean taxa (see supplementary results, Supplementary Material online, for details of selection tests in nonsubterranean lineages).

#### GO Functional Enrichment of PSGs in Subterranean Lineages

For each set of genes identified as being under positive selection in each of the four subterranean lineages, we performed tests of gene ontology (GO) enrichment across three domains (molecular function [MF], cellular component [CC], and biological process [BP]). Numbers of enriched GO terms, based on Fisher’s exact test and the classic algorithm, broadly reflected those of PSGs in each clade, with Spalacidae having the fewest enriched GO terms across domains and the star-nosed mole typically the most (table 1 and supplementary tables S13–S16, Supplementary Material online).

We found limited evidence that enriched terms were shared among the focal subterranean groups, even when using the less conservative classic algorithm. Only the single BP term “Defense response to bacterium” was shared by all four groups, and seven BP terms—again largely immunity-related—were shared by three groups (supplementary fig. S5*B*, Supplementary Material online). In comparison, no MF or CC terms were enriched across all four lineages, although one MF term (“Myosin binding”) and six CC terms were enriched in three lineages (supplementary tables S13–S16 and fig. S5, Supplementary Material online). All four subterranean lineages showed enrichment in many BP terms relating to oxygen and blood supply (supplementary table S17, Supplementary Material online), and shared enriched BP terms included three relating to vascular endothelial growth factor—a molecule implicated in oxygen sensing—found in both the star-nosed mole and golden moles (supplementary fig. S5*B*, Supplementary Material online). Similarly, enriched CC and MF terms relating to the mitochondria or heme binding were detected, although these were not shared by lineages (supplementary tables S13–S16 and fig. S5, Supplementary Material online). Putative molecular adaptations for living in low light conditions were reflected by GO enrichment in BP terms associated with sensation, most prominently in the star-nosed mole—22 terms relating to sensory perception and/or detection of mechanical and light stimuli, compared with two such enriched BP terms in the common shrew (see supplementary results and supplementary table S21*C*, Supplementary Material online). Putative cases of molecular evolution related to skin adaptations for moving in a restrictive substrate may be reflected by enrichment in CC, MF, and BP terms associated with the basement membrane, extracellular matrix, and collagen (supplementary fig. S5 and tables S13–S16, Supplementary Material online). GO terms relating to an immune response were generally the most frequently detected enriched terms in both the subterranean (e.g., “Regulation of humoral immune response,” “Humoral immune response mediated by circulating immunoglobulin,” and “Lymphocyte-mediated immunity”) and nonsubterranean lineages examined (supplementary table S17, Supplementary Material online). The majority of these enrichments were also found by the elim and weight algorithms which take the GO topology into account (supplementary tables S13–S16, Supplementary Material online).

### Convergent Amino Acid Substitutions

#### Detecting Convergent Amino Acid Substitutions in Divergent Subterranean Lineages

As a second measure of parallel molecular evolution, we calculated posterior probabilities (PPs) of total convergent (i.e., parallel and convergent) amino acid substitutions between each of the six pairwise combinations of subterranean lineages. The numbers of proteins with PP total convergent amino acid substitutions (≥1.0—chosen as the lower threshold that may correspond to one convergent site) among pairs of lineages ranged from 210 between African mole-rats and spalacids (B vs. C) to 2,011 between golden moles and the star-nosed mole (A vs. D) (table 2 and supplementary table S22, Supplementary Material online). These numbers decreased to 183 and 1,968, respectively, when only loci with at least one individual site with PP > 0.5 were retained.

**Table 2.** Summary of the Number of Genes Tested for the Presence of Convergent Substitutions, and the Number of Genes with Summed PP ≥ 1.0 and 2.0 across Subterranean and Nonsubterranean Pairs.

Taxa Pair	No. Genes	No. PP ≥ 1.000 (No. genes 1+ sites PP > 0.5)	No. PP ≥ 2.000 (No. genes 2+ sites PP > 0.5)
Golden moles versus African mole-rats	7,486	506 (465)	125 (99)
Golden moles versus spalacids	7,085	627 (554)	155 (127)
Golden moles versus star-nosed mole	7,044	2,011 (1,968)	934 (902)
African mole-rats versus spalacids	7,186	210 (183)	40 (32)
African mole-rats versus star-nosed mole	7,120	735 (661)	239 (199)
Spalacids versus star-nosed mole	6,732	895 (791)	287 (228)
Elephant + hyrax versus guinea pig	7,622	675 (636)	175 (161)
Elephant + hyrax versus mouse + rat	7,673	648 (598)	161 (154)
Elephant + hyrax versus common shrew	7,889	1,096 (1,016)	344 (265)
Guinea pig versus mouse + rat	7,627	2,227 (2,168)	999 (959)
Guinea pig versus common shrew	7,295	3,004 (1,727)	1,597 (1,503)
Mouse + rat versus common shrew	7,337	3,129 (3,042)	1,645 (1,359)

**Fig. 3.**
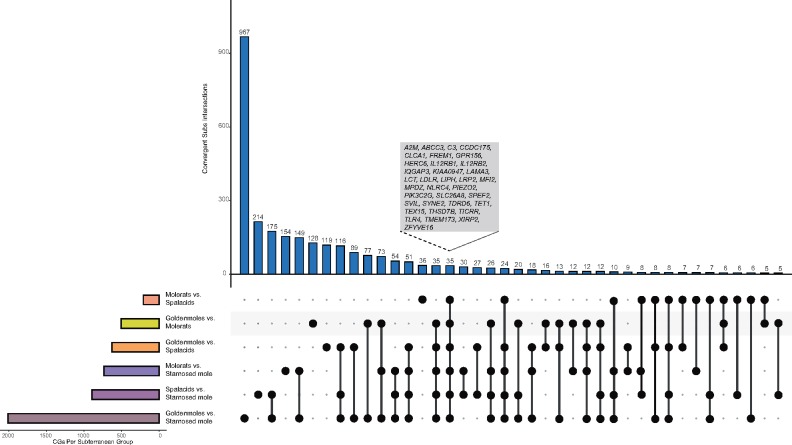
Number and overlap of genes identified as sharing convergent amino acid substitutions, summed PP of convergent substitutions ≥ 1.00, between pairs of subterranean lineages, visualized as an UpSet plot. The 35 “common” genes, identified across all six pairs, are highlighted by the gray box. CGs, convergent genes.

Thirty-five loci with PP ≥ 1.0 were common across all six subterranean pairwise comparisons (fig. 3). Of these loci, *FREM1* functions in craniofacial/renal development, *LAMA3* forms part of basement membrane, *Melanotransferrin* (*MFI2*) functions in iron cellular uptake, and *PIEZO2* forms a mechanosensitive ion channel (Safran et al. 2010). Additionally, at least three of the loci (*SLC26A8*, *TDRD6*, and *TEX15*) have roles in sperm development, and at least eight (e.g., *C3*, *CLCA1*, and *TLR4*) are involved with an immune response (Safran et al. 2010).

#### GO Functional Enrichment of “Convergent” Genes in Subterranean Lineages

GO enrichment analyses of the genes with >1.00 PP of convergence (supplementary tables S23–S25, Supplementary Material online) in each separate subterranean pair comparison yielded a total of 1,391 significant (*P* < 0.05) BP terms (627 unique). A total of 36 enriched BP terms were found to be shared across all six subterranean pairs (supplementary table S30*A*, Supplementary Material online). Of these, 29 were related to immunity/response to biotic stimulus, four were associated with either mitosis or meiosis, two were associated with “Protein activation cascade,” and finally both biological and cell adhesion were enriched. Across the remaining enriched terms, several shared by multiple pairs were linked to blood pressure/coagulation, including “Regulation of systemic arterial blood pressure by circulatory renin–angiotensin” was enriched across five pairs. “Monocarboxylic acid transport” was also enriched in five subterranean pairs. The MF term “Iron ion binding” was enriched in four pairs and “Heme binding” in three pairs. “Detection of external stimulus” was enriched in five of the subterranean pairs, and the following terms were enriched in at least half of the subterranean pair comparisons: “Response to radiation,” “Response to gamma radiation,” “Response to ionizing radiation,” and “Detection of visible light.” Several CC terms relating to the extracellular matrix were enriched across all six pairs (e.g., “Basement membrane”).

#### Detecting Convergent Amino Acid Substitutions in Control Comparisons

To obtain a measure of convergent substitutions that might be expected to occur between any given two taxa, and so to assess the biological significance of the detected subterranean convergence, we used control comparisons comprising either pairs of nonsubterranean taxa or nonsubterranean versus subterranean (mixed phenotype) taxa. As convergent substitutions can occur due to neutral processes, we assessed levels of convergence between pairs of taxa in relation to summed branch lengths (supplementary fig. S7, Supplementary Material online). Across all three groups (subterranean, nonsubterranean and mixed), we found a positive trend between branch length and number of estimated convergent amino acid substitutions (supplementary table S31 and fig. S7, Supplementary Material online). Detected levels of convergence in the subterranean taxa were similar to those of the mixed control group containing lineages with comparable branch lengths (the “relaxed” controls; see supplementary results, Supplementary Material online).

As above, the number of genes with evidence of convergent substitutions varied widely across nonsubterranean taxa from 3,129 in the mouse+rat versus common shrew (G vs. H) comparison to 648 in the elephant+hyrax versus mouse+rat (E vs. G) comparison (table 2 and supplementary fig. 8*A*, Supplementary Material online). In total, 4,683 unique genes with summed PP ≥ 1.00 were detected across the six control comparisons, and of these 130 were common to all six pairs, including *ALB*, *BRCA2*, and many (e.g., *IFNAR1*) relating to the immune system. In the mixed phenotype control group, the number of putative convergent genes varied from 2,193 in the star-nosed mole versus mouse+rat to 171 in African mole-rats versus elephant+hyrax, with 13 genes common to all six pairs (supplementary fig. 8*B*, Supplementary Material online). Three genes (*A2M*, *SYNE2*, and *TEX15*) had convergent substitutions across all pairs examined (supplementary fig. S8*C*, Supplementary Material online).

GO enrichment analyses of convergent genes (PP > 1.00 of total convergence) in the nonsubterranean control taxa yielded a total of 1,362 significant BP terms (671 unique terms). Fifteen BP terms were enriched across all six control pairs, including “Biological adhesion” and “Cell adhesion,” five terms relating to immunity, four terms relating to meiosis, and four terms relating to protein activation/processing (supplementary table S30*B*, Supplementary Material online). Across the six pairs, we also found enriched GO terms relating to blood coagulation and sensory perception (see supplementary results, Supplementary Material online). Therefore, assessment of these results suggests limited evidence for an excess of convergent amino acid substitutions between the subterranean lineages examined compared with any of the three control comparisons.

### Correlating Evidence of Convergent Evolution and Selection Pressures

**Fig. 4.**
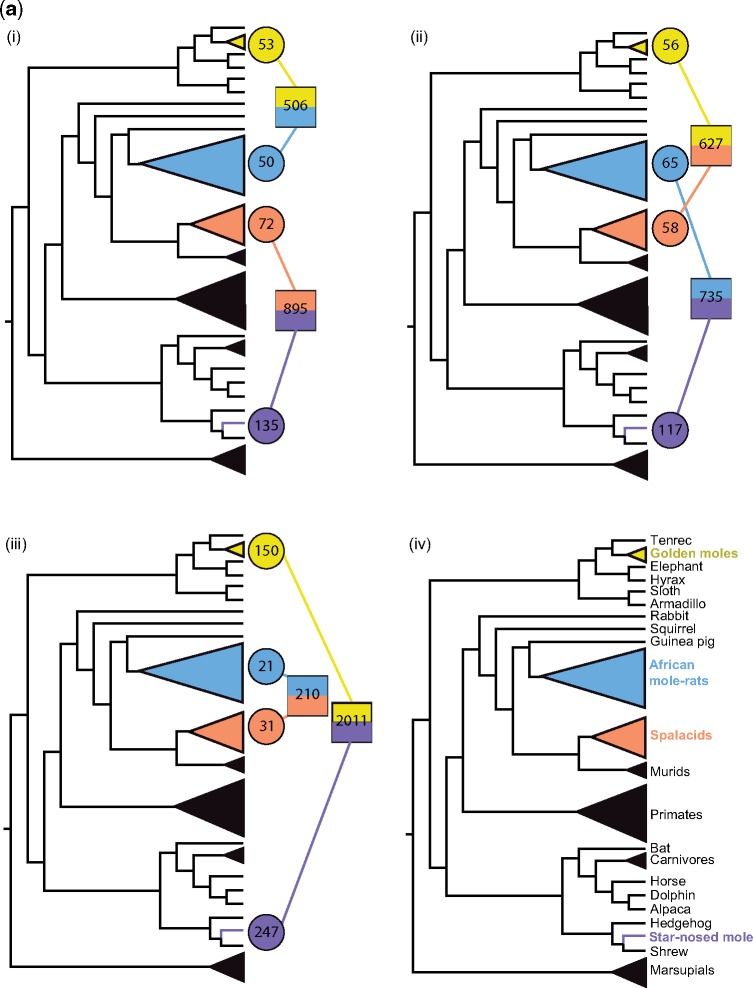

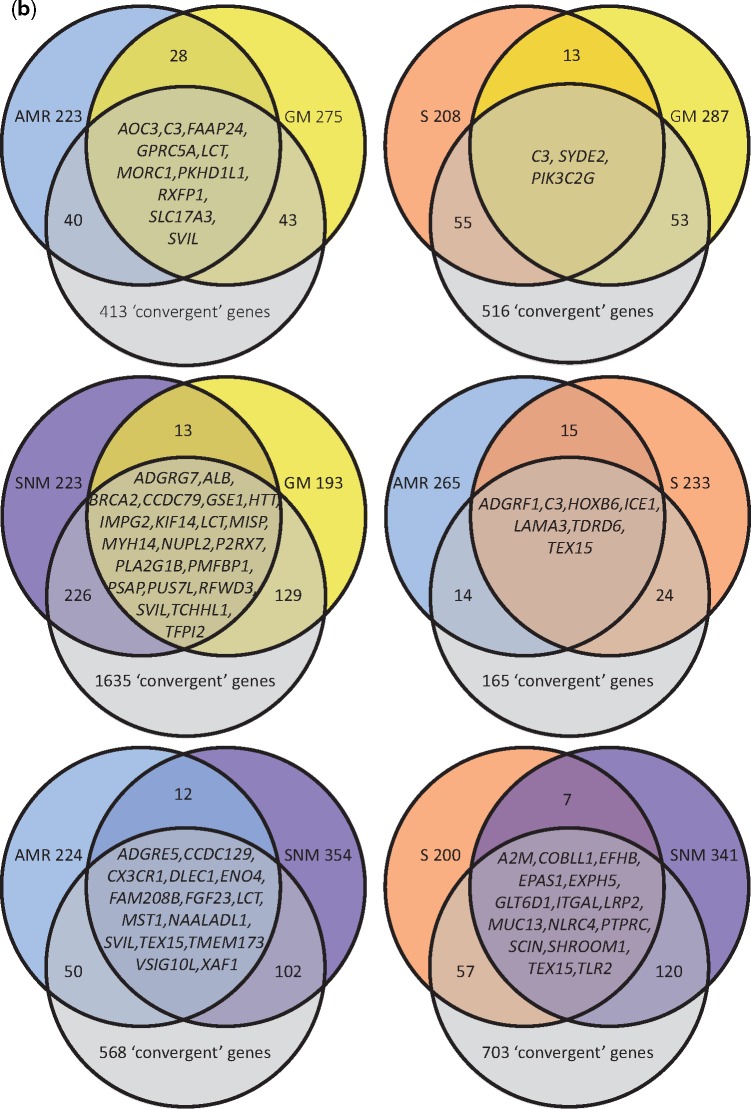
(*A*) Numbers of genes found to be under significant positive selection in subterranean taxa, and with summed PP of convergent substitutions ≥ 1.00 between the pairs of subterranean clades (i–iii), (iv) simplified species tree. Shaded circles denote genes under positive selection and shaded squares denote genes with convergent substitutions; colors represent subterranean taxa/clades as follows: yellow—Chrysochloridae; blue—Bathyergidae; orange—Spalacidae; and purple—Talpidae. (*B*) Venn diagrams representing overlap between genes under positive selection in two subterranean lineages and the presence of convergent substitutions shared between these two lineages. Positive selection in each subterranean lineage is colored as following: yellow—Chrysochloridae; blue—Bathyergidae; orange—Spalacidae; and purple—Talpidae. Gray circles represent the number of genes with convergent substitutions between each respective pair of taxa.

We compared the results of positive selection and estimates of convergent substitutions to determine in which genes inferred amino acid changes might be considered adaptive. Overall, a small proportion of genes (0.3–1.1%) had both a summed convergence PP ≥ 1.00 and were found to be under positive selection in both lineages across the six subterranean pairs (fig. 4). The 71 loci in subterranean pairs contained 63 unique genes and four genes (*C3*, *LCT*, *SVIL*, and *TEX15*) found in multiple pairs. These proportions are equivalent to the numbers found in “mixed controls” (0.5–1.4%, supplementary fig. S10, Supplementary Material online) with 85 occurrences (72 unique genes). Additionally, 17 common genes (*A2M*, *ALB*, *BRCA2*, *C3*, *DLEC1*, *EXPH5*, *IMPG2*, *ITGAL*, *LCT*, *MYH14*, *P2RX7*, *PKHD1L1*, *SHROOM1*, *TCHHL1*, *TDRD6*, *TEX15*, and *VSIG10L*) were found to be under both positive selection and show evidence of convergence in both subterranean and the “mixed control” pairs (see fig. 4 and supplementary fig. S10, Supplementary Material online). We further explored the 63 genes found in subterranean pairs to determine whether the same sites were under positive selection and convergent; of these, 18 contained at least one site under both positive selection (≥1 subterranean lineage) and one site deemed to be a convergent substitution (table 3 and supplementary table S32, Supplementary Material online). These genes included *CX3CR1*, *EPAS1*, and *FAAP24*. In comparison, of the 72 genes in the control pairs, 18 contained convergent sites under positive selection, although in eight of these genes the convergent sites were under positive selection only in the subterranean lineage (table 3 and supplementary table S33, Supplementary Material online).

**Table 3.** Amino Acid Sites Identified as Both Convergent (PP > 0.5) and under Positive Selection between Subterranean Species Pairs, and Subterranean and Nonsubterranean Species Pairs.

Gene	Taxa Pair	“Convergent” Site	PSS Lineage 1	PSS Lineage 2
*ALB*	SNM versus GM	236	NA	236
*CX3CR1*	SNM versus AMR	56	NA	56
*EPAS1*	SNM versus Sp	696	NA	696
*FAAP24*	AMR versus GM	30	NA	30
*FAM208B*	SNM versus AMR	1581	NA	1581
*FGF23*	SNM versus AMR	106	NA	106
*GLT6D1*	SNM versus Sp	100, 116, 151, 222, 224	100, 116, 222, 224	116, 151, 222
*GPRC5A*	AMR versus GM	211	211	211
*HOXB6*	AMR versus Sp	61	61	61
*HTT*	SNM versus GM	1847	1847	1847
*MYH14*	SNM versus GM	1511, 1654	1511, 1654	1654
*SCIN*	SNM versus Sp	201	NA	201
*SVIL*	AMR versus GM	1310	1310	NA
*SVIL*	SNM versus AMR	1518	NA	1518
*TDRD6*	AMR versus Sp	1416, 1420	NA	1416, 1420
*P2RX7*	SNM versus GM	344	NA	344
*PSAP*	SNM versus GM	88	88	88
*PTPRC*	SNM versus Sp	524	524	NA
*PUS7L*	SNM versus GM	621	621	NA
*ENTPD1*	SNM versus E+H	279, 293	NA	279, 293
*GPR128*	SNM versus E+H	376	NA	376
*LPCAT2*	SNM versus E+H	244	NA	244
*P2RX7*	SNM versus E+H	192	NA	192
*PLXNC1*	SNM versus E+H	111	NA	111
*ACACB*	CS versus AMR	1220	NA	1220
*KIAA1217*	CS versus AMR	463	NA	457, 463
*LCT*	CS versus AMR	1671	NA	1671
*NTN5*	CS versus AMR	67, 119	NA	67, 119
*PKD1L1*	CS versus AMR	1023	NA	1023
*TICAM1*	CS versus AMR	289	NA	289
*CCDC175*	M+R versus SNM	569	569	569
*LAX1*	M+R versus SNM	267	267	267
*LRRC63*	M+R versus SNM	97	NA	97
*TTF2*	M+R versus SNM	119	NA	119
*KIAA0947*	Sp versus E+H	883	NA	883

Note.—Only amino acid sites identified as under positive selection and convergent are shown—see Supplementary Material online for all sites. Site number refers to position in trimmed alignment. PSS based on likelihood ratio test *P* < 0.05; ω > 1.00 and Bayes Empirical Bayes >0.5. PP, posterior proportion; NA, not applicable; SNM, star-nosed mole; GM, golden moles; AMR, African mole-rats, Sp, Spalacids; E + H, elephant+hyrax; CS, common shrew; M + R, mouse+rat.

## Discussion

We aimed to assess the evidence for parallel molecular adaptive evolution among divergent lineages of subterranean mammals. Genomic approaches have been increasingly used to reveal the molecular bases of mammalian phenotypic evolution although few studies have examined taxa that have independently colonized similar habitats. Such comparisons might increase the power to identify important loci, as well as offer the opportunity to assess the extent to which the same sets of loci and pathways have been recruited in divergent yet ecologically and phenotypically similar taxa.

We tested for molecular adaptations in protein-coding genes across four lineages of subterranean mammal lineages, including the star-nosed mole and two genera of golden moles. While African mole-rats and blind mole-rats (spalacids) have been studied extensively in relation to resistance to oxidative stress, toxin-resistance, and circadian biology (Rado et al. 1991; Oosthuizen et al. 2003; Fang, Nevo, et al. 2014; Fang, Seim, et al. 2014; Schmidt et al. 2017), other major groups of subterranean mammals have so far been largely overlooked. For example, studies of molecular evolution in true moles (talpids) and golden moles have been restricted to loci associated with the visual system (Carmona et al. 2008; Emerling and Springer 2014), and thus virtually nothing is known about the genetic basis of other phenotypic changes in these taxa.

**Fig. 5.**
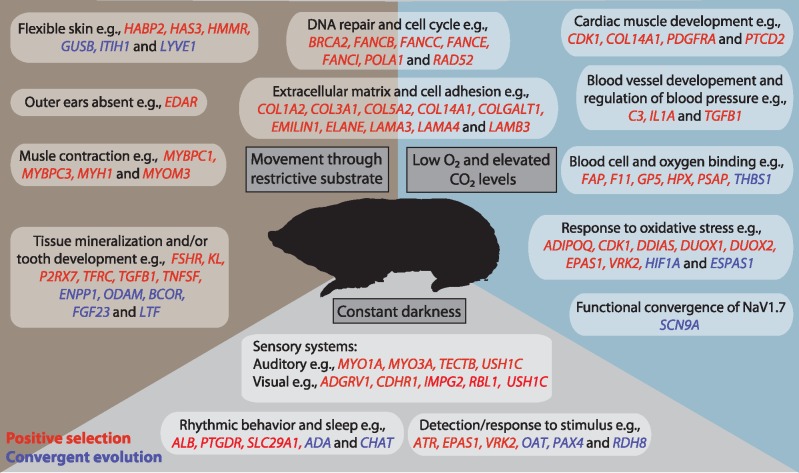
Illustration of three of the dominant environmental pressures acting on subterranean mammals and the possible molecular adaptations evolved in response to these pressures. Listed genes are colored as follows: red—under positive selection in at least one subterranean lineage; blue—total PP of convergence ≥ 1.00 between at least one pair of subterranean taxa.

Our analyses based on ∼8,000 genes across four subterranean mammal lineages revealed positive selection in over 1000 loci across the entire sample. Yet we found that just ∼11% of these PSGs were under selection in more than one lineage, with few (<1%) under selection in three lineages, and none in all four. Furthermore, these PSGs are based on nominal *P*-values (<0.05) following filtering for potential low-quality alignments, and, therefore, the low numbers of shared PSGs can be considered moderate values. A similar trend was observed for convergent analyses; only a fraction of loci containing putative convergent amino acids (based on pairwise PPs) were identified in multiple pairwise comparisons. Comparing our results from tests for positive selection and convergence revealed little overlap in terms of gene identity, and co-occurrence of these two forms of molecular evolution at the same site within a locus was rare (<0.5% of loci tested). A lack of evidence of parallel molecular evolution among the four focal lineages, in terms of either positive selection or convergence, may suggest that their adaptations to conditions underground have arisen via either predominantly divergent genetic routes or alternative (e.g., noncoding) molecular mechanisms. Consistent with this scenario we found numerous cases of molecular adaptation—often involving functionally related genes—across these taxa, and provide some of the first insight into molecular adaptations relating to fossoriality in talpid and golden moles (summarized in fig. 5). It has previously been suggested that there may be three origins of fossoriality within Spalacidae (Flynn 2009); while this could account for limited evidence of parallel evolution occurring along the ancestral spalacids branch, it would not explain the low levels observed among the other groups.

### Molecular Adaptations in Subterranean Mammals

Subterranean mammals inhabit hypoxic burrows with elevated carbon dioxide levels, and therefore it might be expected that these animals were under strong selection to evolve molecular mechanisms for effective oxygen sensing and delivery. In line with these predictions, positive selection was detected in *HPX* in two subterranean lineages; the serum glycoprotein encoded by this gene binds to heme with high affinity and protects tissues from oxidative reactions catalyzed by free heme (Barnard et al. 1993; Dong et al. 2013). Additionally, we identified one convergent site each in two genes (*HIF1A* and *EPAS1*) that function in the homeostatic response to hypoxia (Tian et al. 1997), shared between golden moles and, respectively, the star-nosed mole and spalacids. The latter gene was also found to be under positive selection. Our results therefore support previous reports of parallel evolution in *EPAS1* between subterranean mammals (Shao et al. 2015). Interestingly this gene is differentiated in human populations living at high elevations (Yi et al. 2010) and therefore could represent a candidate for future functional studies. GO enrichment analyses of PSGs further highlighted adaptation in the response to hypoxia; three enriched terms related to vascular endothelial growth factor production, a molecule controlling vascularization that is upregulated in low oxygen (Gerber et al. 1997).

A putative molecular adaptation to hypercapnia was evident from the presence of a negatively charged motif (EKD) in the Na_V_1.7 sodium ion channel of the star-nosed mole. The Na_V_1.7 sodium ion channel controls the action potential of pain receptors sensitive to acidosis, a consequence of chronic exposure to elevated carbon dioxide (Smith et al. 2011). Amino acid substitutions in this motif have previously been shown to be under functional convergence in some hibernating and burrowing mammals that face similar challenges (Smith et al. 2011; Liu, Wang, et al. 2013). Curiously, we found no such negatively charged motif in the golden moles—which were found to possess the KKA motif (data not shown)—implying that these taxa might have evolved alternative mechanisms to the same stressors.

In recent years, there has been considerable interest in African mole-rats (Bathyergidae) and spalacids (Spalacidae) due to reported low rates of cancer (Manov et al. 2013; Delaney et al. 2016). This apparent cancer resistance in naked mole-rats (Bathyergidae) may be due to substitutions in *HAS2* (*Hyaluronan synthase 2*), which results in a high molecular mass form of hyaluronic acid (Tian et al. 2013). It has been speculated that these molecular changes have arisen due to selection for elastic skin (Tian et al. 2013), a requirement when burrowing in restrictive substrates. While we did not detect positive selection in hyaluronic acid associated genes in either spalacids or African mole-rats, positive selection was detected in three associated genes in golden moles (*HABP2*, *HAS3*, and *HMMR*); these also led to enrichment in associated GO terms (see Supplementary Material online). Interestingly, enrichment in GO terms relating to hyaluronic acid was also found in some control comparisons based on convergent amino acid changes. Perhaps unsurprisingly, given the vital function of the extracellular matrix in virtually all structures (Mouw et al. 2014), we found many genes that produce the constitutive molecules (e.g., collagens, proteoglycans, laminins, and fibronectin) to be under positive selection in some of the subterranean lineages. These results contrast with a recent study that did not detect accelerated evolution in laminins (Partha et al. 2017). However, our results, and those of Partha et al. (2017), provide evidence of potential adaptation in loci putatively associated with thickened skin, with positive selection in subterranean lineages detected in the loci *COL14A1* and *COL5A2*, which have been linked to the skin diseases punctate palmoplantar keratoderma type I and classical Ehlers–Danlos syndrome. Symptoms of the former include thickened skin on the palms and soles, and those of the latter include hyperextensible skin (Rappaport et al. 2013). Genes relating to another structural component, myosin were also found to be under positive selection across subterranean taxa, including sensory, cardiac, and structural myosins (Grewal et al. 1999; Safran et al. 2010). However, positive selection relating to extracellular matrix components and myosins was also found in control taxa, and therefore specific associations with the subterranean niche remain unclear.

In this study, we detected no common signals of either positive selection or convergence in sensory perception genes across the subterranean lineages. This contrasts with previous studies that found parallel signals of relaxation in genes relating to the visual systems of subterranean mammals (Emerling and Springer 2014; Partha et al. 2017). This may relate to the fact that, while subterranean mammals share degeneration of vision and audition (Heffner RS and Heffner HE 1993), their other senses may be under divergent selection pressures in each of the subterranean clades. For example, the star-nosed mole has a highly tactile nose, which may serve as the principal sensory input in this species, while olfaction has been shown to be enhanced in other subterranean species (Stathopoulos et al. 2014). In general, the most cases of positive selection associated with hearing, vision, taste, touch and temperature detection and BP enrichment for sensory terms (∼22 terms) were found in the star-nosed mole, perhaps reflecting the fact that this species is the least subterranean of the four clades studied here. Independent subterranean mammal lineages are known to have lost the ability to detect high-frequency sounds and have poor sound localization (Heffner RS and Heffner HE 1993; Heffner et al. 2014), yet at the same time some lineages exhibit auditory adaptations which may enable improved sensitivity to seismic vibrations (Mason 2006). In addition to several putative hearing genes, including several myosins, we also found positive selection in *EDAR* in African mole-rats (also see Davies et al. [2015]), and polymorphisms in *EDAR* have been linked to variable pinna shape in humans (Adhikari et al. 2015). Despite these results, we acknowledge that the alignments used by this study contain some sequences from mixed-tissue transcriptomes and are based on one-to-one orthologs and therefore will have excluded some genes that are present as large gene families (e.g., olfactory receptors) as well as those that have highly specific expression in tissues that were not sampled, such as sensory organs. Thus, further research adopting alternative strategies has the potential to uncover further novel molecular adaptations in these loci. Widening the taxon sample would also be valuable, in particular by including marsupial moles and additional true moles from Talpidae, the latter of which was represented by a single species in our study and thus necessitated that associated model estimates for this lineage came from a “tip” rather than ancestral branch.

Across all subterranean species, we found positive selection, convergent substitutions, and GO enrichment in many genes/terms relating to immunity and reproduction. At the same time, however, similar or even higher numbers of loci and terms with related function and such signatures were detected in the nonsubterranean taxa, and thus we found no evidence of high selective pressures in the immune system in taxa that live underground. Archer et al. (2017) recently discussed the contrasting nature of selection on the immune system in taxa that live above versus below the ground, with potentially less diversity in parasites in the latter, while experiments on subterranean and nonsubterranean rodents have revealed contrasting susceptibility to viruses (Artwohl et al. 2009). The lack of a clear signal relating in our data to immunity might therefore be unsurprising and fit with previous suggestions that functional enrichment in terms relating to biological processess such as immunity and reproduction will be detected in all genome-wide selection scans (Yang and dos Reis 2011).

### Genome-Wide Studies of Convergence

Although we found evidence of parallelism in multiple genes, the numbers of loci were relatively small as a fraction of those examined. Thus, our results suggest these four groups of subterranean taxa have arrived at broadly similar ecologies and phenotypes through independent or alternative genetic routes. The small overlap in gene sets showing positive selection and convergence might also reflect differences among these divergent taxa, rather than similarities. For example, although all live underground, African and spalacid mole rats feed primarily on plant tubers, whereas the golden moles and star-nosed mole hunt arthropods and small vertebrates. Furthermore, some lineages are solitary whereas others are highly social, and anatomical differences are also evident; while golden moles and blind mole rats possess subcutaneous eyes and never go above ground, the star-nosed mole has reduced but functional eyes and may forage above ground and in aquatic environments. More generally, we cannot rule out the possibility that our selection tests failed to detect ancient bursts of molecular adaptation, especially on long branches (e.g., *Condylura*), or that positive selection has occurred since the transition to fossoriality and would thus be overlooked in tests of ancestral branches. Conversely, it is likely that cases of positive selection on focal branches may not relate to fossoriality itself.

While some previous studies concerning the identification of loci underlying convergent traits had obvious candidates (e.g., audition genes in relation to echolocation [Parker et al. 2013]), arguably, it is difficult to predict a priori which loci may show convergent evolution in subterranean mammals. The genetic bases of many of the morphological traits shared by subterranean mammals, for example, pinnae loss or cylindrical body shape, are not known and are likely controlled by multiple loci (Adhikari et al. 2015). Arguably the best candidate loci pertain to the visual system, and these have been extensively studied in subterranean mammals (Emerling and Springer 2014; Prudent et al. 2016; Partha et al. 2017). More generally, the extent to which phenotypic convergence can be related to sequence convergence is debated (Parker et al. 2013; Foote et al. 2015; Zhou et al. 2015), and empirical evidence supporting the functional significance of detected convergent substitutions is rarely available (Liu, Qi, et al. 2014). Furthermore, the optimal approach to quantifying parallel signatures of selection across genomes remains equivocal. For example, while our PSGs were identified using a conservative approach (Zhang et al. 2005), our interpretation of these and downstream analyses are based on genes identified with nominal *P*-values (0.05), an approach which has been adopted by several other studies (Foote et al. 2015; Sahm et al. 2017). Although post hoc corrections are often applied where lineage-specific selection tests are repeated for many loci, and we report these corrections, we have not focused on these due to our aims of detecting broad patterns of parallelism based on combining information across several gene sets.

We cannot rule out that other forms of parallel molecular evolution have occurred in subterranean taxa that involve neither positive selection nor shared amino acid substitutions. Cases of accelerated or decelerated evolutionary rates, pseudogenization, and parallel changes in gene expression have all been identified (Emerling and Springer 2014; Berens et al. 2015; Springer et al. 2016; Chikina et al. 2016; Partha et al. 2017). Previous studies have highlighted significant differential expression in genes involved in response to hypoxia, DNA damage, and oxido-reduction between nonsubterranean rodents and either spalacids or African mole-rats (Yu et al. 2011; Fang, Nevo, et al. 2014; Malik et al. 2016), and this remains another route of possible parallel adaptation in subterranean mammals (see Schmidt et al. [2017]). Improvements in genome assembly, annotation methods, and the availability of comparative data sets will make these studies possible in the near future.

## Materials and Methods

### Subterranean Mammal Species Representation and Data Sets

Our data set consisted of representative species from four independent acquisitions of a subterranean lifestyle: Chrysochloridae (*n *=* *2), Bathyergidae (*n *=* *8), Spalacidae (*n *=* *4), and Talpidae (*n *=* *1) (supplementary table S1, Supplementary Material online, and fig. 1). We generated RNA-Seq data for the golden mole *Amblysomus hottentotus*; total RNA was extracted and pooled from brain, liver, lung, skeletal muscle, heart, and kidney tissue samples by BGI (Hong Kong), which also performed 2 × 100 base-pairs (bp) paired-end sequencing with an Illumina HiSeq2000 (Illumina, San Diego, CA). Raw reads containing adaptors, unknown nucleotides >5%, and low-quality reads (i.e., >20% bases quality scores <10) were removed leaving 17,358,151 read-pairs (GenBank SRA: SRP126619). Transcriptome assembly was carried out with Trinity.r2013-02-25 (Grabherr et al. 2011) with default settings. This data set was combined with *Chrysochloris asiatica* CDS downloaded from GenBank. We combined RNA-Seq data assembled for a previous study (Davies et al. 2015) with genome-wide CDS data from the naked mole-rat (Keane et al. 2014) to obtain an African mole-rat data set representing all six genera. The four spalacids consisted of previously assembled RNA-Seq data—*Tachyoryctes splendens* (Davies et al. 2015), preassembled CDSs—*Spalax galili* (Malik et al. 2011), and downloaded short-reads—*Rhizomys pruinosus* and *Eospalax baileyi* (Lin et al. 2014). Finally, CDS gene predictions were downloaded from the star-nosed mole (*C. cristata*) genome. De novo transcriptome assembly of downloaded reads was carried out as above (supplementary table S2, Supplementary Material online). We searched the de novo transcriptomes for *trans*-self-chimeric transcripts following Yang and Smith (2013). Briefly, assemblies were queried against the human protein database (containing 23,393 proteins, Ensembl 75 [Flicek et al. 2014]) using BlastX v.2.2.29+ with an *e*-value cutoff of 0.01. High-scoring segment pairs meeting default identification parameters (identity >30% and >100 bp) were cut into segments and kept if >100 bp (for details see Davies et al. [2015]). Transcriptome completeness (based on conserved ortholog content) was assessed with BUSCOv.3 (Waterhouse et al. 2018) (see Supplementary Material online).

### Protein-Coding Gene Annotation of De Novo Assemblies

A reciprocal Blast approach was used to annotate protein-coding genes in each of the 15 subterranean mammal data sets. First, the longest sequence per locus for each human protein (Ensembl 75) was used as queries against each subterranean mammal database, using TBlastN v.2.2.29+. Only the top hit, with an *e*-value cutoff <1e^−6^, was kept. Second, reciprocal BlastX searches were conducted with transcripts used as queries against the human protein databases with only the top hit retained when *e*-value <1e^−6^. Percentage coverage of each hit was calculated with analyze_blastPlus_topHit_coverage.pl (Trinity utils). Candidates were then filtered by 9,034 one-to-one (across human, guinea pig, elephant, mouse, and common shrew) orthologous protein-coding genes from Ensembl 75.

### Mammal-Wide Alignment Construction

One-to-one canonical coding sequences for 26 mammal species were downloaded via the Ensembl-API, sequences with >50% missing data (*N*) were removed. These sequences were combined with the subterranean mammal transcripts, providing a maximum of 41 species per gene. Sequences were screened for incomplete codons; if applicable *N*s were added to complete the final codon. Sequences containing internal stop codons or sequences <150 nt (likely to cause alignment errors) were removed. Sequences were aligned with GUIDANCEv.1.41 (Penn et al. 2010) and PRANK (Löytynoja and Goldman 2005) with codons enforced and ten bootstraps. Sequences with a GUIDANCE score <0.6 were removed, and the alignment process repeated. Columns scoring <0.93, and/or >50% gaps, were removed with a Perl script. Sequences <150 nt (50 codons) were removed, and only alignments >100 codons were kept. Only alignments containing species from ≥2 origins of subterranean lifestyle were kept—resulting in a data set of 8,311 nuclear genes (∼92% of one-to-one orthologous protein-coding genes: human, guinea pig, elephant, mouse, and common shrew).

### Testing for Positive Selection Associated with the Subterranean Life

To identify putative genes involved in the adaptive evolution of mammals as they transitioned to a subterranean lifestyle, we tested each gene for positive selection along the branch leading to the inferred origin of underground life in our focal clades (fig. 1). For the four groups of interest (mole-rats, golden moles, spalacids, and star-nosed mole), alignments were pruned to include a single subterranean clade and closely related nonsubterranean sister taxa (i.e., mole-rats plus 1–5 Glires; spalacids plus 1–5 Glires; golden moles plus 1–3 Afrotheria and 1–2 Xenarthra; and star-nosed mole plus 3–8 Laurasiatheria). Alignments with fewer than four species were removed. We repeated selection tests on four terrestrial control branches (branches E–H, fig. 1). Sister taxa were chosen as controls to minimize effects of life-history traits on substitution rates, while accounting for genome quality, sampling completeness, and divergence levels.

We implemented branch-site models with codeML in PAML v.4.7. (Zhang et al. 2005; Yang 2007) to test for positive selection along each of the four branches of interest. In Model A, a branch is designated as the foreground branch, and sitewise estimates of ω (ratio of nonsynonymous to synonymous rates) are estimated separately for this focal branch and across the remaining background branches. Model A estimates four site classes: 0<ω_0_<1, ω_1_=1, ω_2a_ can exceed 1 on the foreground but constrained to purifying selection on the background, and ω_2 b_ can exceed 1 on the foreground but not on the background. Model A is then compared with the null Model A using the likelihood ratio test, and the significance of model fit assessed by chi-square test (1 degree of freedom), *P*-values <0.05 indicates the significantly better fit of the alternative model. The use of this test has been shown to produce conservative *P*-values (Zhang et al. 2005). The species tree topology follows previous studies (Blanga-Kanfi et al. 2009; Meredith et al. 2011; Davies et al. 2014, 2015; Lin et al. 2014). To reduce potential false positives due to alignment errors, we removed genes with highly aggregated positively selected sites (PSSs) (see Davies et al. 2015; Tsagkogeorga et al. 2015). Briefly, the median interval between PSSs is calculated and alignments removed if median interval is ≤10 amino acids. Downstream analyses were performed on significant gene sets (*P *<* *0.05) filtered by median interval PSS. We report FDR (Benjamini and Hochberg 1995)-adjusted Model A results for completeness, but do not use these adjusted values because our selection tests are based on a priori hypotheses to determine which genes are under selection in the four subterranean lineages, and because we are interested in broad patterns of parallelism. Overlap in PSGs was visualized with UpSet plots (Lex et al. 2014), and significance of observed intersections, calculated using expected intersections given the background of genes tested, examined with the supertest function from SuperExactTest v.0.99.4 (Wang et al. 2015). To assess enrichment in PSGs, we used Fisher’s exact test in topGO (Alexa and Rahnenfuhrer 2010) implemented in R v.2.15 (R Core Team 2013). For the reason that small sets of loci preclude meaningful tests of enrichment, we identified our gene sets based on PSGs with *P *<* *0.05. GO term accession codes and domains were downloaded from Ensembl. Fisher’s exact test was used to identify significantly overrepresented gene sets in combination with the classic, elim, and weight algorithms (*P *<* *0.05). Following recommendations by the authors, we did not apply FDR adjustment to the classic algorithm values to avoid being overly conservative, while the latter two algorithms take the GO topology into account and therefore the tested terms can be considered nonindependent (Alexa and Rahnenfuhrer 2010).

### Analyses of Convergent Sequence Evolution

To identify convergent amino acids shared between lineages of subterranean mammals, we estimated PPs of substitutions inferred from ancestral sequence reconstructions with codeML-ancestral (Castoe et al. 2009). This method calculates summed PPs of divergent amino acid substitutions between branch pairs as well as providing sitewise PP information. We used the Dayhoff substitution matrix, the species tree topology (fig. 1), with branch lengths and model parameters estimated using PAML v.4.7 (Yang 2007). To remove alignments containing possible errors, which may bias convergence probabilities, we discarded the 594 alignments that had scored a PSS median interval ≤10 amino acids. For each pair of interest, the PP of convergence was obtained for the most ancestral pairwise comparison possible. We then tested for enrichment in GO terms in the genes calculated with a summed probability of total PP convergence >1.00 using topGO as described above.

## Supplementary Material

Supplementary Data
