# Supplementary material for: Limited Evidence for Parallel Molecular Adaptations Associated with the Subterranean Niche in Mammals: A Comparative Study of Three Superorders
Source: Mol Biol Evol. 2018 Aug 20;35(10):2544–59. doi: 10.1093/molbev/msy161 (PMC6188548; doi:10.1093/molbev/msy161)
Supplement: Supplementary Data [file msy161_supp.zip › Davies_et_al_Supplementary_Material.pdf]

SUPPLEMENTARY MATERIAL

Limited evidence for parallel molecular adaptations associated with the subterranean niche in mammals: a comparative study of three superorders

Kalina T. J. Davies, Nigel C. Bennett, Chris G. Faulkes, Stephen J. Rossiter

Supplementary Materials and Methods

(1) Subterranean mammal species representation and datasets

Our dataset consisted of representative species from four independent acquisitions of a subterranean lifestyle: Chrysochloridae (golden moles) *n*=2; Bathyergidae, (African mole-rats) *n*=8; Spalacidae (spalacids) *n*=4 and Talpidae (true moles) *n*=1 (see supplementary table S1 and fig. 1). We generated RNA-Seq data for the golden mole *Amblysomus hottentotus*. Total RNA was extracted and pooled from brain, liver, lung, skeletal muscle, heart and kidney tissue samples by BGI (Hong Kong), which also performed paired-end sequencing, with a read length of 2 x 100 base-pairs, on an Illumina HiSeq2000 platform (Illumina, San Diego, USA). Raw reads containing adaptors, unknown nucleotides >5% and low quality reads (i.e. >20% bases quality scores <10) were removed leaving 17,358,151 clean read pairs (Q20 94.64% and GC 48.68%). Clean reads have been deposited in the GenBank Sequence Read Archive (SRA) under SRA Study accession SRP126619. *De novo* transcriptome assembly was carried out using the default settings of Trinity (release 2013-02-25) (Grabherr et al. 2011). This dataset was combined with coding DNA sequence (CDS) predictions from the *Chrysochloris asiatica* genome downloaded from GenBank. We included published RNA-Seq data assembled for a previous study (Davies et al. 2015) combined with genome-wide CDS data from African mole-rat species representing all six genera (Keane et al. 2014). The four spalacids included in our study consisted of published RNA-Seq data – *Tachyoryctes splendens* (Davies et al. 2015), pre-assembled CDSs – *Spalax galili* (Malik et al. 2011) and downloaded short-reads – *Rhizomys pruinosus* and *Eospalax baileyi* (Lin et al. 2014). Finally, CDS gene predictions were downloaded from the star-nosed mole, *Condylura cristata* genome. *De novo* transcriptome assembly of downloaded reads was carried out as above (see supplementary table S2 for assembly statistics). We searched the three *de novo* transcriptomes assembled for this study for the presence of *trans*-self chimera transcripts by adopting the strategy of Yang and Smith (2013), and following the procedure described in Davies et al. (2015). All assemblies were queried against the human protein database [containing 23,393 protein sequences, Ensembl 75 (Flicek et al. 2014)] using blastx (version 2.2.29+) with an e-value cut-off of 0.01. High-scoring segment pairs (HSPs) meeting default identification parameters of identity >30% and longer than 100 base-pairs were cut into segments and kept if length was >100 base-pairs. Transcriptome completeness (based on conserved ortholog content) was assessed with BUSCOv.3 (Waterhouse et al. 2017) using HMMERv.3.1b2, blast+v.2.7.1 and AUGUSTUSv.3.2.3 and using the mammalia_odb9 (eukaryota) database.

(2) Protein-coding gene annotation of de novo assemblies

Human protein sequences (Ensembl 75) were filtered by longest sequence per gene and used as tblastn (version 2.2.29+) queries against each of the 15 subterranean mammal databases. Only the top hit, with an e-value cut-off $<1e^{-6}$, was kept. In addition, reciprocal blastx searches were conducted with transcripts used as queries against the human protein

databases with only the top hit retained if e-value <1e^{-6}. Percentage coverage of each hit against the human proteins was calculated with the perl script analyze_blastPlus_topHit_coverage.pl available in Trinity utils. Transcripts meeting the above criteria of reciprocal blasts were kept for further analysis. We visualised blast annotated gene content of the 12 transcriptome datasets, and the gene predictions from the naked mole-rat genome, and the three subterranean groups that contained transcriptome data (golden moles, African mole-rats and spalacids) using UpSet plots (Lex et al. 2014). These candidate CDSs were then filtered by the 9,034 one-to-one orthologous protein coding genes shared between human, guinea pig, elephant, mouse and common shrew, obtained from Ensembl 75.

(3) Mammal-wide alignment construction
Candidate subterranean mammal transcripts were combined with one-to-one canonical coding sequences from 26 species downloaded via the Ensembl-API; all Ensembl sequences with >50% missing data were removed. In total, FASTA files consisted of a maximum of 41 species per CDS. Sequences were screened for incomplete codons at the beginning or end of the sequence; if applicable Ns were added to complete the codon. Sequences containing internal stop codons were removed, as were short sequences <150 nucleotides as these are likely to lead to alignment errors. Sequences were aligned with GUIDANCEv1.41 (Penn et al. 2010) and the PRANK algorithm (Löytynoja and Goldman 2005) with codons enforced and 10 bootstraps. Sequences with a GUIDANCE score <0.6 were removed and the alignment process repeated. Columns scoring <0.93 were removed, as were columns containing >50% gaps using a Perl script. Sequences <150 nucleotides (50 codons) were removed from alignments, and only alignments >100 codons were kept. Alignments were only kept if they contained species from at least two independent origins of subterranean lifestyle – resulting in a dataset of 8,311 nuclear genes (see supplementary fig. S1)

(4) Testing for positive selection associated with the subterranean life
To identify putative genes involved in the adaptive evolution of mammals as they transitioned to a subterranean life-style, we tested each gene for the presence of positive selection along the branch leading to the inferred origin of underground life in our focal clades (fig. 1). For the four groups of interest (mole-rats, golden moles, spalacids and star-nosed mole) alignments were pruned to include a single subterranean clade and its closely related non-subterranean sister-taxa. Alignments consisted of mole-rats plus 1–5 Glires; spalacids plus 1–5 Glires; golden moles plus 1–3 Afrotheria and 1–2 Xenarthra; and the star-nosed mole plus 3–8 Laurasiatheria. Any alignment containing less than four species were removed. In addition, we used these taxonomically reduced sets of alignments to test for positive selection acting on four terrestrial ‘control’ branches (branch E: ancestral branch of elephant + hyrax; branch F: guinea pig; branch G: ancestral branch of mouse + rat and branch H: common shrew, fig. 1). In an attempt to minimise the potentially confounding effects of life-history traits on substitution rates, the closest possible sister taxa (taking into account genome quality, sampling completeness and divergence levels) were chosen as these terrestrial ‘control’ taxa.

We implemented branch-site models (Model A) (Zhang et al. 2005) with codeml in PAML v.4.7. (Yang 2007) to test for positive selection along each of the four branches of interest (fig. 1). Under model A, one branch is designated the foreground branch of interest and site-wise estimates of ω (the ratio of the number of nonsynonymous substitutions per

non-synonymous site to the number of synonymous substitutions per synonymous site) are estimated separately for this focal branch as well as across the remaining background branches in the species tree. The branch-site model estimates four site-classes from the data; (i) constrained ($0<\omega_0<1$), (ii) $\omega_1=1$ (iii) ω_{2a} can exceed 1 on the foreground but is constrained to be under purifying selection on the background, and (iv) ω_{2b} can exceed 1 on the foreground but not on the background. This model is then compared with the null model A, using the likelihood ratio test (LRT), and the significance of the model fit assessed by Chi-squared test with one degree of freedom, with *P*-values <0.05 indicating the alternative model has a significantly better fit compared to the null. The species tree topology follows previous studies (Blanga-Kanfi et al. 2009; Meredith et al. 2011; Davies et al. 2014; Lin et al. 2014; Davies et al. 2015), with species not present in the specific CDS alignment pruned.

To reduce the number of false positives due to alignment errors we adopted an approach that removes genes with highly aggregated positively selected sites [for more details see Davies et al. (2015) and Tsagkogeorga et al. (2015)]. Briefly, the median interval was calculated between sites identified as being under positive selection and an alignment was removed if the median interval was ≤ 10 amino acids. We applied the false discovery rate (FDR) (Benjamini and Hochberg 1995) to the *P*-values calculated above to account for the multiple tests performed with a significant level of 0.05. Overlap in the gene sets under selection in the four groups were visualised as UpSet plots (Lex et al. 2014), and the significance of the intersections, against the background of the genes tested, examined with the supertest function from the SuperExactTest v.0.99.4 package (Wang et al. 2015). We used topGO (Alexa and Rahnenfuhrer 2010) implemented in R v.2.15 (R Development Core Team 2012) to assess enrichment in PSGs. We downloaded GO term accession codes and associated GO domains for each gene tested for selection from Ensembl. This information was then divided into three GO maps based on the three GO domains: (i) molecular function, (ii) cellular component and (iii) biological process. We implemented Fisher's exact test to identify overrepresented gene sets carried out using the classic, elim and weight algorithms, the latter two algorithms take account of GO topology.

(5) Analyses of convergent sequence evolution between divergent subterranean lineages
In order to obtain a measure of total convergent (i.e. parallel and convergent) amino acids substitutions shared between lineages of subterranean mammals, for each locus we characterised the distribution of sequence convergence between pairs of branches in the species phylogeny following the approach described by Castoe et al. (2009) and implemented with the package codeML Ancestral. In this method, posterior probabilities (PP) of all possible amino-acid substitutions were calculated along each branch of the species tree (fig. 1) under the Dayhoff model of amino-acid substitution. These PPs are then used to calculate the probability of all possible convergent and divergent substitutions. For all pair-wise branch comparisons where both branches followed divergent paths, the sum of the joint probabilities of all possible pairs of convergent substitutions (same amino acid) and divergent substitutions were calculated. For each gene alignment analysed, branch lengths and model parameters were first estimated using PAML v.4.7. (Yang 2007).

For each locus, we used the PP of divergent or convergent substitution that corresponded to the most ancestral pair-wise comparison possible given the taxonomic sampling. In an attempt to remove alignments containing errors, which may bias convergence probabilities, we filtered alignments that had scored a median interval

between PSS in any of the four subterranean clades. This resulted in 594 alignments being removed from the dataset. For each of our pair-wise comparisons of interest, we identified all loci returning a summed PP of total convergence of >1.00 (this value was chosen as the lower threshold that can correspond to a maximum of one convergent amino acid site), and then performed GO term enrichment analyses on these genes using topGO, as described above.

We predicted that if convergent molecular evolution had taken place between subterranean lineages, then the greatest levels would occur between taxa known to have independently adopted a subterranean lifestyle, compared to either between terrestrial lineages or between subterranean and terrestrial taxa. Therefore, we compared the number, identity and biological function (based on GO enrichment) of loci identified as sharing convergent substitutions in the six pairs of subterranean with two 'control' comparisons; firstly, pairs of terrestrial taxa, and secondly, a pair consisting of one subterranean and one terrestrial taxon. As mentioned above, these species were chosen as the closest possible sister taxa to the subterranean lineages to minimise the potentially confounding effects of life-history traits on substitution rates while taking into account genome quality, sampling completeness and divergence levels.

As convergent amino acids may arise due to neutral processes, as well as via adaptation, we explored the relationship between branch lengths and the estimated levels of convergent substitutions in our dataset. We estimated branch lengths with RAxML v. 7.2.8 (Stamatakis 2006) set to optimise branch lengths of our constrained species topology using a concatenated amino acid alignment of the 429 loci (236,199 amino acids) containing all 41 taxa under the PROTGAMMADAYHOFF model. We then plotted estimated levels of convergence against the measures of branch length. Finally, using this branch length information, we assigned our second 'control' comparison (terrestrial + subterranean taxa) pairs to be either 'conservative' (long branches) or 'relaxed' (short branches) based on the summed branch lengths of the pairs of taxa concerned.

Supplementary Results

(1) Taxonomic representation and alignment construction

Our BUSCO assessment of the completeness of the transcriptome used in this study suggested that, aside from the *Heliophobius emini* assembly, the assemblies typically contained ~50% complete BUSCOs (supplementary table S2). There was no obvious effect of either tissue source or sequencing technology on the estimates of BUSCO completeness, although given the small sample size it is not possible to test this statistically. Despite tissue source, study and sequencing technology varying across samples, the four spalacid species consistently had the most complete BUSCOs. We further examined protein coding gene representation across the different transcriptome assemblies using UpSet plots (supplementary fig. S2). Unsurprisingly, out of the 23,393 human proteins used as queries, the most were recovered in the gene predictions from the naked mole-rat genome (16,625) and the least in the *H. emini* assembly (9,243). Overlap across the 13 datasets was high – with 5623 common genes. Only 1027 genes were found to be unique to the naked mole-rat, which suggests that the tissue source did not have a large effect on the genic content. When the 13 transcriptome datasets are collapsed into three families (golden moles, African mole-rats and spalacids), the number of genes represented across all three groups is 11,719 (supplementary fig. S2B).

(2) Testing for positive selection in subterranean and non-subterranean linages

(a) Genes under positive selection

To test for site-specific positive selection in each of the four clades of subterranean mammals we ran branch-site models (model MA) for ~8,000 genes. We first explored the taxonomic coverage in our test datasets and found that ~50% had all taxa present across the four groups (supplementary fig. S3). Tests performed on the ancestral branch of each clade (represented by a single species of star-nosed mole, *Condylura cristata*, in talpid moles) revealed a total of 1419 cases of positive selection, encompassing 1267 different positively selected genes (PSGs). Numbers of PSGs per focal branch ranged from 279 in the Spalacidae to 483 in the star-nosed mole (Table 1, supplementary tables S3–S6). We confirmed that the non-subterranean sister taxon was also present in the majority of these cases. Specifically, tenrec was present in 83% of PSGs (295/356) found in golden moles, guinea pig in 99% (297/301) of PSGs in African mole-rats, mouse and/or rat in 99.6% (278/279) of PSGs in spalacids, and shrew in 94% (452/483) of PSGs in star-nosed mole. After adjusting for the false discovery rate (FDR), the number of PSGs ranged from six in the golden moles to 37 in the star-nosed mole (Table 1). These numbers are comparable to the number of PSGs found in the four control taxa, where a total of 2270 genes under positive selection were found, with 1946 different genes under selection across all four non-subterranean control taxa. Number of PSGs per control branch ranged from 303 in the ancestral elephant+hyrax branch, to 742 and 763 in the guinea pig and common shrew, respectively, with the two lineages each represented by a single species (Table 1, supplementary tables S7–S10). We compared genes found to be under selection in each of the focal subterranean lineages with those under selection in their respective control group (supplementary fig. S4A). Across all four comparisons less than 5% of the PSGs were found to be under section in both branches (supplementary table S11). Significance tests, using the supertest function (Wang et al. 2015), of these gene set intersections revealed significant intersections between overlapping PSGs in two comparisons: golden moles and Elephant+hyrax (fold enrichment = 1.48, p = 0.045), and spalacids and mouse+rat (fold enrichment = 1.81, $p = 9.04 \times 10^{-4}$) (see supplementary Table S12). Comparing the pooled 1267 genes under selection in subterranean taxa with the 1946 PSGs in non-subterranean species revealed an overlap of 434 genes (13.5% of the total PSGs) (supplementary fig. S4B). When only PSGs meeting the FDR threshold < 0.05 are considered, an overlap of five genes is found between the 71 PSGs in subterranean species and 271 PSGs in non-subterranean species.

To determine the corresponding extent of parallel molecular adaptation among the four control non-subterranean groups, we compared their respective sets of PSGs and found that 287 loci were under selection in at least two groups – with six of these being robust to FDR adjustment. Of these 287 loci, four (*CDH17*, *P2RX7*, *PKD1L1* and *XIRP2*) showed positive selection in all four groups, and 29 loci showed positive selection in three of the groups (supplementary fig. S4C). Significance tests, using the supertest function (Wang et al. 2015), of these gene set intersections (against 6148 background genes) revealed significant intersections between overlapping PSGs in all comparisons other than Guinea pig and common shrew, Elephant+hyrax and mouse+rat, Elephant+hyrax and common shrew, and Elephant+hyrax and Guinea pig (see supplementary Table S12B).

We detected positive selection in a number of genes that may relate to molecular adaptations in subterranean mammals, however, positive selection was also detected in non-subterranean ‘control’ taxa and therefore, they cannot be said to exclusively relate to

subterranean adaptations. For example, six of the 17 genes previously linked to Fanconi anaemia (and therefore, also a response to DNA damage) were found to be positively selected: *BRCA2*–golden moles and the star-nosed mole, *FANCB*–golden moles, *FANCC*–African mole-rats, *FANCE* and *FANCI*–the star-nosed mole, and *SLX4*–spalacids. In comparison eight of these genes were found to be under positive selection in non-subterranean species: *BRCA1* and *FANCG*–common shrew, *BRCA2* and *FANCB*–both common shrew and mouse+rat, *FANCE*–guinea pig and *BRIP1*, *FANCM* and *PALB2*–mouse+rat.

We found at least five myosins with known roles in hearing to be under positive selection across subterranean lineages. These loci, with the respective groups in which they show selection were *MYO1A* (*DFNA48*) in star-nosed moles, *MYO3A* (*DFNB30*) in African mole-rats, *MYH14* (*DFNA4*) in golden moles and the star-nosed mole, *MYO15A* (*DFNB3*) in golden moles, and *MYO9B*, in African mole-rats and golden moles. Across the subterranean taxa we found positive selection in 10 myosin-related genes; aside from the five 'sensory' myosins mentioned above, the five genes were associated with either skeletal or cardiac muscle contraction (*MYBPC1, MYBPC3, MYBPHL, MYL6B* and *MYOM3*). However, positive selection was also detected in a number of myosins in the non-subterranean lineages; for example, *MYO1A*, *MYO9B* and *MYOM3* in the elephant+hyrax, *MYOT*, *MYO15A* and *MYO9A* in the guinea pig, *MYO9B*, *MYO1A*, *MYOM3*, *MYOM1* and *MYO1C* in the mouse+rat, and *MYO7B*, *MYOM3* and *MYO15A* in the common shrew. The gene *CFDP1* (*Craniofacial Development Protein 1*) was found to be under selection in the star-nosed mole, and also in the guinea pig. Positive selection in the subterranean lineages was detected in many genes encoding components of the extracellular matrix, including collagen-related (*COL12A1*, *COL14A1*, *COL1A2*, *COL3A1*, *COL4A5*, *COL5A2*, *COLGALT1*), elastin-related (*EMILIN1* and *ELANE*) and laminin-related (*LAMA3*, *LAMA4* and *LAMB3*) genes (supplementary tables S3–10). However, positive selection was also detected in multiple collagens and laminins in control taxa; e.g., *COL6A6*, *COL4A5*, *COL27A1* and *LAMB3* along the elephant+hyrax branch. Along the golden mole branch, positive selection was also found in three genes associated with the glycosaminoglycan hyaluronan (*HABP2*, *HAS3* and *HMMR*) compared to positive selection in a single hyaluronan related gene, *HYAL4* and *HMMR*, along the elephant+hyrax and mouse+rat branch, respectively.

(b) GO functional enrichment of positively selected genes

For each set of genes identified as being under positive selection in either each of the four subterranean and non-subterranean lineages, we performed tests of gene ontology (GO) enrichment across three domains [molecular function (MF), cellular component (CC) and biological process (BP)]. Numbers of enriched GO terms in the control taxa also broadly reflected those of PSGs in each clade, with the Elephant+hyrax having the fewest enriched GO terms across domains and the guinea pig typically the most (Table 1 and supplementary tables S18–21). As in the subterranean lineages, we found limited evidence of overlap between enriched BP GO terms in the non-subterranean lineages (supplementary fig. S5–6), with the single term ('Response to cytokine') enriched in all four lineages. The following terms: 'Cell activation', 'Protein activation cascade', 'Cell adhesion', 'Biological adhesion', 'Cellular response to cytokine stimulus' and 'Fibrinolysis' were enriched across three non-subterranean lineages and are associated with an immune response. Perhaps surprisingly, relatively few significantly enriched terms relating to

sensory perception were found in the four non-subterranean taxa. With two such enriched BP terms in the common shrew (‘Sensory perception of pain’ and ‘Detection of mechanical stimulus’), three terms relating to external stimulus in the elephant+hyrax branch (‘Response to external stimulus’, ‘Regulation of response to external stimulus’, ‘Positive regulation of response to external stimulus’), and eight terms in guinea pig (‘Detection of mechanical stimulus’, ‘Response to mechanical stimulus’, ‘Detection of mechanical stimulus involved in sensory perception’, ‘Positive regulation of response to external stimulus’, ‘Detection of external stimulus’, ‘Detection of abiotic stimulus’, ‘Response to external stimulus’, ‘Positive regulation of response to stimulus’ (supplementary table S18–21).

(3) Analyses of convergent sequence evolution between divergent subterranean lineages
We estimated the branch lengths across the taxa included in our study by optimising branch lengths using a maximum likelihood analysis of a concatenated amino acid alignment of 429 loci (236,199 amino acids) containing all 41 taxa and the constrained species topology (supplementary fig. 7A). From this tree it is evident that there is considerable variation in branch lengths across the sample, with taxa such as the common shrew and star-nosed mole having much longer branches than for example, the African mole-rats. We found that across all groups, there was a significant positive relationship between the number of genes showing evidence of convergent substitutions and summed branch length (Number of ‘convergent’ genes = 27002 x Branch length – 398, $R^2 = 0.709$, $F = 53.66$ (df 1, 22), $P = 2.47 \times 10^{-7}$). Based on this relationship, the points corresponding to the subterranean pairs fall as expected and therefore, do not appear to show higher levels of convergence compared to other control comparisons given the branch lengths (supplementary fig. 7B, and supplementary fig. S8).

To compare levels of enriched BP terms, broadly relating to blood/oxygen, sensory perception and immune response, across subterranean and non-subterranean pairs we grouped functionally related terms based on key words and examined the frequency distribution of numbers of pairs with enriched terms (supplementary fig. S9). Across all three categories (Blood, Sensory and Immunity), and in both subterranean and non-subterranean pairs, the bin corresponding to a single pair has the highest frequency – suggesting that many convergent amino acids do not occur in functionally identical genes. Generally, across all three categories, and in particular Immunity, bins with higher numbers of pairs were found in the subterranean taxa. Although given the hierarchical nature of GO terms the significance of this is difficult to assess.

Supplementary Tables

Table S1
Taxa and datasets included in the current study.

Order	Family	Species (common name)	Assembly/SRA ID (ref.)
Diprotodontia	Macropodidae	*Macropus eugenii* (Wallaby)	Meug_1.0
Didelphimorphia	Didelphidae	*Monodelphis domestica* (Gray short-tailed opossum)	monDom5
Dasyuromorphia	Dasyuridae	*Sarcophilus harrisii* (Tasmanian devil)	Devil_ref v7.0
Afrosoricida	Chrysochloridae	*Amblysomus hottentotus* (Hottentot golden mole)	SRP126619[a]
		Chrysochloris asiatica (Cape golden mole)	ChrAsi1.0
	Tenrecidae	*Echinops telfairi* (Lesser hedgehog tenrec)	TENREC
Hyracoidea	Procaviidae	*Procavia capensis* (Hyrax)	proCap1
Pilosa	Megalonychidae	*Choloepus hoffmanni* (Sloth)	choHof1
Cingulata	Dasypodidae	*Dasypus novemcinctus* (Armadillo)	Dasnov3.0
Proboscidea	Elephantidae	*Loxodonta Africana* (Elephant)	loxAfr3
Rodentia	Bathyergidae	*Bathyergus suillus* (Cape dune mole-rat)	SRR2141210[a] (Davies et al. 2015)
		Cryptomys hottentotus mahali (Lesotho mole-rat)	SRR2141211[a] (Davies et al. 2015)
		Cryptomys h. natalensis (Natal mole-rat)	SRR2141212[a] (Davies et al. 2015)
		Cryptomys h. pretoriae (Highveld mole-rat)	SRR2141213[a] (Davies et al. 2015)
		Fukomys damarensis (Damaraland mole-rat)	SRR2141214[a] (Davies et al. 2015)
		Georychus capensis (Cape mole-rat)	SRR2141216[a] (Davies et al. 2015)
		Heliophobius emini (Silvery mole-rat)	SRR2141215[a] (Davies et al. 2015)
		Heterocephalus glaber (Naked mole rat)	HetGla_female_1.0 (Keane et al. 2014)
	Caviidae	*Cavia porcellus* (Guinea pig)	cavPor3
	Muridae	*Mus musculus* (Mouse)	GRCm38
		Rattus norvegicus (Rat)	Rnor_5.0
	Spalacidae	*Tachyoryctes splendens* (East African root rat)	SRR2141217[a] (Davies et al. 2015)
		Nannospalax galili	[a](Malik et al. 2011)

		(Northern Israeli blind subterranean mole rat)	
		Rhizomys pruinosus (Hoary bamboo rat)	PRJNA211727[a] (Lin et al. 2014)
		Eospalax baileyi (Plateau zokor)	PRJNA208780[a] (Lin et al. 2014)
	Sciuridae	*Ictidomys tridecemlineatus* (Thirteen-lined ground squirrel)	spetri2
Lagomorpha	Leporidae	*Oryctolagus cuniculus* (Rabbit)	OryCun2.0
Primates	Hominidae	*Homo sapiens* (Human)	GRCh37
		Pan troglodytes (Chimpanzee)	CHIMP2.1.4
		Pongo abelii (Orangutan)	PPYG2
	Hylobatidae	*Nomascus leucogenys* (Gibbon)	Nleu1.0
	Galagidae	*Otolemur garnettii* (Bushbaby)	OtoGar3
Soricomorpha	Talpidae	*Condylura cristata* (Star-nosed mole)	ConCri1.0
	Soricidae	*Sorex araneus* (Common shrew)	COMMON_SHREW1
Erinaceomorpha	Erinaceidae	*Erinaceus europaeus* (Hedgehog)	HEDGEHOG
Perissodactyla	Equidae	*Equus caballus* (Horse)	EquCab2
Cetartiodactyla	Camelidae	*Vicugna pacos* (Alpaca)	vicPac1
	Delphinidae	*Tursiops truncatus* (Bottle nosed dolphin)	turTru1
Chiroptera	Vespertilionidae	*Myotis lucifugus* (Little brown bat)	Myoluc2.0
Carnivora	Felidae	*Felis catus* (Cat)	Felis_catus_6.2
	Canidae	*Canis lupus familiaris* (Dog)	CanFam3.1

[a] transcriptome dataset.
Subterranean taxa are underlined.

Table S2
Source, tissue and statistics for the subterranean mammal transcriptome datasets.

**Species** (source)	**Tissue**	**Reads**	**No. transcripts** (average length)	**No. cut transcripts** (average length)	**BUSCO %** (*n*=4104)
Amblysomus hottentotus (This study)	Brain, liver, lung, skeletal muscle, heart and kidney	17,358,151	145,570 (630)	146,291 (621)	C:52.7 [S:37.4, D:15.3], F:30.2, M:17.1
Bathyergus suillus (Davies et al. 2015)	Brain, liver, lung, skeletal muscle, heart and kidney	18,175,296	176,294 (614)	177,332 (604)	C:54.0 [S:38.0, D:16.0], F:27.9, M:18.1
Cryptomys h. mahali (Davies et al. 2015)	Brain, liver, lung, skeletal muscle, heart and kidney	17,253,040	318,549 (574)	320,282 (564)	C:48.0 [S:31.8, D:16.2], F:30.0, M:22.0
Cryptomys h. natalensis (Davies et al. 2015)	Brain, liver, lung, skeletal muscle, heart and kidney	17,290,951	259,886 (494)	260,393 (488)	C:33.1 [S:20.9, D:12.2], F:42.7, M:24.2
Cryptomys h. pretoriae (Davies et al. 2015)	Brain, liver, lung, skeletal muscle, heart and kidney	17,833,375	226,694 (567)	227,740 (559)	C:50.6 [S:34.9, D:15.7], F:31.2, M:18.2
Fukomys damarensis (Davies et al. 2015)	Brain, liver, lung, skeletal muscle, heart and kidney	18,220,998	244,256 (602)	245,682 (589)	C:54.2 [S:35.0, D:19.2], F:28.6, M:17.2
Georychus capensis (Davies et al. 2015)	Brain, liver, lung, skeletal muscle, heart and kidney	20,038,942	228,786 (416)	229,151 (414)	C:32.1 [S:25.6, D:6.5], F:32.6, M:35.3
Heliophobius emini (Davies et al. 2015)	Brain, liver, lung, skeletal muscle, heart and kidney	17,410,109	105,595 (367)	105,685 (366)	C:10.4 [S:8.2, D:2.2], F:29.4, M:60.2
Tachyoryctes splendens (Davies et al. 2015)	Brain, liver, lung, skeletal muscle, heart and kidney	18,135,605	237,399 (630)	238,843 (619)	C:62.2 [S:40.8, D:21.4], F:25.6, M:12.2
Rhizomys pruinosus (Lin et al. 2014)	Brain and liver	140,777,831	201,452 (1055)	204,391 (1011)	C:84.7 [S:45.6, D:39.1], F:8.8, M:6.5
Eospalax baileyi (Lin et al. 2014)	Brain and liver	50,318,570	332,182 (695)	334,879 (678)	C:72.5 [S:43.7, D:28.8], F:16.3, M:11.2
Nannospalax galili (Malik et al. 2011)	Brain and skeletal muscle	NA	51,439	NA	C:58.2 [S:41.2, D:17.0], F:19.2, M:22.6

Abbreviations: n – total BUSCO groups searched; C – complete BUSCOs; S – complete and single-copy BUSCOs; D – complete and duplicated BUSCOs; F – fragmented BUSCOs; M – missing BUSCOs

Supplementary tables S3-S10 as separate file: Supplementary_tables_S3_10_PAML.xlsx

Supplementary table S3

Results of branch-site model tests for positive selection based on the golden moles. Genes with median interval PSS ≤ 10 (likely reflecting false positives due to alignment errors) and alignments with <4 taxa (shaded in grey) were removed from the dataset. Abbreviations: lnL – log-likelihood; df – degrees of freedom; LRT – likelihood ration test; FDR – false discovery rate; P – proportion of sites; ω – omega/dN/dS; PSS – positively selected sites. * indicates inaccurate ω estimates likely due to no synonymous sites.

Supplementary table S4

Results of branch-site model tests for positive selection based on the African mole-rats. Genes with median interval PSS ≤ 10 (likely reflecting false positives due to alignment errors) and alignments with <4 taxa (shaded in grey) were removed from the dataset. Abbreviations: lnL – log-likelihood; df – degrees of freedom; LRT – likelihood ration test; FDR – false discovery rate; P – proportion of sites; ω – omega/dN/dS; PSS – positively selected sites. * indicates inaccurate ω estimates likely due to no synonymous sites.

Supplementary table S5

Results of branch-site model tests for positive selection based on the spalacids. Genes with median interval PSS ≤ 10 (likely reflecting false positives due to alignment errors) and alignments with <4 taxa (shaded in grey) were removed from the dataset. Abbreviations: lnL – log-likelihood; df – degrees of freedom; LRT – likelihood ration test; FDR – false discovery rate; P – proportion of sites; ω – omega/dN/dS; PSS – positively selected sites. * indicates inaccurate ω estimates likely due to no synonymous sites.

Supplementary table S6

Results of branch-site model tests for positive selection based on the star-nosed mole. Genes with median interval PSS ≤ 10 (likely reflecting false positives due to alignment errors) and alignments with <4 taxa (shaded in grey) were removed from the dataset. Abbreviations: lnL – log-likelihood; df – degrees of freedom; LRT – likelihood ration test; FDR – false discovery rate; P – proportion of sites; ω – omega/dN/dS; PSS – positively selected sites. * indicates inaccurate ω estimates likely due to no synonymous sites.

Supplementary table S7

Results of branch-site model tests for positive selection based on the elephant+hyrax branch. Abbreviations: lnL – log-likelihood; df – degrees of freedom; LRT – likelihood ration test; FDR – false discovery rate.

Supplementary table S8

Results of branch-site model tests for positive selection based on the guinea pigs. Abbreviations: lnL – log-likelihood; df – degrees of freedom; LRT – likelihood ration test; FDR – false discovery rate.

Supplementary table S9

Results of branch-site model tests for positive selection based on the mouse+rat branch. Abbreviations: lnL – log-likelihood; df – degrees of freedom; LRT – likelihood ration test; FDR – false discovery rate.

Supplementary table S10

Results of branch-site model tests for positive selection based on the common shrew. Abbreviations: lnL – log-likelihood; df – degrees of freedom; LRT – likelihood ration test; FDR – false discovery rate.

Table S11

(A) The seven genes identified as being under positive selection in three subterranean lineages.

**Gene:**	**Function/role:**	**Positive selection**			
		**GM**	**AMR**	**SP**	**SNM**
C3	Activation of complement system	Yes	Yes	Yes	–
ADGRE5	Cell adhesion as well as leukocyte recruitment, activation and migration	–	Yes	Yes	Yes
GLS2	Regulation of glutamine catabolism	Yes	Yes	–	Yes
LCT	Splits lactose	Yes	Yes	–	Yes
SLC29A3	Cellular uptake of nucleosides, nucleobases	Yes	Yes	–	Yes
SVIL	Forms a high-affinity link between the actin cytoskeleton and the membrane	Yes	Yes	–	Yes
TEX15	Unclear	–	Yes	Yes	Yes

Abbreviations: GM – golden moles; AMR – African mole-rats, SP – spalacids, SNM – star-nosed mole.

(B) Number and identity of common genes found to be under significant positive selection in both a subterranean lineage and its corresponding non-subterranean control lineage.

(Branch) subterranean #PS genes	(Branch) control #PS genes	#genes shared (% of PSGs)	Abbreviated gene name
(A) Golden moles 356	(E) Elephant+hyrax 303	21 (3.19)	*ADGRG7, ATP11C, CD109, CDH17, CSN3, FBXW9, GP5, LMF1, MGAM, MTIF2, MYH14, MYO9B, P2RX7, POLA2, POLR2B, PRIM2, PTCH1, RBM34, SLC39A4, TGFB1, VAV1*
(B) African mole-rats 301	(F) Guinea pig 742	40 (3.84)	*ABAT, ADAM18, ADGRF3, C3, CD274, CEACAM16, CROCC, DNMT1, ESAM, FAM208B, FZD6, GFPT2, ICE1, IRF3, ITGA1, ITGB5, JAG1, LCA5, MAP3K13, NAALADL1, NAGLU, NLRX1, NTN5, OASL, PAK5, PKD1L1, PPP1R9A, PRSS54, RAB3GAP2, RNF43, SLC15A2, SNTB1, TANGO6, TEX15, TMIGD1, TTC9, UIMC1, VPS13B, VSIG10L*
(C) Spalacids 279	(G) Mouse+rat 462	31 (4.18)	*A2M, ARHGAP36, CDH17, CTNND1, DUOX1, ESCO1, EXPH5, FSTL4, HAUS6, HSF2BP, IGSF11, IL12RB2, ITGAL, KNTC1, LACTB2, LMF1, MMP9, MYBPC1, PATJ, PPIP5K2, RET, SDE2, SEC24D, SGO1, SHROOM1, SLC6A19, STAB1, TEX15, THNSL2, TLR4, TM4SF20*
(D) Star-nosed mole 483	(H) Common shrew 763	62 (4.98)	*ADAMTS12, ADGRG7, ALDH18A1, ANPEP, APAF1, AQR, ARHGEF17, AZIN2, BRCA2, C19orf45, CAPN11, CCDC175, CCDC36, CFI, CHAC1, COMT, CTSW, DDIAS, DLEC1, DLGAP5, ENTPD1, EPB41L5, GC, GLB1, GLMP, HERC6, HTT, IKBKAP, ITGAE, KCNC2, LAPTM5, LCT, LGMN, LRIG1, MCM8, MEP1B, MORC3, MYOM3, NEU3, NIN, NME8, P2RX7, PI4KA, PITPNM3, RAG1, SERPINE1, SH3TC2, SHROOM1, SLAMF1, SMC1B, TBC1D4, TCHHL1, TEX15, TKFC, TLR2, TM7SF2, TRIP11, UBA7, USP26, USPL1, VSIG10L, XAF1*

Table S12

(A) Test of the significance of positively selected gene intersections between the four subterranean groups using the supertest function from the SuperExactTest package (Wang et al. 2015).

Test	Intersections	Obs. overlap	Exp. overlap	FE	*P*-value
1	SNM & SP	22	20.62	1.07	0.407
1	AMR & SP	22	12.85	1.71	0.009
1	AMR & SNM	27	22.24	1.21	0.168
1	AMR & SNM & SP	2	0.95	2.11	0.246
1	GM & SP	16	15.20	1.05	0.453
1	GM & SNM	34	26.31	1.29	0.071
1	GM & SNM & SP	0	1.12	0	1
1	GM & AMR	38	16.39	2.32	$7.53x10^{-7}$
1	GM & AMR & SP	1	0.70	1.43	0.504
1	GM & AMR & SNM	4	1.21	3.30	0.034
1	GM & AMR & SNM & SP	0	0.05	0	1
2	AMR & SP	22	11.22	1.96	0.002
3	AMR & GM	38	13.96	2.72	$1.23x10^{-8}$
4	AMR & SNM	27	20.80	1.30	0.096
4	GM & SNM	34	24.61	1.38	0.032
4	GM & AMR	38	15.33	2.48	0.000
4	GM & AMR & SNM	4	1.06	3.77	0.022

Note – The background number of genes for each test carried out were as follows: Test 1 = 6536 genes present and tested in all four groups; Test 2 = 7485 genes present and tested in the African mole-rats and spalacids; Test 3 = 7675 genes present and tested in African mole-rats and golden moles; Test 4 = 6988 genes present and tested in the star nosed-mole, golden moles and African mole-rats.

Abbreviations: FE – fold enrichment; Obs. – observed; Exp. – expected; SP – spalacids; SNM – star nosed-mole; AMR – African mole-rats; GM – golden moles

(B) Test of the significance of positively selected gene intersections between the four non-subterranean groups using the supertest function from the SuperExactTest package (Wang et al. 2015).

Intersections	Obs. overlap	Exp. overlap	FE	*P*-value
CS & M+R	72	57.34	1.26	0.021
GP & M+R	75	55.76	1.35	0.004
GP & CS	104	92.09	1.13	0.089
GP & CS & M+R	19	6.92	2.75	$8.12x10^{-5}$
E+H & M+R	29	22.77	1.27	0.103
E+H & CS	44	37.60	1.17	0.146
E+H & CS & M+R	9	2.83	3.18	0.002
E+H & GP	41	36.57	1.12	0.235
E+H & GP & M+R	8	2.75	2.91	0.007
E+H & GP & CS	9	4.54	1.98	0.040
E+H & GP & CS & M+R	4	0.34	11.73	$4.14x10^{-4}$

Note – The background number of genes for each test carried out were as follows: Test 1 = 6148 genes present and tested in all four groups.

Abbreviations: FE – fold enrichment; Obs. – observed; Exp. – expected; CS – Common shrew; M+R – Mouse + rat; GP – Guinea pig; E+H – Elephant + hyrax

(C) Test of the significance of positively selected gene intersections between the four pair of subterranean and non-subterranean groups using the supertest function from the SuperExactTest package (Wang et al. 2015).

Test	Intersections	Obs. overlap	Exp. overlap	FE	*P*-value
1	GM & E+H	21	14.15	1.48	0.045
2	AMR & GP	40	30.96	1.29	0.053
3	SNM & CS	62	55.24	1.12	0.176
4	SP & M+R	31	17.15	1.81	9.04×10^{-4}

Note – The background number of genes for each test was calculated as the common genes tested in both lineages and are as follows: Test 1 = 7621; Test 2 = 7214; Test 3 = 6671; Test 4 = 7517 genes.

Abbreviations: FE – fold enrichment; Obs. – observed; Exp. – expected; CS – Common shrew; M+R – Mouse + rat; GP – Guinea pig; E+H – Elephant + hyrax

Supplementary tables S13-16 as separate file:
Supplementary_tables_S13_16_TopGo_PAML.xlsx

Supplementary table S13
GO enrichment based on PSGs in golden moles.
(A) Molecular Function GO terms; (B) 'Cellular Component' terms; (C) 'Biological Process' GO terms; enriched by at least one of the three tests (classic, elim or weight). Abbreviations: fis – Fisher's exact test. Significant terms ($P < 0.05$) are shown by bold font.

Supplementary table S14
GO enrichment based on PSGs in African mole-rats.
(A) Molecular Function GO terms; (B) 'Cellular Component' terms; (C) 'Biological Process' GO terms; enriched by at least one of the three tests (classic, elim or weight). Abbreviations: fis – Fisher's exact test. Significant terms ($P < 0.05$) are shown by bold font.

Supplementary table S15
GO enrichment based on PSGs in spalacids.
(A) Molecular Function GO terms; (B) 'Cellular Component' terms; (C) 'Biological Process' GO terms; enriched by at least one of the three tests (classic, elim or weight). Abbreviations: fis – Fisher's exact test. Significant terms ($P < 0.05$) are shown by bold font.

Supplementary table S16
GO enrichment based on PSGs in the star-nosed mole.
(A) Molecular Function GO terms; (B) 'Cellular Component' terms; (C) 'Biological Process' GO terms; enriched by at least one of the three tests (classic, elim or weight). Abbreviations: fis – Fisher's exact test. Significant terms ($P < 0.05$) are shown by bold font.

Table S17

Grouped 'Biological Process' (BP) GO terms found to be significantly enriched based on PSGs in the four subterranean lineages tested using Fisher's exact test and the classic algorithm.

(A) Enriched BP terms based in Golden moles

Category [#terms]	Representative GO Terms	Associated genes
Immune response [7]	e.g. 'Defense response to bacterium', 'Inflammatory response' and 'Response to stress'	82 (e.g. *ALB*, *MAP3K13* and *UVSSA*)
DNA and cell cycle [22]	e.g. 'Nucleotide-excision repair', 'DNA repair' and 'Mitotic cell cycle'	66 (e.g. *CENPM*, *POLA2* and *TOP2A*)
Digestion, catabolic and metabolic process [16]	e.g. 'Digestion', 'Small molecule metabolic process' and 'Hormone metabolic process'	58 (e.g. *HMMR*, *CRK*, and *VCAN*)
Phosphorylation and protein processing [9]	e.g. 'Positive regulation of phosphorylation', 'Protein maturation' and 'Protein processing'	45 (e.g. *IGF1R*, *SERPINB12* and *SLC11A1*)
Ions and transport [15]	e.g. 'Divalent inorganic cation homeostasis', 'Lipid transport' and 'Carbohydrate derivative transport'	42 (e.g. *HEXA*, *SLC7A7* and *SLC9C1*)
Biological adhesion [1]	'Biological adhesion'	30 (e.g. *CDH16*, *COL12A1* and *VEZTI*)
Cell homeostasis and genesis [4]	'Anatomical structure homeostasis', 'Homeostasis of number of cells within a tissue' and 'Gliogenesis'	19 (e.g. *BBS10*, *P2RX7* and *VCAN*)
Positive regulation of transferase activity [1]	'Positive regulation of transferase activity'	18 (e.g. *CXXC1*, *LTF* and *MAP3K10*)
Kinase activity [3]	'Positive regulation of kinase activity', 'Activation of protein kinase activity' and 'Positive regulation of protein kinase activity'	18 (e.g. *SEC31A* , *SLC11A1* and *TOM1L1*)
Sex determination [4]	e.g. 'Sex determination', 'Mammary gland development' and 'Embryonic placenta morphogenesis'	16 (e.g. *BRCA2*, *PTCH1* and *TGFB1*)
Blood [4]	e.g. 'Vascular endothelial growth factor production', 'Positive regulation vascular endothelial growth factor production' and 'Platelet activation'	12 (e.g. *ALB*, *C3*, *PSAP* and *VAV1*)
Inositol mediated signalling [2]	'Phosphatidylinositol-mediated signalling' and 'Inositol lipid-mediated signalling'	11 (e.g. *PIK3C2B*, *PIK3C2G* and *VAV1*)

‘Hyaluronan and collagen’ [7]	e.g. ‘Hyaluronan metabolic process’, ‘Collagen metabolic process’ and ‘Collagen biosynthetic process’	10 (e.g. *COL12A1*, *HAS3* and *HMMR*)
Oxygen [4]	e.g. ‘Regulation of reactive oxygen species metabolic process’, ‘Superoxide anion generation’ and ‘Reactive oxygen species metabolic process’	6 (e.g. *EGFR*, *IL18* and *TGFB1*)
Mineralization [3]	‘Positive regulation of bone mineralization’, ‘Positive regulation of biomineral tissue development’, ‘Positive regulation of ossification’	4 (e.g. *KL*, *P2RX7* and *TGFB1*)
Telomere [3]	‘Telomere maintenance via semi-conservative replication’, ‘Telomere maintenance via recombination’ and ‘Telomere maintenance via telomere lengthening’	4 (e.g. *DNA2*, *POLA2* and *RFC1*)
Sleep [1]	‘Sleep’	4 (e.g. *ALB*, *PTGDR* and *SLC29A1*)
Dermatan/chondroitin sulfate [5]	e.g. ‘Dermatan sulfate metabolic process’, ‘Dermatan sulfate proteoglycan biosynthetic process’ and ‘Chondroitin sulfate biosynthetic process’	*CSPG4*, *DSE*, *VCAN*
Syncytium formation [2]	‘Syncytium formation by plasma membrane fusion’ and ‘Syncytium formation’	*DCSTAMP*, *GCM1* and *NPHS1*
7-methylguanosine mRNA capping [1]	‘7-methylguanosine mRNA capping’	*ERCC3*, *POLR2B* and *RNMT*

(B) Enriched BP terms based in African mole-rats

**Category [#terms]**	**Representative GO Terms**	**Associated genes**
Immune response [56]	e.g. ‘Immune system process’, ‘Defense response to virus’ and ‘Response to other organism’	57 (e.g. *C3*, *ELP2* and *ITGA1*)
Reproduction [6]	e.g. ‘Reproduction’, ‘Mating’, and ‘Spermatogenesis’	42 (e.g. *ABAT*, *FSHR* and *OASL*)
Extracellular [5]	e.g. ‘Biological adhesion’, ‘Extracellular structure organization’ and ‘Integrin-mediated signaling pathway’	35 (e.g. *ADAM28*, *ITGA1* and *LAMA3*)
Organic acid [14]	e.g. ‘Organic acid metabolic process’, ‘Fatty acid metabolic process’ and ‘Aspartate family amino acid catabolic process’	32 (e.g. *GLS2*, *OLAH* and *RARS*)
Cellular ketone metabolic process [1]	Cellular ketone metabolic process	30 (e.g. *ALDH1A2*, *DLST* and *NR5A2*)
Lipid [10]	e.g. ‘Lipid catabolic process’, ‘Cellular lipid catabolic process’ and ‘Lipid homeostasis’	23 (e.g. *ACOX1*, *LIPK* and *NPC1L1)*
Oxidation-reduction process [1]	‘Oxidation-reduction process’	23 (e.g. *COX4I2*, *UQCRC1* and *VAT1L*)
Small molecule process [2]	‘Small molecule catabolic process’ and ‘Small molecule biosynthetic process’	22 (e.g. *ALDH8A1*, *OLAH* and *SCAP*)
Organic transport [2]	‘Organic alcohol transport’ and ‘Organic substance transport’	21 (e.g. *CHRNA3*, *SLC35D3* and *TNFSF11*)
–noid metabolic process [5]	e.g. ‘Diterpenoid metabolic process’, ‘Retinoid metabolic process’ and ‘Steroid metabolic process’	14 (e.g. *APOA4*, *FGF23*, and *TTR*)
Hormone [2]	‘Hormone metabolic process’ and ‘Regulation of hormone levels’	13 (*ALDH1A2*, *CPQ* and *TTR*)
Cholesterol/Sterol [6]	e.g. ‘Cholesterol homeostasis’, ‘Sterol homeostasis’ and ‘Cholesterol metabolic process’	9 (e.g. *APLP2*, *LSS* and *SCAP*)
Vitamin biosynthetic process [4]	e.g. ‘Vitamin biosynthetic process’, ‘Fat-soluble vitamin metabolic process’ and ‘Vitamin metabolic process’	8 (e.g. *ALDH1A2*, *MMAB*, and *PCCB*)
Locomotory behaviour [1]	‘Locomotory behaviour’	8 (e.g. *CHRNA3*, *NAGLU* and *NTAN1*)
Nitrogen compound transport [1]	‘Nitrogen compound transport’	8 (e.g. *ACACB*, *AQP7*, and *SLC17A3*)

Amine transport [2]	'Amine transport' and 'Regulation of amine transport'	6 (e.g. *CHRNA6*, *CPT1A* and *PARK7*)
Regulation of protein binding [1]	'Regulation of protein binding'	5 (e.g. *APLP2*, *TEX14* and *TICAM1*)
Acetyl-CoA metabolic process [1]	'Acetyl-CoA metabolic process'	4 (e.g. *DLST*, *MDH1* and *PIPOX*)
Protein activation cascade [1]	'Protein activation cascade'	4 (e.g. *C5*, *F11* and *GP5*)
Response to dsRNA [2]	'Response to dsRNA' and 'Cellular response to dsRNA'	4 (e.g. *IRF3*, *MAVS* and *TMEM173*)
Dopamine [2]	'Dopamine transport' and 'Catecholamine transport'	*C5*, *CHRNA6* and *PARK7*
Bone [2]	'Regulation of bone resorption' and 'Regulation of bone remodelling'	*FSHR*, *TFRC* and *TNFSF*
Blood [2]	'Negative regulation of blood coagulation' and 'Negative regulation of hemostasis'	*FAP*, *F11* and *GP5*
Cellular amide metabolic process [1]	'Cellular amide metabolic process'	*ACACB*, *MME* and *PCCB*
Positive regulation of transcription factor import into nucleus [1]	'Positive regulation of transcription factor import into nucleus'	*EDAR*, *MAVS* and *TMEM173*
Peroxisome organization [1]	'Peroxisome organization'	*ACOX1*, *MAVS* and *PEX16*

(C) Enriched BP terms based in Spalacids

Category [#terms]	Representative GO Terms	Associated genes
Immune response [34]	e.g. 'Regulation of innate immune response', 'Positive regulation of leukocyte mediated immunity' and 'Defense response to bacterium'	32 (e.g. *A2M*, *ITGAL* and *ST3GAL6*)
Homeostasis [10]	e.g. 'Homeostatic process', 'Cellular chemical homeostasis' and 'Cation homeostasis'	32 (e.g. *ATP7B*, *EPAS1* and *FLVCR1*)
DNA and cell cycle [18]	e.g. 'Chromosome condensation', 'Double-strand break repair' and 'DNA replication'	28 (e.g. *CDC6*, *POLA1* and *RAD52*)
Tissue/cell development and differentiation [10]	e.g. 'Metanephric nephron development', 'Ventricular cardiac muscle tissue development' and 'Regulation of organ growth'	17 (e.g. *COL14A1*, *MYBPC3* and *PDGFRA*)
Signalling pathway [4]	e.g. 'Regulation of cytokine-mediated signaling pathway', 'Neuropeptide signaling pathway' and 'Nucleotide-binding domain, leucine rich repeat containing receptor signaling pathway'	14 (e.g. *HPX*, *IFNGR1* and *ROBO1*)
Response and detection [4]	e.g. 'Response to gamma radiation' 'Cellular response to oxidative stress' and 'Detection of chemical stimulus'	13 (e.g. *ATR*, *EPAS1* and *VRK2*)
Organelle fission [1]	'Organelle fission'	13 (e.g. *AKAP8*, *SKA3* and *ZWINT*)
Protein [2]	'Regulation of protein processing' and 'Protein secretion'	10 (e.g. *ARFIP1*, *EXPH5* and *GLG1*)
Glycosylation [3]	'O-glycan processing', 'Protein glycosylation' and 'Macromolecule glycosylation'	9 (e.g. *B3GNT7*, *LRP2* and *MUC13*)
Ions [3]	'Zinc ion transport', 'Sequestering of metal ion' and 'Sequestering of calcium ion	8 (e.g. *ATP7B*, *HRC* and *SH3TC2*)
Fertilization [1]	'Fertilization'	5 (e.g. *DUOX2*, *SPAG1* and *TRIM36*)
Regulation of platelet activation [1]	'Regulation of platelet activation'	*PDGFB*, *PDGFRA* and *TLR4*
Substrate-dependent cell migration [1]	'Substrate-dependent cell migration'	*PDGFB*, *PTPRC* and *ROBO1*
Keratan sulfate [2]	'Keratan sulfate metabolic process' and 'Keratan sulfate biosynthetic process'	*B3GNT7*, *ST3GAL1* and *ST3GAL6*

‘Regulation of triglyceride metabolic process’ [1]	‘Regulation of triglyceride metabolic process’	*C3*, *LMF1* and *NR1H2*
Oligosaccharide metabolic process [1]	‘Oligosaccharide metabolic process’	*PRKCSH*, *ST3GAL6* and *ST8SIA3*

(D) Enriched BP terms based in Star-nosed mole

Category [#terms]	Representative GO Terms	Associated genes
Detection and response [25]	e.g. 'Detection of stimulus', 'Response to UV' and 'Sensory perception'	152 (e.g. *TAS2R4*, *TIMELESS* and *USH1C*)
Digestion and metabolism [32]	e.g. 'Digestion', 'Fat-soluble vitamin metabolic process' and 'Steroid metabolic process'	132 (e.g. *ALDH1L2*, *GLS2* and *SLC5A5*)
Immune response [109]	e.g. 'Inflammatory response', 'Cell killing' and 'Neutrophil mediated immunity'	104 (e.g. *LGMN*, *TYR* and *XAF1*)
Development and differentiation [11]	e.g. 'Epithelial cell development', 'Urogenital system development' and 'Embryo development ending in birth or egg hatching'	73 (e.g. *ERBB2*, *NOTCH2* and *VCL*)
Regulation [9]	e.g. 'Regulation of pH', 'Regulation of locomotion' and 'Regulation of hormone levels'	66 genes (e.g. *COMT*, *RBP4* and *SULF1*)
Cellular component movement [2]	'Cellular component movement' and 'Positive regulation of cellular component movement'	48 (e.g. *ELANE*, *HTT* and *ZP3*)
Secretion [4]	e.g. 'Secretion', 'Secretion by cell' and 'Positive regulation of protein secretion'	40 (e.g. *CA2*, *ENG* and *PSAP*)
Adhesion [2]	'Biological adhesion' and 'Cell adhesion'	37 (e.g. *ADIPOQ*, *BOC* and *ITGAL*)
Cell activation [1]	'Cell activation'	34 (e.g. *ALB*, *LCK* and *SLAMF1*)
Taxis and chemotaxis [5]	e.g. 'Taxis', 'Chemotaxis' and 'Cell chemotaxis'	27 (e.g. *BOC*, *ENAH* and *TTC8*)
Blood [14]	e.g. 'Regulation of systemic arterial blood pressure by circulatory renin-angiotensin', 'Platelet degranulation' and 'Smooth muscle contraction'	26 (e.g. *ALB*, *ENG* and *PSAP*)
Transport [4]	e.g. 'Positive regulation of transmembrane transport', 'Positive regulation of protein transport' and 'Regulation of vesicle-mediated transport'	24 (e.g. *CA2*, *RALB* and *RBP4*)
Exocytosis [2]	'Exocytosis' and 'Positive regulation of exocytosis'	20 (e.g. *EXPH5*, *F2RL1* and *RAB3B*)
Behavior [3]	'Regulation of behaviour', 'Positive regulation of behaviour' and 'Positive regulation of locomotion'	19 (e.g. *ALB*, *ELANE* and *ZP3*)
DNA [3]	'Regulation of DNA recombination', 'DNA-dependent DNA replication' and 'Base-excision repair'	15 (e.g. *BRCA2*, *HELB* and *TK2*)

Aging [3]	'Aging', 'Cell aging' and 'Multicellular organismal aging'	13 (e.g. *CAT*, *HTT* and *POLG*)
Protein [3]	'Protein activation cascade', 'Protein maturation' and 'Protein processing'	12 (e.g. *A2M*, *ENPEP* and *PIK3C3*)
Regulation of action potential [1]	'Regulation of action potential'	11 (e.g. *DSC2*, *KCNC2* and *TG*)
Negative regulation of hydrolase activity [1]	'Negative regulation of hydrolase activity'	11 (e.g. *MDM2*, *MICAL1* and *SFI1*)
Signaling [2]	'Smoothened signaling pathway' and 'Positive regulation of calcium-mediated signalling'	9 (e.g. *BOC*, *HHIP* and *TCTN2*)
Positive regulation of transcription factor import into nucleus [1]	'Positive regulation of transcription factor import into nucleus'	4 (e.g. *CDH1*, *IL23A* and *TLR2*)
Actin filament depolymerisation [1]	'Actin filament depolymerisation'	4 (e.g. *MICAL1*, *SCIN* and *SPTBN1*)
Acrosome reaction [1]	'Acrosome reaction'	*AKAP3*, *PLCD4* and *ZP3*
Mitochondrial DNA [2]	'Mitochondrial DNA metabolic process' and 'Mitochondrial genome maintenance'	*MGME1*, *POLG* and *TK2*

Note – results are based on the remaining genes found to be under significant positive selection, using branch-site models, after filtering alignments for less than four taxa and median site interval between positively selected sites (PSS) ≤ 10.

Supplementary tables S18-21 as separate file: Supplementary_tables_S18_21_TopGo_PAML_controls.xlsx

Supplementary table S18
GO enrichment based on PSGs on the elephant+hyrax branch.
(A) Molecular Function GO terms; (B) ‘Cellular Component’ terms; (C) ‘Biological Process’ GO terms; enriched by at least one of the three tests (classic, elim or weight). Abbreviations: fis – Fisher's exact test. Significant terms ($P < 0.05$) are shown by bold font.

Supplementary table S19
GO enrichment based on PSGs in Guinea pig.
(A) Molecular Function GO terms; (B) ‘Cellular Component’ terms; (C) ‘Biological Process’ GO terms; enriched by at least one of the three tests (classic, elim or weight). Abbreviations: fis – Fisher's exact test. Significant terms ($P < 0.05$) are shown by bold font.

Supplementary table S20
GO enrichment based on PSGs on the mouse+rat branch.
(A) Molecular Function GO terms; (B) ‘Cellular Component’ terms; (C) ‘Biological Process’ GO terms; enriched by at least one of the three tests (classic, elim or weight). Abbreviations: fis – Fisher's exact test. Significant terms ($P < 0.05$) are shown by bold font.

Supplementary table S21
GO enrichment based on PSGs in the common shrew.
(A) Molecular Function GO terms; (B) ‘Cellular Component’ terms; (C) ‘Biological Process’ GO terms; enriched by at least one of the three tests (classic, elim or weight). Abbreviations: fis – Fisher's exact test. Significant terms ($P < 0.05$) are shown by bold font.

Supplementary tables S22–S25 as separate file: Supplementary_table_S22_S25_Convergent_results_subterranean.xlsx

Supplementary table S22
Summed posterior probabilities of convergent and divergent substitutions among pairs of subterranean taxa. Abbreviations: PP – posterior probability.
(A) Golden moles and African mole-rats
(B) Golden moles and spalacids
(C) Golden moles and the star-nosed mole
(D) African mole-rats and spalacids
(E) African mole-rats and the star-nosed mole
(F) Spalacids and the star-nosed mole.

Supplementary table S23
Enriched GO 'Biological Process' terms in genes with shared convergent substitutions among pairs of subterranean taxa with at least one of the three tests (classic, elim or weight). Abbreviations: fis – Fisher's exact test. Significant terms (P < 0.05) are shown by bold font.
(A) Golden moles and African mole-rats
(B) Golden moles and spalacids
(C) Golden moles and the star-nosed mole
(D) African mole-rats and spalacids
(E) African mole-rats and the star-nosed mole.
(F) Spalacids and the star-nosed mole.

Supplementary table S24
Enriched GO 'Cellular Component' terms in genes with shared convergent substitutions among pairs of subterranean taxa with at least one of the three tests (classic, elim or weight). Abbreviations: fis – Fisher's exact test. Significant terms (P < 0.05) are shown by bold font.
(A) Golden moles and African mole-rats
(B) Golden moles and spalacids
(C) Golden moles and the star-nosed mole
(D) African mole-rats and spalacids
(E) African mole-rats and the star-nosed mole.
(F) Spalacids and the star-nosed mole.

Supplementary table S25
Enriched GO 'Molecular Function'' terms in genes with shared convergent substitutions among pairs of subterranean taxa with at least one of the three tests (classic, elim or weight). Abbreviations: fis – Fisher's exact test. Significant terms (P < 0.05) are shown by bold font.
(A) Golden moles and African mole-rats
(B) Golden moles and spalacids
(C) Golden moles and the star-nosed mole
(D) African mole-rats and spalacids
(E) African mole-rats and the star-nosed mole.
(F) Spalacids and the star-nosed mole.

Supplementary tables S26–S29 as separate file: Supplementary_table_S26_S29_Convergent_results_control.xlsx

Supplementary table S26

Summed posterior probabilities of convergent and divergent substitutions among pairs of non-subterranean taxa. Abbreviations: PP – posterior probability.
(A) Elephant + hyrax and guinea pig
(B) Elephant + hyrax and mouse + rat
(C) Elephant + hyrax and common shrew
(D) Guinea pig and mouse + rat
(E) Guinea pig and common shrew
(F) Mouse + rat and the common shrew.

Supplementary table S27

Enriched GO 'Biological Process' terms in genes with shared convergent substitutions among pairs of subterranean taxa with at least one of the three tests (classic, elim or weight). Abbreviations: fis – Fisher's exact test. Significant terms ($P < 0.05$) are shown by bold font.
(A) Elephant + hyrax and guinea pig
(B) Elephant + hyrax and mouse + rat
(C) Elephant + hyrax and common shrew
(D) Guinea pig and mouse + rat
(E) Guinea pig and common shrew
(F) Mouse + rat and the common shrew.

Supplementary table S28

Enriched GO 'Cellular Component' terms in genes with shared convergent substitutions among pairs of subterranean taxa with at least one of the three tests (classic, elim or weight). Abbreviations: fis – Fisher's exact test. Significant terms ($P < 0.05$) are shown by bold font.
(A) Elephant + hyrax and guinea pig
(B) Elephant + hyrax and mouse + rat
(C) Elephant + hyrax and common shrew
(D) Guinea pig and mouse + rat
(E) Guinea pig and common shrew
(F) Mouse + rat and the common shrew.

Supplementary table S29

Enriched GO 'Molecular Function'' terms in genes with shared convergent substitutions among pairs of subterranean taxa with at least one of the three tests (classic, elim or weight). Abbreviations: fis – Fisher's exact test. Significant terms ($P < 0.05$) are shown by bold font.
(A) Elephant + hyrax and guinea pig
(B) Elephant + hyrax and mouse + rat
(C) Elephant + hyrax and common shrew
(D) Guinea pig and mouse + rat
(E) Guinea pig and common shrew
(F) Mouse + rat and the common shrew

Supplementary table S30 as separate file:
Supplementary_table_S30_Counts_BP_terms_Convergent_results.xlsx

Table S30
(A) Pivot table of enriched BP GO terms based on 'convergent' genes found across subterranean taxon pairs

(B) Pivot table of enriched BP GO terms based on 'convergent' genes found across non-subterranean 'control' taxon pairs

Table S31

Convergence values and estimated summed branch lengths for pairs of (1) subterranean, (2) non-subterranean and (3) subterranean + non-subterranean taxa. Group 3 pairs are subdivided into (a) conservative and (b) relaxed controls based on branch length.

Group	Branch 1	BL 1	Branch 2	BL2	Total BL	#genes	#genes PP≥1
1	Golden moles	0.030	African mole-rats	0.001	0.031	7,486	506
	Golden moles	0.030	Spalacids	0.013	0.043	7,085	627
	Golden moles	0.030	Star-nosed mole	0.039	0.069	7,044	2,011
	African mole-rats	0.001	Spalacids	0.013	0.014	7,186	210
	African mole-rats	0.001	Star-nosed mole	0.039	0.040	7,120	735
	Spalacids	0.013	Star-nosed mole	0.039	0.053	6,732	895
2	Elephant + hyrax	0.009	Guinea pig	0.044	0.053	7,622	675
	Elephant + hyrax	0.009	Mouse + rat	0.037	0.045	7,673	648
	Elephant + hyrax	0.009	Common shrew	0.073	0.082	7,889	1,096
	Guinea pig	0.044	Mouse + rat	0.037	0.081	7,627	2,227
	Guinea pig	0.044	Common shrew	0.073	0.117	7,295	3,004
	Mouse + rat	0.037	Common shrew	0.073	0.109	7,337	3,129
3a	Golden moles	0.030	Guinea pig	0.044	0.074	7,479	1,685
	Golden moles	0.030	Mouse + rat	0.037	0.066	7,527	1,765
	Golden moles	0.030	Common shrew	0.073	0.103	7,197	2,668
	Spalacids	0.013	Guinea pig	0.044	0.058	7,152	858
	Star-nosed mole	0.039	Guinea pig	0.044	0.084	7,115	2,132
	Spalacids	0.013	Common shrew	0.073	0.086	6,888	209
3b	African mole-rats	0.001	Elephant + hyrax	0.009	0.010	7,625	171
	Spalacids	0.013	Elephant + hyrax	0.009	0.022	7,200	209
	Star-nosed mole	0.039	Elephant + hyrax	0.009	0.048	7,165	771
	African mole-rats	0.001	Mouse + rat	0.037	0.038	7,628	692
	African mole-rats	0.001	Common shrew	0.073	0.074	7,288	1,119
	Star-nosed mole	0.039	Mouse + rat	0.037	0.076	7,164	2,193

Table S32
Genes identified as under positive selection and undergoing convergent substitutions between subterranean species pairs.

(A) African mole-rats vs. golden moles

Gene	Sites with PP > 0.5 [PP convergence]	African mole-rat PSS	Golden mole PSS
AOC3	377 [1.29]	59, 60, 137, 140, 153, 156, 194, 295, 424, 503, 535, 539, 548	70, 74, 91, 166, 184, 186
C3	780 [1.64]	382, 507, 553, 921, 1292	218, 321, 334, 503, 519, 553, 654, 959, 1013, 1077, 1145, 1236, 1339, 1340, 1343
FAAP24	**30**, 129 [1.72]	102, 172	21, **30**, 126
GPRC5A	**211** [1.31]	40, 111, 143, **211**, 236, 239, 265, 288, 306, 349	6, 27, 30, 133,.165, 183, 201, **211**, 230
LCT	381, 1711 [3.04]	60, 65, 159, 210, 266, 325, 392, 502, 574, 580, 643, 824, 850, 917, 1171, 1253, 1510, 1542, 1552, 1671, 1701, 1748	94, 1589, 1621, 1753
MORC1	300, 330 [1.92]	409	48, 368, 570
PKHD1L1	NA [1.15]	140, 2398	344, 599, 1881
RXFP1	438 [1.28]	NA	58, 62, 162, 184, 215, 242, 498, 508, 604, 607, 689
SLC17A3	495 [2.29]	11, 13, 26, 88, 126, 240, 276	11, 209, 312, 433, 467
SVIL	**1310** [3.69]	163, 299, 1064, **1310**, 1518	153, 209, 213, 214, 310, 892, 1750

(B) Spalacids vs. golden moles

Gene	Sites with PP > 0.5 [PP convergence]]	Spalacids PSS	Golden moles PSS
C3	446, 761 [1.64]	382, 441	218, 321, 334, 503, 519, 553, 654, 959, 1013, 1077, 1145, 1236, 1339, 1340, 1343
PIK3C2G	90, 182 [2.47]	77, 115, 131, 177, 179, 194, 269, 341, 343, 408, 487, 554	34, 190, 593
SYDE2	NA [1.21]	138, 203, 278, 311, 814, 827, 920	247, 874, 911

(C) Star-nosed moles vs. golden moles

Gene	Sites with PP > 0.5 [PP convergence]	Star-nosed mole PSS	Golden mole PSS
ADGRG7	91, 203, 426 [4.02]	4, 13, 36, 65, 70, 122, 128, 135, 278, 411	67, 73, 108, 152, 278, 289
ALB	**236**, 326, 491 [4.25]	366, 423, 494, 498	140, 207, **236**, 255, 266, 278, 303, 517
BRCA2	980, 1045, 1294, 1340, 1751, 1771, 2092 [13.24]	99, 310, 343, 387, 454, 500, 520, 637, 647, 670, 726, 1028, 1035, 1102, 1169, 1190, 1219, 1540, 1586	1146, 1445, 1446, 1960
CCDC79	96, 185, 225 [2.92]	110, 214, 226, 266, 303	3, 63, 163, 184, 213, 231, 237, 267, 272
GSE1	495, 504, 534 [3.26]	296, 299, 306, 368, 416, 686, 715, 729, 784, 789, 799, 881, 900	428, 432, 617, 670, 723, 724, 746, 893, 923
HTT	1442, 1831, 1840, **1847** [7.06]	1148, 1150, 1389, 1393, **1847**, 1849, 1861, 1919	53, 284, 483, 500, 516, 518, 639, 671, 774, 795, 988, 1257, 1360, 1371, 1385, 1463, 1583, 1729, 1730, 1764, 1792, 1828, 1835, **1847**, 1848, 1857, 2049, 2349, 2431
IMPG2	355, 390, 477, 479, 618, 642 [6.21]	540	159, 240, 374, 491
LCT	486, 670, 796 [3.31]	69, 133, 282, 812, 956, 1386, 1436	94, 1589, 1621, 1753
KIF14	65, 250, 327, 810, 1170, 1174 [6.12]	61, 648, 958, 1070, 1071, 1101, 1126, 1191	105, 280, 958, 1123
MISP	20, 55, 83, 99, 143, 295, 331, 368, 372, 409 [8.77]	12, 14, 27, 75, 79, 123, 209, 322, 363, 387, 439, 453, 457, 459	52, 76, 91, 97, 135, 152, 363, 370
MYH14	862, 910, 1228, 1441, **1511**, **1654** [4.92]	62, 376, 1061, 1085, 1278, 1363, 1501, 1503, 1504, 1510, **1511**, 1544, 1561, 1565, 1570, 1591, **1654**, 1808	62, 79, 83, 216, 424, 516, 535, 883, 1028, 1035, 1197, 1573, **1654**
NUPL2	209, 269 [1.81]	55, 80, 257, 290, 345	75, 76, 222
P2RX7	84, 148, 217, 305, **344**, 461 [5.52]	114, 133, 162, 296, 305, 356, 360, 365, 462, 501, 539	**344**, 352, 422, 439, 518
PLA2G1B	94 [1.04]	33, 37, 69, 71, 72, 107, 130	14, 57, 60, 73

PMFBP1	303, 315 [1.72]	228, 361	253, 333, 448
PSAP	63, **88**, 207, 235, 243, 337 [5.25]	7, 38, 44, 55, **88**, 120, 171, 341, 343, 435	28, 87, **88**, 98, 217, 242, 326, 422
PUS7L	148, 305, 582, **621** [3.70]	75, 87, 125, 141, 371, 454, 509, 518, 615, **621**, 622	286, 453, 547
RFWD3	306, 323, 423 [3.62]	14, 27, 127, 139, 145, 290	16, 33, 123, 520
SVIL	841 [2.29]	243, 379, 714, 983	153, 209, 213, 214, 310, 892, 1750
TCHHL1	129, 370, 399, 539, 594, 618 [6.69]	355, 374, 542, 558, 660	227, 294, 303, 347, 411, 438
TFPI2	30 [1.17]	69, 142	19, 117, 159, 163

(D) Star-nosed moles vs. spalacids

**Gene**	**Sites with PP > 0.5 [PP convergence]**	**Star-nosed mole PSS**	**Spalacids PSS**
A2M	423, 425, 632, 770, 896 [4.03]	54, 58, 169, 757	161, 283, 1095, 1109
COBLL1	575 [1.23]	325, 445, 506, 532, 696, 795	890
EFHB	112, 119 [2.66]	12, 14, 27, 68, 109, 286, 443, 444, 480, 558, 560	75, 93, 95, 115, 139
EPAS1	**696** [1.11]	312, 381, 434, 710	351, 381, 540, 570, 646, 695, **696**, 718
EXPH5	334, 483 [4.37]	433, 529, 599, 777, 853, 854, 857	285, 441
GLT6D1	15, 27, 32, 35, 51, 58, 59, 60, 65, 76, **100**, 109, 110, **116**, 133, 137, 142, **151**, 157, 164, 169, 190, 191, 194, 206, 212, 213, 216, 219, 221, **222**, **224** [31.93]	21, 23, 73, **100**, **116**, 135, 162, 163, 174, 209, **222**, 223, **224**	35, 45, 68, **116**, **151**, 162, 174, **222**
ITGAL	194 [2.39]	6, 329, 570, 593, 600, 611, 616, 632, 657, 729, 751, 818, 824	417, 615, 707
LRP2	170, 197, 347, 818, 901, 940, 1127, 1255, 1386, 1449, 1469, 1726, 1808, 2445, 2642, 2751, 2825, 2956, 3016, 3044, 4264, 4273 [19.17]	459, 498, 586, 946, 1027, 1558, 2645, 2838, 3668, 3886	99, 793, 2109, 2343, 2605, 2628, 2917, 3738, 3849
MUC13	47, 162, 178 [3.20]	16, 62, 129, 136, 185, 194, 198	9, 19, 108

NLRC4	303, 332, 462, 563, 618 [6.00]	88, 187, 302, 457, 500, 603, 629, 865, 954	149, 193, 237, 258, 410, 526, 681
PTPRC	3, 65, 98, 110, 143, **524**, 536, 645, 653, 833 [10.32]	28, 75, 76, 100, 111, 130, 223, 245, 247, 272, 396, 401, 404, 421, 450, 452, 474, 485, 496, 500, 502, 517, **524**, 554, 643, 728, 744, 767	109, 145, 333, 691
SCIN	**201**, 326 [1.97]	37, 97, 246, 346, 401, 459, 502	**201**, 219, 607
SHROOM1	NA [1.69]	149, 209, 247, 297, 499, 596	318, 336, 403
TEX15	392, 544, 581, 609, 629, 639, 688, 695, 709, 835, 1285, 1320, 1322 [13.28]	134, 1157, 1237	370, 523, 534, 587, 928, 1093, 1338, 1348
TLR2	NA [1.17]	50, 76, 203, 226, 262, 271, 276, 295, 448, 473, 561, 629, 744, 745	572

(E) Star-nosed moles vs. African mole-rats

**Gene**	**Sites with PP > 0.5 [PP convergence]**	**Star-nosed mole PSS**	**African mole-rats PSS**
ADGRE5	NA [1.45]	134, 145, 153, 216, 380	12, 94, 233
CCDC129	523 [1.39]	130, 209, 241, 306, 367, 509, 519, 559, 582, 637, 642	640
CX3CR1	**56**, 262 [1.90]	14, 36, 288, 314	**56**, 87, 159, 250, 253, 316, 326
DLEC1	NA [1.90]	403, 414, 490, 568, 579, 764, 780	64, 67, 68, 69, 83, 99, 141, 588, 639, 702, 780
ENO4	56, 276, 353 [2.16]	7, 75, 105, 153, 154, 155, 157, 158, 213, 226, 355, 469	55, 292, 469
FAM208B	**1581** [3.71]	353, 658, 956, 1554, 1834, 1999	50, 274, 314, 368, 376, 556, 561, 657, 793, 833, 878, 902, 1040, 1070, 1119, 1204, 1227, 1235, 1284, 1364, 1430, 1451, 1513, 1530, 1548, 1560, **1581**, 1600, 1646, 1648, 1675, 1711, 1770, 1804, 1806, 1932, 1959, 1960, 2017
FGF23	**106 [**1.06]	23, 34	61, **106**, 164, 181
LCT	563 [1.62]	69, 133, 282, 812, 956, 1386, 1436	60, 65, 159, 210, 266, 325, 392, 502, 574, 580, 643, 824, 850, 917, 1171, 1253, 1510, 1542, 1552, 1671, 1701, 1748
MST1	97, 165, 193 [2.57]	50, 352, 421, 462	284, 387, 393, 470, 579
NAALADL1	52 [1.36]	118, 123, 293, 570, 633	46, 84, 308
SVIL	825, **1518**, 1698 [3.83]	243, 379, 714, 983	163, 299, 1064, 1310, **1518**
TEX15	6, 236, 562, 695 [3.63]	134, 1157, 1237	11, 344, 357, 552, 742, 758, 1200
TMEM173	116, 151, 343 [2.31]	5, 109, 184, 292, 359	31, 40, 86, 96, 111, 144, 194, 243, 246, 266, 275, 301, 313
VSIG10L	NA [1.07]	489	2, 3, 11, 99, 478, 583, 607, 654
XAF1	12, 126 [1.64]	98, 117, 179, 204	22, 86, 132

(F) African mole-rats vs. spalacids

Gene	Sites with PP > 0.5 [PP convergence]	African mole-rats PSS	Spalacids PSS
ADGRF1	276 [1.61]	238, 444	212, 429
C3	869 [1.47]	382, 507, 553, 921, 1292	382, 441
ICE1	NA [1.21]	277, 720, 729, 1050, 1445	719, 1172
HOXB6	**61** [1.01]	**61**	**61**, 211
LAMA3	2688 [2.84]	1539, 2134, 2188, 2286, 2527, 2541, 2621, 2671, 2679, 2680, 2729	2193, 2680
TDRD6	**1416**, **1420** [4.63]	121, 150, 286, 417, 432, 437, 686, 962, 1066, 1080, 1380	**1416**, **1420**, 1431, 1447
TEX15	695, 1019, 1046, 1284 [4.44]	11, 344, 357, 552, 742, 758, 1200	370, 523, 534, 587, 928, 1093, 1338, 1348

Note – amino acid sites identified as being under both positive selection and convergence are shown in bold; site number refers to the position in the trimmed alignment and not the complete protein. Positively selected sites are defined as LRT $P<0.05$, $\omega>1.00$ and Bayes Empirical Bayes>0.5.
Abbreviations: PP – posterior proportion; PSS – positively selected site; NA – not applicable.

Table S33

Genes identified as being both under positive selection and undergoing convergent substitutions between subterranean and non-subterranean species pairs.

(A) African mole-rats vs. Elephant+hyrax

Gene	Sites with PP > 0.5 [PP convergence]	African mole-rat PSS	Elephant+hyrax PSS
GPR110	109 [1.66]	238, 444	43, 53, 54, 358, 421, 600, 611, 684
KIAA0947	NA [1.20]	277, 720, 729, 1050, 1445	484, 496, 499, 543, 550, 591, 598, 614, 663, 664, 698, 726, 729, 746, 777, 797, 798, 799, 803, 807, 827, 863, 881, 883, 885, 914, 921, 927, 941, 965, 1000, 1010, 1049, 1132, 1155, 1167, 1334, 1335, 1343, 1351, 1354, 1359, 1397, 1441, 1442, 1460, 1468, 1509, 1512, 1527, 1531, 1539, 1676, 1801, 1831, 1849
ODAM	76 [1.52]	220	6, 96, 97, 130, 132, 138, 187, 204
PKD1L1	1207 [1.10]	368, 380, 499, 511, 581, 608, 985, 1023, 1026, 1028, 1029, 1091, 1132, 1216, 1218, 1219, 1267, 1275	6, 9, 17, 209, 211, 703, 716, 836, 864, 882, 902, 927, 929, 930, 931, 932, 933, 934, 1057, 1071, 1072, 1259, 1271

(B) Star-nosed mole vs. Elephant+hyrax

**Gene**	**Sites with PP > 0.5 [PP convergence]**	**Star-nosed mole PSS**	**Elephant+hyrax PSS**
CD5L	323 [2.36]	50, 67, 99, 148, 168, 259, 282, 318	1, 2, 66, 254, 288, 317, 319, 321
ENTPD1	**279**, **293** [2.25]	81, 241, 299	21, 82, 152, **279**, **293**, 379
GPR128	**376**, 426, 432 [3.09]	4, 13, 36, 65, 70, 122, 128, 135, 278, 411	40, 52, 75, 129, 245, **376**, 428
ITGAL	NA [1.43]	6, 329, 570, 593, 600, 611, 616, 632, 657, 729, 751, 818, 824	48, 123, 171, 226, 414, 417, 419, 534, 613, 667
LPCAT2	**244** [1.20]	73, 89, 146, 307	46, 54, 55, 141, 172, **244**, 265, 370
MYH14	1560 [1.28]	62, 376, 1061, 1085, 1278, 1363, 1501, 1503, 1504, 1510, 1511, 1544, 1561, 1565, 1570, 1591, 1654, 1808	44, 223, 227
MYOM3	1016 [2.37]	75, 91, 215, 216, 229, 231, 232, 235, 237, 239, 241, 288, 307, 467, 521, 757, 948	27, 38, 46, 52, 67, 340, 342, 361, 392, 419, 441, 459, 579, 593, 704, 897, 944
P2RX7	**192**, 259 [2.17]	114, 133, 162, 296, 305, 356, 360, 365, 462, 501, 539	7, 20, 42, 46, 49, 78, 109, **192**, 204, 242, 281, 360, 370, 437, 522
PLXNC1	**111** [1.39]	115, 247, 263, 290, 291, 293, 378, 412, 417, 462, 491, 513, 610	98, **111**, 133, 134, 238, 258, 581
TG	NA [1.29]	18, 203, 225, 306, 503, 592, 643, 749, 763	28, 46, 401, 598, 599, 685, 1100

(C) Common shrew vs. African mole-rats

Gene	Sites with PP > 0.5 [PP convergence]	Common shrew PSS	African mole-rat PSS
ACACB	**1220** [1.77]	30, 32, 693, 958, 1161, 1557	101, 345, 383, 607, 722, 729, 763, 765, 1184, **1220**, 1282, 1320, 1418, 1675
APOB	999, 1221, 1792, 1806, 2640, 2943, 2970, 3563, 3982, 3988 [14.12]	144, 836, 1320, 1583, 1833, 2041, 2186, 3768, 3808, 4121	2154, 2514
C3	1056 [3.68]	62, 99, 103, 195, 492, 548, 549, 555, 564, 574, 615, 639, 682, 714, 734, 755, 760, 779, 899, 990, 994, 1083, 1158, 1256	382, 507, 553, 921, 1292
C5	900 [2.63]	14, 61, 67, 80, 84, 112, 271, 279, 291, 293, 675, 677, 682, 731, 1136	590, 603
CDON	206, 437 [1.60]	8, 503, 513, 783, 786, 787, 861, 951	24, 38, 107, 188, 353, 461, 1084
CLCA1	195, 233, 632, 868 [5.59]	199, 425, 742	83, 176, 178, 256, 350, 616, 618, 761, 833
CXorf22	258, 307, 552 [4.47]	2, 13, 18, 142, 491, 512, 562, 653	378
DLEC1	NA [3.10]	107, 124, 253, 261, 401, 608, 628, 641, 644, 645, 647, 677, 684, 748, 814	64, 67, 68, 69, 83, 99, 141, 588, 639, 702, 780
F11	11, 181 [1.77]	226, 308, 498	7, 130, 196, 203, 215, 274, 407, 471, 485, 559, 598
GPATCH1	403 [1.54]	312, 554, 670	328, 336, 392, 492, 544, 666, 727
IRF3	219 [1.19]	15, 141, 148, 162, 165, 237, 266, 279, 281, 299	20, 93, 191, 192, 217
KIAA1217	**457**, **463** [2.93]	422, 426, 567	7, 292, 323, **457**, **463**, 504, 555, 561
LCT	8, 1650, **1671**, 1749 [5.93]	94, 109, 190, 236, 1037, 1047, 1048, 1106, 1216, 1660, 1689, 1739	60, 65, 159, 210, 266, 325, 392, 502, 574, 580, 643, 824, 850, 917, 1171, 1253, 1510, 1542, 1552, **1671**, 1701, 1748

LRSAM1	NA [1.11]	68, 118, 123, 125, 161, 181, 184, 244, 264, 266, 275, 341, 363, 389, 412, 537	99, 115, 121, 162, 182, 190, 206, 234, 256, 303, 312, 367, 398, 468, 564, 615
NTN5	**67**, **119** [2.05]	3, 13, 44, 48, 53, 72, 121, 124, 274, 300, 303, 318, 382	28, 61, **67**, 79, 92, 102, 107, **119**, 307, 309, 342
OASL	130 [1.74]	51, 115, 134, 273, 306, 317, 318, 350, 370, 411, 414, 423, 455	6, 10, 14, 50, 100, 104, 120, 135, 147, 151, 152, 160, 162, 165, 170, 188, 211, 262, 274, 280, 334, 345, 391, 412, 475, 494, 496
PKD1L1	551, 621, 845, **1023**, 1044, 1078, 1134, 1159 [6.97]	108, 513, 813, 910, 959, 1018, 1123, 1188	368, 380, 499, 511, 581, 608, 985, **1023**, 1026, 1028, 1029, 1091, 1132, 1216, 1218, 1219, 1267, 1275
PKHD1L1	NA [2.67]	818, 1412, 1454, 1461, 1884, 2081, 3121, 3576, 3596	140, 2398
TDRD6	279 [1.19]	918, 949, 962, 971, 1068, 1379	121, 150, 286, 417, 432, 437, 686, 962, 1066, 1080, 1380
TEX15	6, 74, 313, 641, 692, 695, 1034 [6.69]	63, 186, 515, 724, 806, 973, 974, 976, 1095	11, 344, 357, 552, 742, 758, 1200
TICAM1	**289** [2.05]	173, 184, 245, 320, 373, 374, 427, 428, 429	22, 71, 74, 102, 112, 131, 151, 156, 165, 173, 211, 216, 219, 231, 256, **289**, 333, 366, 373, 401, 416, 418, 422, 425
TMEM156	21, 53 [1.62]	52, 140, 147	144, 164
VPS13B	NA [1.13]	492, 890, 1439, 1485, 1501, 2202, 2216, 2880, 2890, 2927	105, 106, 108, 111, 1495, 1644, 2028, 2066, 2150, 2733
VSIG10L	NA [1.38]	422, 479	2, 3, 11, 99, 478, 583, 607, 654

(D) Mouse+rat vs. Star-nosed mole

Gene	Sites with PP > 0.5 [PP convergence]	Mouse+rat PSS	Star-nosed mole PSS
A2M	51, 480, 599, 731, 765, 792, 991, 1164 [8.42]	76, 216, 286, 288, 305, 408, 468, 613, 614, 648, 747, 860, 965, 1125, 1130, 1178, 1182, 1254, 1297	54, 58, 169, 757
ALB	324, 458 [3.85]	253, 409, 572	140, 207, 236, 255, 266, 278, 303, 517
BPIFB1	128, 160, 207 [3.87]	21, 189, 252, 289	20, 50, 63, 81, 83, 93, 107, 127, 183, 216, 224, 239
BRCA2	480, 1195 [16.67]	12, 60, 249, 499, 865, 1084, 1336, 1536, 1540, 1907, 2055, 2097, 2220	99, 310, 343, 387, 454, 500, 520, 637, 647, 670, 726, 1028, 1035, 1102, 1169, 1190, 1219, 1540, 1586
CAMTA2	200, 739 [1.55]	262, 280, 320, 322, 347, 462, 490, 604, 648, 705, 795	754
CCDC175	200, 210, 252, 346, 519, 549, 567, **569** [12.44]	128, 221, 244, 320, 371, 438, 457, 464, 470, 491, 537, 555, 564, **569**, 627, 644, 687, 701	128, 221, 244, 320, 371, 438, 457, 464, 470, 491, 537, 555, 564, **569**, 627, 644, 687, 701
CCDC36	314 [2.12]	50, 113, 132, 134, 175, 253, 309, 310, 369, 412	9, 220, 300, 388
CDH1	285, 306 [3.82]	62, 163, 257, 358, 526, 530, 602	3, 102, 213, 218, 263, 373, 465, 507, 660
CEP152	63, 438, 662 [4.70]	303, 731, 935, 936, 937, 961	107, 165, 366, 369, 409, 410, 723, 1038, 1150
ESCO1	80, 372 [2.23]	64, 156, 197, 363	286, 351
EXPH5	146, 447, 472, 758, 899, 954 [7.71]	139, 467, 781	433, 529, 599, 777, 853, 854, 857
FAM115C	56, 111, 153, 164, 290, 367, 434, 480, 509, 512, 599, 754 [9.70]	103, 376, 395, 482, 600, 657, 678, 703, 728, 759	106, 284, 303, 352, 360, 416, 676, 731
GPR128	132, 224, 248, 259, 310, 340, 432 [6.16]	2, 93, 126, 129, 268, 358	4, 13, 36, 65, 70, 122, 128, 135, 278, 411

IKBKAP	25, 39, 141, 266, 475, 505, 562, 601 [6.87]	101, 124, 316, 385, 545, 620, 819, 967	421, 761, 926
IMPG2	20, 131, 171, 184, 390, 522, 555 [6.40]	25, 55, 182, 200, 249, 541, 597, 612, 874	540
ITGAL	72, 166, 354, 390 [4.43]	12, 125, 632, 668, 671	6, 329, 570, 593, 600, 611, 616, 632, 657, 729, 751, 818, 824
LAX1	158, **267**, 288 [3.13]	67, **267**	25, 54, 61, 88, 148, 154, 166, 189, 205, 207, 226, 239, 246, 248, 263, **267**, 268
LCT	1132 [3.89]	1175	69, 133, 282, 812, 956, 1386, 1436
LRRC63	89, **97**, 210, 344 [5.01]	22, 100, 123, 130, 289	82, **97**, 140, 143, 148, 167, 182, 212, 213, 250, 272, 281, 312, 344, 360
MYO1A	14, 236, 299, 310, 324, 354, 470 [6.67]	220, 580, 637, 718	400, 436, 474, 475, 528, 696, 727
MYOM3	47, 437, 843, 878, 1037 [4.66]	58, 761	75, 91, 215, 216, 229, 231, 232, 235, 237, 239, 241, 288, 307, 467, 521, 757, 948
NIN	174, 566, 580, 888, 961, 977, 1465, 1510, 1653, 1763 [9.19]	502, 887, 893, 1017, 1114, 1236, 1240, 1302, 1618, 1637, 1647	639, 1509, 1516, 1616
P2RX7	532 [1.69]	123, 482, 562	114, 133, 162, 296, 305, 356, 360, 365, 462, 501, 539
PLEKHA4	382 [1.05]	249, 550, 583, 613, 619, 621	22, 226, 341, 369, 440, 635
PNKP	100 [1.11]	84, 92, 348	16, 63, 112, 136
SDR39U1	73 [1.09]	6, 29	29, 112, 127
SFI1	423, 553, 675, 950 [3.95]	332, 543	133, 362, 466, 499, 595, 611, 645, 764
SHROOM1	137, 199, 395, 487, 672 [5.31]	8, 108, 244, 262, 316, 321, 508, 509	149, 209, 247, 297, 499, 596
SPEF2	NA [4.20]	39, 275, 586, 597, 757, 841	797, 974, 1064
TAS2R4	NA [1.26]	136, 185	33, 61, 128, 135, 260
TCHHL1	195, 336 [5.32]	111, 173, 178, 192, 204, 260, 289, 302, 347, 362, 369, 418, 444, 461, 476	355, 374, 542, 558, 660

TEX15	22, 174, 327, 422, 774, 905, 1195 [8.76]	165, 233, 547, 665, 1104, 1183, 1229, 1279, 1333	134, 1157, 1237
TRADD	180 [1.27]	110, 127, 178	124, 130, 179, 222, 293
TTF2	98, **119**, 236, 545 [5.88]	120, 510, 645, 665	**119**, 120, 139, 151, 169, 219, 245, 257, 261, 353, 357, 373, 377, 419, 498, 538, 554, 572, 574, 579, 583, 584, 586, 587, 730, 789, 817
USP26	6, 17, 75, 99, 130, 191, 225, 255, 323, 331, 367, 374, 394, 418, 457, 483, 503, 517, 630, 698 [22.79]	118, 119, 139, 181, 184, 219, 298, 556, 558, 577, 650	31, 68, 151, 281, 381, 394, 547, 632
VWA5B1	539, 570, 645, 753, 774 [5.52]	89, 198, 782, 785	175, 582, 823
XPNPEP2	21 [1.80]	5, 274	211, 249, 250, 359, 594, 600, 642

(E) Spalax vs. Elephant+hyrax

**Gene**	**Sites with PP > 0.5 [PP convergence]**	**Spalax PSS**	**Elephant+hyrax PSS**
KIAA0947	**883**, 1712 [6.89]	719, 1172	484, 496, 499, 543, 550, 591, 598, 614, 663, 664, 698, 726, 729, 746, 777, 797, 798, 799, 803, 807, 827, 863, 881, **883**, 885, 914, 921, 927, 941, 965, 1000, 1010, 1049, 1132, 1155, 1167, 1334, 1335, 1343, 1351, 1354, 1359, 1397, 1441, 1442, 1460, 1468, 1509, 1512, 1527, 1531, 1539, 1676, 1801, 1831, 1849

(F) Mouse+rat vs. African mole-rats

Gene	Sites with PP > 0.5 [PP convergence]	Mouse+rat PSS	African mole-rat PSS
APOB	999, 1954, 2001, 2020, 2129 [8.97]	552, 1955, 2291, 2293, 2972, 3878, 3899, 3976, 4122, 4148	2154, 2514
BPIFB1	175 [1.40]	21, 189, 252, 289	44, 284, 298, 302
CENPC	388 [1.67]	9, 45, 116, 119, 132, 227, 319, 391, 422, 476	173, 295, 325, 437, 518
GPR113	NA [1.33]	134, 466	268, 362, 390, 460
IL31RA	23, 464 [2.81]	30, 48, 91, 136, 199, 271, 332, 398, 430	70, 212, 425
LCT	NA [1.32]	1175	60, 65, 159, 210, 266, 325, 392, 502, 574, 580, 643, 824, 850, 917, 1171, 1253, 1510, 1542, 1552, 1671, 1701, 1748
MCM9	NA [2.44]	616, 648, 681, 745, 760	27, 63, 92, 136, 148, 151, 222, 267, 270, 418, 567, 661, 680, 703, 718, 734
PKD1L1	755, 1006 [6.04]	148, 275, 289, 415, 540, 545, 559, 800, 851, 1075, 1094	368, 380, 499, 511, 581, 608, 985, 1023, 1026, 1028, 1029, 1091, 1132, 1216, 1218, 1219, 1267, 1275
TEX15	1205, 1355 [3.22]	165, 233, 547, 665, 1104, 1183, 1229, 1279, 1333	11, 344, 357, 552, 742, 758, 1200

Note – amino acid sites identified as being under both positive selection and convergence are shown in bold; site number refers to the position in the trimmed alignment and not the complete protein. Positively selected sites are defined as LRT $P<0.05$, $\omega>1.00$ and Bayes Empirical Bayes>0.5.
Abbreviations: PP – posterior proportion; PSS – positively selected site; NA – not applicable.

Table S34

Genes identified as being both under positive selection and undergoing convergent substitutions between subterranean and non-subterranean species pairs.

Gene	Taxa pair	Sites with PP > 0.5 [PP convergence]	Lineage (1) PSS	Lineage (2) PSS
ENTPD1	SNM vs. E+H	**279**, **293** [2.25]	81, 241, 299	21, 82, 152, **279**, **293**, 379
GPR128	SNM vs. E+H	**376**, 426, 432 [3.09]	4, 13, 36, 65, 70, 122, 128, 135, 278, 411	40, 52, 75, 129, 245, **376**, 428
LPCAT2	SNM vs. E+H	**244** [1.20]	73, 89, 146, 307	46, 54, 55, 141, 172, **244**, 265, 370
P2RX7	SNM vs. E+H	**192**, 259 [2.17]	114, 133, 162, 296, 305, 356, 360, 365, 462, 501, 539	7, 20, 42, 46, 49, 78, 109, **192**, 204, 242, 281, 360, 370, 437, 522
PLXNC1	SNM vs. E+H	**111** [1.39]	115, 247, 263, 290, 291, 293, 378, 412, 417, 462, 491, 513, 610	98, **111**, 133, 134, 238, 258, 581
ACACB	CS vs. AMR	**1220** [1.77]	30, 32, 693, 958, 1161, 1557	101, 345, 383, 607, 722, 729, 763, 765, 1184, **1220**, 1282, 1320, 1418, 1675
KIAA1217	CS vs. AMR	**457**, **463** [2.93]	422, 426, 567	7, 292, 323, **457**, **463**, 504, 555, 561
LCT	CS vs. AMR	8, 1650, **1671**, 1749 [5.93]	94, 109, 190, 236, 1037, 1047, 1048, 1106, 1216, 1660, 1689, 1739	60, 65, 159, 210, 266, 325, 392, 502, 574, 580, 643, 824, 850, 917, 1171, 1253, 1510, 1542, 1552, **1671**, 1701, 1748
NTN5	CS vs. AMR	**67**, **119** [2.05]	3, 13, 44, 48, 53, 72, 121, 124, 274, 300, 303, 318, 382	28, 61, **67**, 79, 92, 102, 107, **119**, 307, 309, 342
PKD1L1	CS vs. AMR	551, 621, 845, **1023**, 1044, 1078, 1134, 1159 [6.97]	108, 513, 813, 910, 959, 1018, 1123, 1188	368, 380, 499, 511, 581, 608, 985, **1023**, 1026, 1028, 1029, 1091, 1132, 1216, 1218, 1219, 1267, 1275
TICAM1	CS vs. AMR	**289** [2.05]	173, 184, 245, 320, 373, 374, 427, 428, 429	22, 71, 74, 102, 112, 131, 151, 156, 165, 173, 211, 216, 219, 231, 256, **289**, 333, 366, 373, 401, 416, 418, 422, 425
CCDC175	M+R vs. SNM	200, 210, 252, 346, 519, 549, 567, **569** [12.44]	128, 221, 244, 320, 371, 438, 457, 464, 470, 491, 537, 555, 564, **569**, 627, 644, 687, 701	128, 221, 244, 320, 371, 438, 457, 464, 470, 491, 537, 555, 564, **569**, 627, 644, 687, 701

LAX1	M+R vs. SNM	158, **267**, 288 [3.13]	67, **267**	25, 54, 61, 88, 148, 154, 166, 189, 205, 207, 226, 239, 246, 248, 263, **267**, 268
LRRC63	M+R vs. SNM	89, **97**, 210, 344 [5.01]	22, 100, 123, 130, 289	82, **97**, 140, 143, 148, 167, 182, 212, 213, 250, 272, 281, 312, 344, 360
TTF2	M+R vs. SNM	98, **119**, 236, 545 [5.88]	120, 510, 645, 665	**119**, 120, 139, 151, 169, 219, 245, 257, 261, 353, 357, 373, 377, 419, 498, 538, 554, 572, 574, 579, 583, 584, 586, 587, 730, 789, 817
KIAA0947	Sp vs. E+H	**883**, 1712 [6.89]	719, 1172	484, 496, 499, 543, 550, 591, 598, 614, 663, 664, 698, 726, 729, 746, 777, 797, 798, 799, 803, 807, 827, 863, 881, **883**, 885, 914, 921, 927, 941, 965, 1000, 1010, 1049, 1132, 1155, 1167, 1334, 1335, 1343, 1351, 1354, 1359, 1397, 1441, 1442, 1460, 1468, 1509, 1512, 1527, 1531, 1539, 1676, 1801, 1831, 1849

Note – amino acid sites identified as being under both positive selection and convergence are shown in bold; site number refers to the position in the trimmed alignment and not the complete protein. Positively selected sites are based on LRT $P<0.05$; $\omega>1.00$ and Bayes Empirical Bayes>0.5. Abbreviations: PP – posterior proportion; PSS – positively selected site; NA – not applicable; SNM – star-nosed mole; GM – golden moles; AMR – African mole-rats, Sp – Spalacids; E+H – elephant+hyrax; CS – common shrew; M+R – mouse+rat.

Supplementary figures and legends

Figure S1
Schematic diagram of data processing and analytical steps: (1) dataset assembly; (2) ortholog identification and alignment construction; (3) screen for positive selection and alignment errors; (4) calculation of convergent amino acid substitutions and (5) association of positive selection and convergent substitutions. Abbreviations: CDS – coding DNA sequences; PSS – positively selected sites; GO – gene ontology; PP – posterior probability; BEB – Bayes empirical Bayes.

Figure S2
UpSet plots of the protein coding gene content of the transcriptome datasets used in the current study.
(A) By individual species: H. emi – *Heliophobius emini*; N. gal – *Nannospalax galili*; G. cap – *Georychus capensis*; C. mah – *Cryptomys hottentotus mahali*; B. sui – *Bathyergus suillus*; F. dam – *Fukomys damarensis*; C. nat – *Cryptomys h. natalensis*; A. hot – *Amblysomus hottentotus*; C. pre – *Cryptomys h. pretoriae*; T. spl – *Tachyoryctes splendens*; E. bai – *Eospalax baileyi*; R. pru – *Rhizomys pruinosus*; H. gla – *Heterocephalus glaber*. The source of the data is represented as follows: black – genome-wide gene predictions; red – brain and liver transcriptomes; blue – brain, liver, lung, skeletal muscle, heart and kidney transcriptome; yellow – brain and skeletal muscle transcriptome.
(B) Grouped by family level: yellow – golden moles; blue – African mole-rats; orange – spalacids.

Figure S3
Histograms of the total number of species (including focal and background species) per gene alignment across the four date sets screened for positive selection. Data sets are coloured as follows: (i) yellow – golden moles + outgoups; (ii) blue – African mole-rats + outgroups; (iii) orange – spalacids + outgroups and (iv) purple – star-nosed mole + outgroups.

Figure S4
UpSet plots of the positively selected genes in non-subterranean lineages.
(A) Comparison of positively selected genes detected in four subterranean and four non-subterranean lineages: (i) golden moles vs. the elephant+hyrax; (ii) African mole-rats vs. guinea pig; (iii) Spalax vs. mouse+rat; (iv) star-nosed mole vs. common shrew. The four subterranean lineages are coloured as follows: yellow – golden moles; blue – African mole-rats; orange – spalacids and purple – star-nosed mole, the corresponding non-subterranean lineages are shown in grey, and the overlap of genes in black.
(B) Comparison of pooled positively selected genes in subterranean and non-subterranean lineages
(C) Number and overlap of positively selected genes in each of the four non-subterranean lineages, visualised as an UpSet plot (Lex et al. 2014). Abbreviations: PSGs – positively selected genes. The four genes found to be under positive selection across all four lineages are highlighted by the white box.

Figure S5

(A) Identity of common genes under positive selection across the four subterranean lineages. Subterranean lineages are represented as follows: yellow – Chrysochloridae; blue – Bathyergidae; orange – Spalacidae and purple – Talpidae), and overlap in respective sets are indicated by grey symbols.

(B) Identity of shared enriched 'Biological Process' Go terms across the four subterranean lineages. Abbreviations: MP – metabolic process; CP – catabolic process; VEGF – Vascular endothelial growth factor; IR – immune response; TF – transcription factor; Reg. – regulation; Prod. – production; BP – biological process.

(C) Identity of shared enriched 'Molecular Function' Go terms across the four subterranean lineages. Abbreviations: TTA – transmembrane transporter activity.

(D) Identity of shared enriched 'Cellular Component' Go terms across the four subterranean lineages.

Figure S6

Venn diagram of shared enriched 'Biological Process' Go terms across the four non-subterranean lineages. The single enriched term 'Response to cytokine' is shared across all four lineages. Abbreviations: BP – biological process.

Figure S7

Exploring the relationship between estimated total convergence and branch lengths in pairs of taxa.

(A) Optimised ML branch lengths estimated with RAxMLv.7.2.8, using the constrained species topology and a concatenated amino acid alignment of 429 genes (236,199 amino acids) containing all 41 taxa. Branches are labelled according to Fig. 1, and images of animals depict example focal and control taxa.

(B) Plot of estimated convergence against the summed branch length of pairs. Points are coloured as follows: orange – subterranean pairs; blue – 'control' non-subterranean pairs; yellow – 'relaxed' mixed subterranean and non-subterranean; green – 'conservative' mixed subterranean and non-subterranean. The mixed 'control' (terrestrial + subterranean taxa) pairs were assigned to either 'conservative' (long branches) or 'relaxed' (short branches) based on the summed branch lengths of the pairs using the branch length information from (A). Across all groups, a significant positive relationship was found between the number of genes showing evidence of convergent substitutions and summed branch length (Number of 'convergent' genes = 27002 x Branch length – 398, $R^2 = 0.709$, $F = 53.66$ (df 1, 22), $P = 2.47 \times 10^{-7}$).

Figure S8

(A) Number and overlap of proteins identified as sharing convergent amino acid substitutions, based on the summed posterior probability (PP) of convergent substitutions ≥ 1.00, between pairs of non-subterranean lineages, visualised as an UpSet plot. The 130 'common' proteins, identified across all six pairs, are highlighted by the grey box. Abbreviations: CGs – convergent genes.

(B) Number and overlap of proteins identified as sharing convergent amino acid substitutions, based on the summed posterior probability (PP) of convergent substitutions ≥ 1.00, between mixed pairs of subterranean and non-subterranean lineages, visualised as an UpSet plot. The 13 'common' proteins, identified across all six pairs, are highlighted by the grey box. Abbreviations: CGs – convergent genes.

(C) Intersection of 'convergent' genes identified across all six pairs of each of three phenotypic groups: (1) subterranean species (grey circle), (2) non-subterranean control taxa (green circle) and (3) mixed subterranean and non-subterranean taxa (red circle). Animal images provide on example of the species pair contained in each data set.

Figure S9

Histograms of the frequency of taxa pairs that enriched GO Biological Process terms relating to blood, sensory systems and immune response were shared. The top row of plots (i–iii) refers to subterranean taxa, and the lower row of plots (iv–vi) to non-subterranean pairs, in each case the maximum number of pairs that a terms may occur in is six. GO terms were grouped into three categories blood (red), sensory (blue) and immune response (yellow) based on key words and phrases.

Figure S10

(A) Venn diagrams representing overlap between genes under positive selection in two non-subterranean lineages and the presence of convergent substitutions shared between these two lineages. Positive selection in each lineage is coloured as follows: green – Elephant+hyrax; red – Guinea pig; yellow – Mouse+rat and dark blue – Common shrew. Grey circles represent the number of genes with convergent substitutions between each respective pair of taxa.

(B) Venn diagrams representing overlap between genes under positive selection in one subterranean and one non-subterranean lineage and the presence of convergent substitutions shared between these two lineages. Positive selection in each subterranean lineage is coloured as following: green – Elephant+hyrax; blue – Bathyergidae; orange – Spalacidae; dark blue – Common shrew; yellow – Mouse+rat and purple – Talpidae. Grey circles represent the number of genes with convergent substitutions between each respective pair of taxa.

Supplementary References:

Alexa A, Rahnenfuhrer J. 2010. topGO: enrichment analysis for gene ontology. R package version 2160.

Benjamini Y, Hochberg Y. 1995. Controlling the false discovery rate – a practical and powerful approach to multiple testing. J Roy Stat Soc B Met 57: 289–300.

Blanga-Kanfi S, Miranda H, Penn O, Pupko T, DeBry RW, Huchon D. 2009. Rodent phylogeny revised: analysis of six nuclear genes from all major rodent clades. BMC Evol Biol 9: 71.

Castoe TA, de Koning APJ, Kim HM, Gu WJ, Noonan BP, Naylor G, Jiang ZJ, Parkinson CL, Pollock DD. 2009. Evidence for an ancient adaptive episode of convergent molecular evolution. Proc Natl Acad Sci U S A 106: 8986–8991.

Davies KTJ, Bennett NC, Tsagkogeorga G, Rossiter SJ, Faulkes CG. 2015. Family wide molecular adaptations to underground life in African mole-rats revealed by phylogenomic analysis. Mol Biol Evol 32: 3089–3107.

Davies KTJ, Tsagkogeorga G, Bennett NC, Dávalos LM, Faulkes CG, Rossiter SJ. 2014. Molecular evolution of growth hormone and insulin-like growth factor 1 receptors in long-lived, small-bodied mammals. Gene 549: 228–236.

Flicek P, Amode MR, Barrell D, Beal K, Billis K, Brent S, Carvalho-Silva D, Clapham P, Coates G, Fitzgerald S, et al. 2014. Ensembl 2014. Nucleic Acids Res 42: D749–D755.

Grabherr MG, Haas BJ, Yassour M, Levin JZ, Thompson DA, Amit I, Adiconis X, Fan L, Raychowdhury R, Zeng Q, et al. 2011. Full-length transcriptome assembly from RNA-seq data without a reference genome. Nat Biotechnol 29: 644–652.
Keane M, Craig T, Alföldi J, Berlin AM, Johnson J, Seluanov A, Gorbunova V, Di Palma F, Lindblad-Toh K, Church GM, et al. 2014. The Naked Mole Rat Genome Resource: facilitating analyses of cancer and longevity-related adaptations. Bioinformatics 30: 3558–3560.
Lex A, Gehlenborg N, Strobelt H, Vuillemot R, Pfister H. 2014. UpSet: Visualization of Intersecting Sets. IEEE Transactions on Visualization and Computer Graphics (InfoVis '14) 20: 1983–1992.
Lin G-H, Wang K, Deng X-G, Nevo E, Zhao F, Su J-P, Guo S-C, Zhang T-Z, Zhao H. 2014. Transcriptome sequencing and phylogenomic resolution within Spalacidae (Rodentia). BMC Genomics 15: 32.
Löytynoja A, Goldman N. 2005. An algorithm for progressive multiple alignment of sequences with insertions. Proc Natl Acad Sci USA 102: 10557–10562.
Malik A, Korol A, Hübner S, Hernandez AG, Thimmapuram J, Ali S, Glaser F, Paz A, Avivi A, Band M. 2011. Transcriptome sequencing of the blind subterranean mole rat, *Spalax galili*: utility and potential for the discovery of novel evolutionary patterns. PLoS ONE 6: e21227.
Meredith RW, Janecka JE, Gatesy J, Ryder OA, Fisher CA, Teeling EC, Goodbla A, Eizirik E, Simao TLL, Stadler T, et al. 2011. Impacts of the Cretaceous Terrestrial Revolution and KPg extinction on mammal diversification. Science 334: 521–524.
Penn O, Privman E, Landan G, Graur D, Pupko T. 2010. An alignment confidence score capturing robustness to guide-tree uncertainty. Mol Biol Evol 27: 1759–1767.
R Development Core Team. 2012. R: A language and environment for statistical computing. Vienna, Austria.
Stamatakis A. 2006. RAxML-VI-HPC: maximum likelihood-based phylogenetic analyses with thousands of taxa and mixed models. Bioinformatics 22: 2688–2690.
Tsagkogeorga G, McGowen MR, Davies KTJ, Jarman S, Polanowski A, Bertelsen MF, Rossiter SJ. 2015. A phylogenomic analysis of the role and timing of molecular adaptation in the aquatic transition of cetartiodactyl mammals. Royal Soc Open Sci 2: 150156.
Wang M, Zhao Y, Zhang B. 2015. Efficient test and visualization of multi-set intersections. Sci Rep 5: 16923.
Waterhouse RM, Seppey M, Simão FA, Manni M, Ioannidis P, Klioutchnikov G, Kriventseva EV, Zdobnov EM. 2017. BUSCO applications from quality assessments to gene prediction and phylogenomics. Mol Biol Evol msx319: 10.1093/molbev/msx1319.
Yang Y, Smith SA. 2013. Optimizing de novo assembly of short-read RNA-seq data for phylogenomics. BMC Genomics 14: 328.
Yang ZH. 2007. PAML 4: Phylogenetic analysis by maximum likelihood. Mol Biol Evol 24: 1586–1591.
Zhang JZ, Nielsen R, Yang ZH. 2005. Evaluation of an improved branch-site likelihood method for detecting positive selection at the molecular level. Mol Biol Evol 22: 2472–2479.

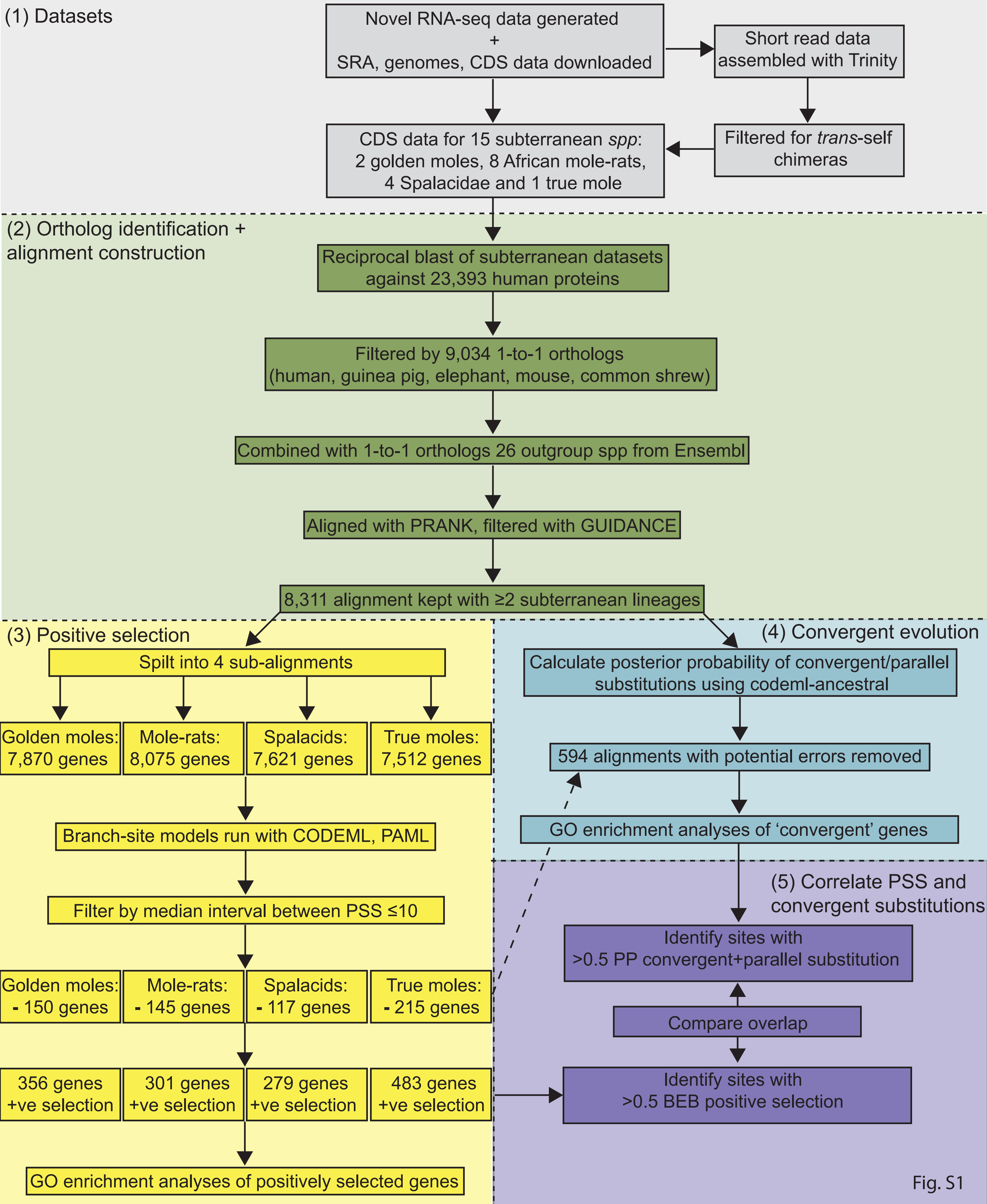
(1) Datasets
Novel RNA-seq data generated + SRA, genomes, CDS data downloaded
Short read data assembled with Trinity
Filtered for trans-self chimeras
CDS data for 15 subterranean spp: 2 golden moles, 8 African mole-rats, 4 Spalacidae and 1 true mole
(2) Ortholog identification + alignment construction
Reciprocal blast of subterranean datasets against 23,393 human proteins
Filtered by 9,034 1-to-1 orthologs (human, guinea pig, elephant, mouse, common shrew)
Combined with 1-to-1 orthologs 26 outgroup spp from Ensembl
Aligned with PRANK, filtered with GUIDANCE
8,311 alignment kept with ≥2 subterranean lineages
(3) Positive selection
Spilt into 4 sub-alignments
Golden moles: 7,870 genes
Mole-rats: 8,075 genes
Spalacids: 7,621 genes
True moles: 7,512 genes
Branch-site models run with CODEML, PAML
Filter by median interval between PSS ≤10
Golden moles: - 150 genes
Mole-rats: - 145 genes
Spalacids: - 117 genes
True moles: - 215 genes
356 genes +ve selection
301 genes +ve selection
279 genes +ve selection
483 genes +ve selection
GO enrichment analyses of positively selected genes
(4) Convergent evolution
Calculate posterior probability of convergent/parallel substitutions using codeml-ancestral
594 alignments with potential errors removed
GO enrichment analyses of 'convergent' genes
(5) Correlate PSS and convergent substitutions
Identify sites with >0.5 PP convergent+parallel substitution
Compare overlap
Identify sites with >0.5 BEB positive selection

Fig. S1

Fig. 2A

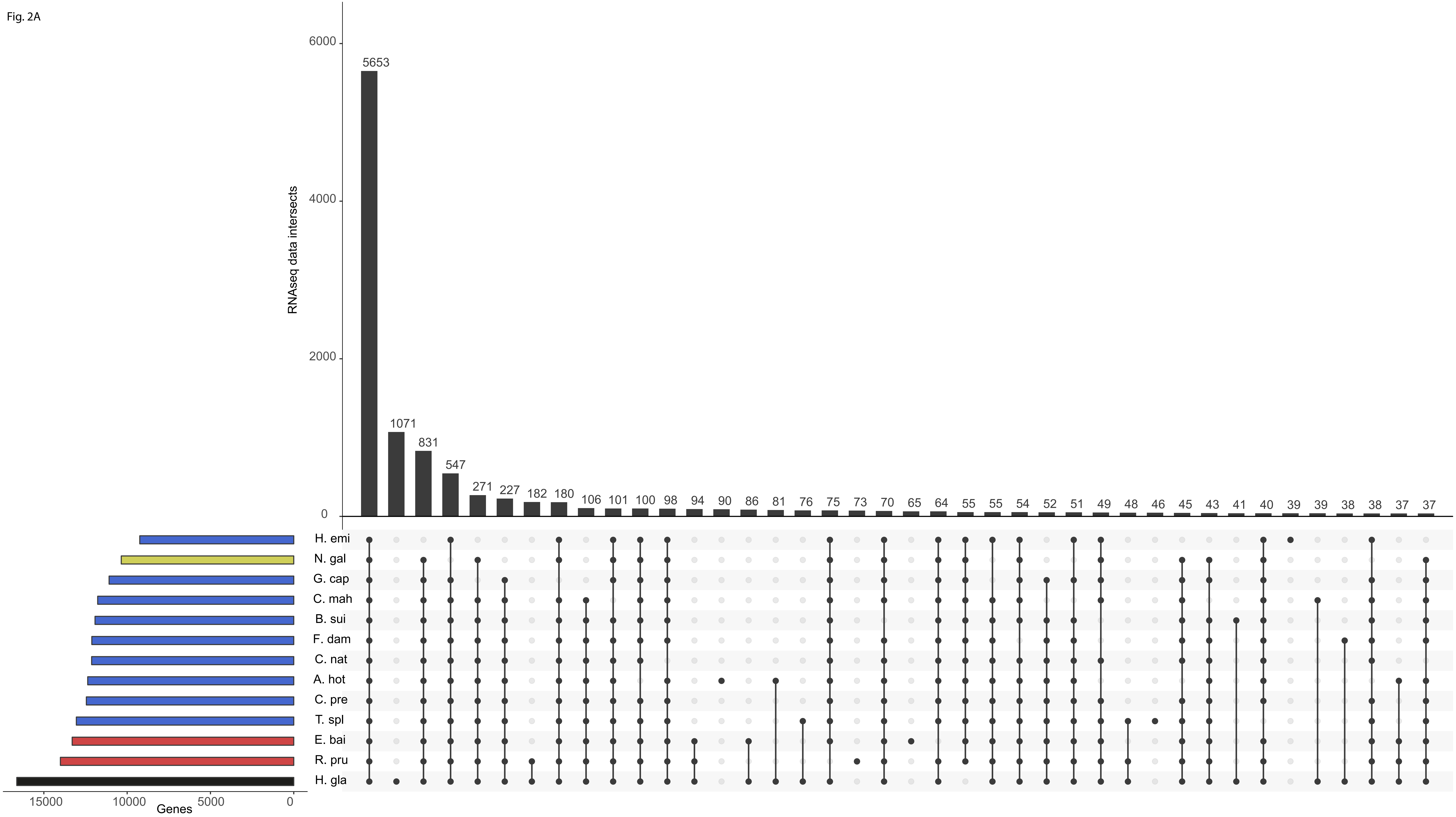
RNAseq data intersects
6000
4000
2000
0
5653 1071 831 547 271 227 182 180 106 101 100 98 94 90 86 81 76 75 73 70 65 64 55 55 54 52 51 49 48 46 45 43 41 40 39 39 38 38 37 37
H. emi
N. gal
G. cap
C. mah
B. sui
F. dam
C. nat
A. hot
C. pre
T. spl
E. bai
R. pru
H. gla
15000
10000
5000
0
Genes

Fig. 2B

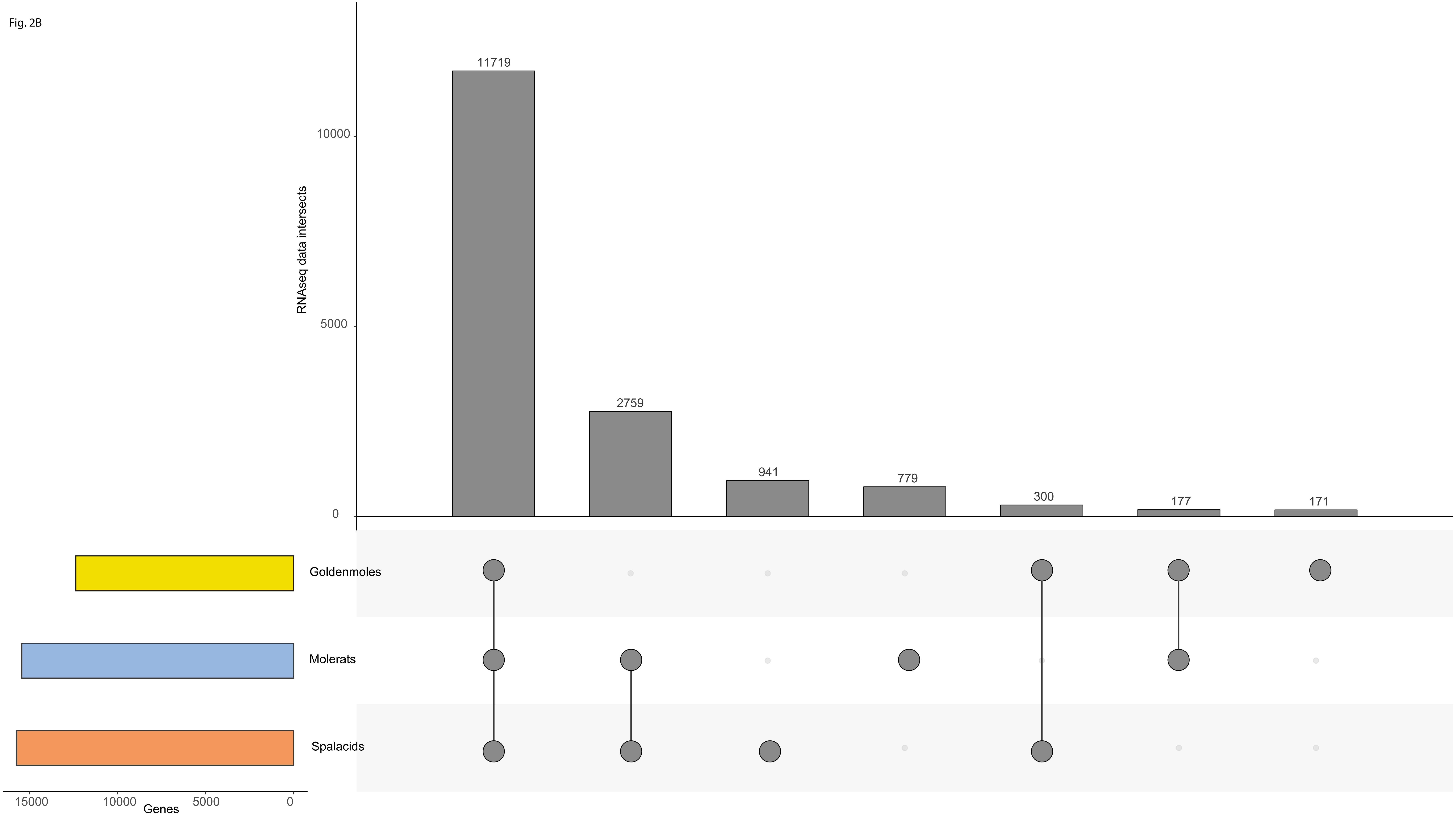
11719
2759
941
779
300
177
171
10000
5000
0
RNAseq data intersects
Goldenmoles
Molerats
Spalacids
15000
10000
5000
0
Genes

Fig. S3

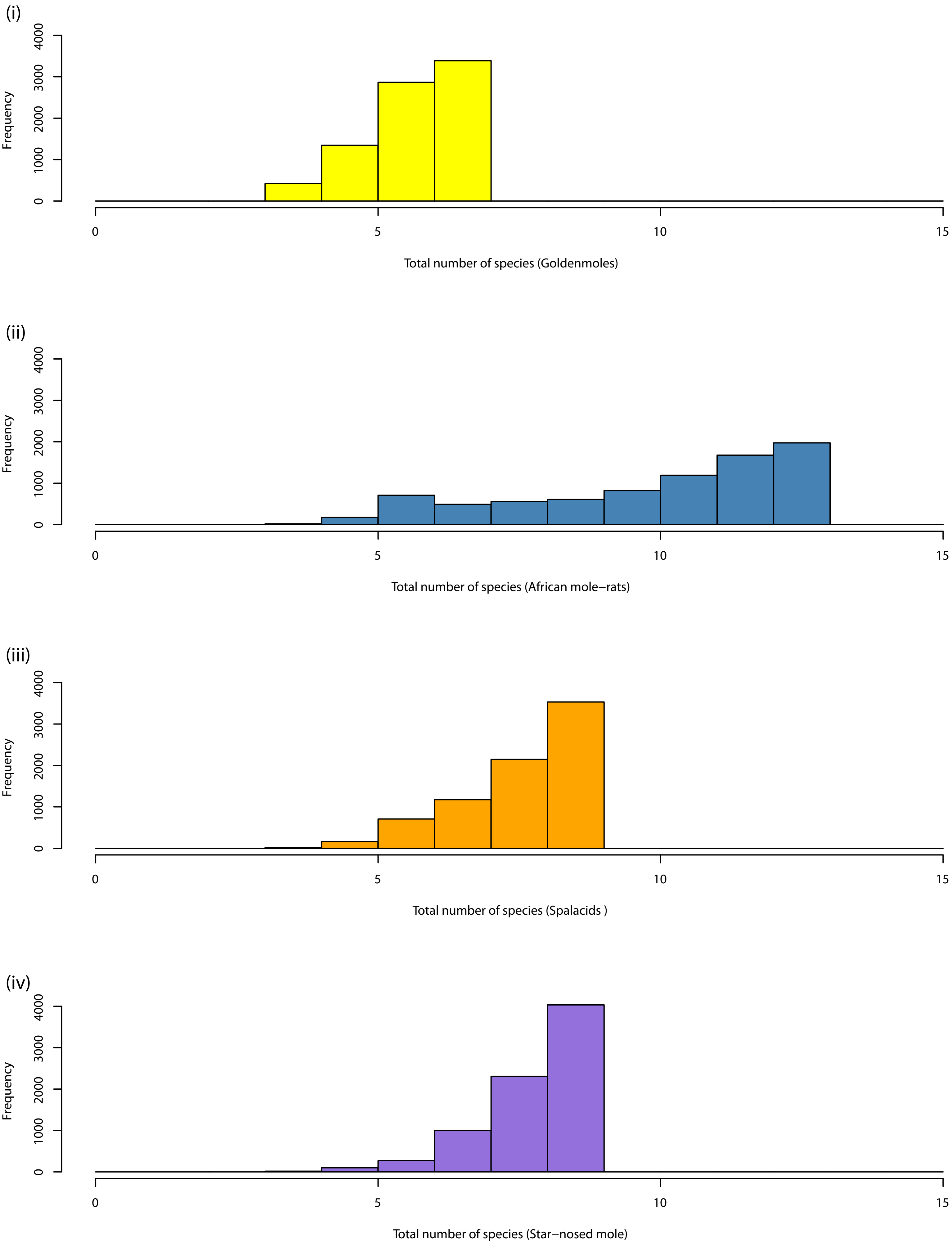

(i)
Frequency
0
1000
2000
3000
4000
0
5
10
15
Total number of species (Goldenmoles)
(ii)
Frequency
0
1000
2000
3000
4000
0
5
10
15
Total number of species (African mole−rats)
(iii)
Frequency
0
1000
2000
3000
4000
0
5
10
15
Total number of species (Spalacids)
(iv)
Frequency
0
1000
2000
3000
4000
0
5
10
15
Total number of species (Star−nosed mole)

Fig. S4A

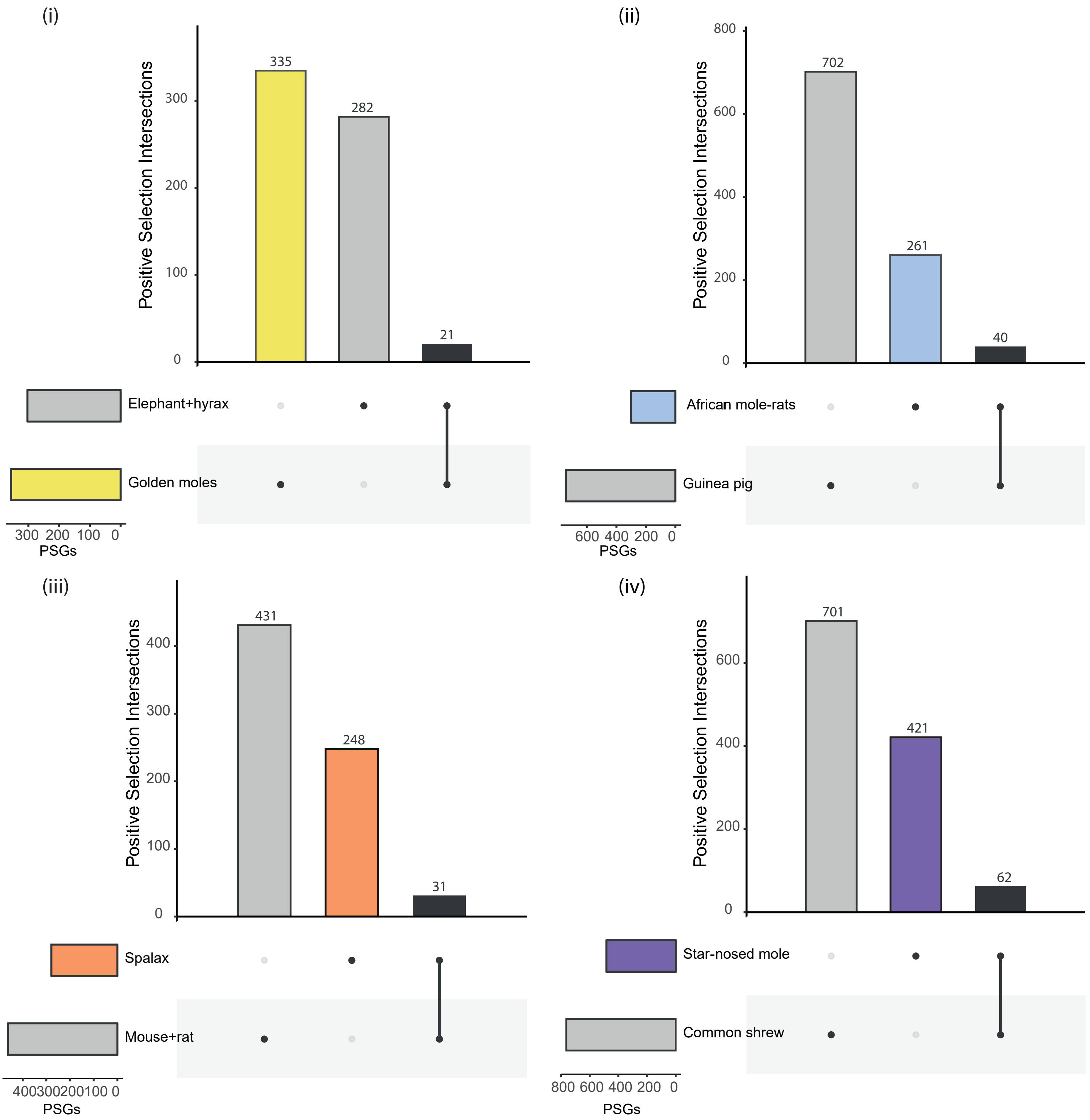
(i)
Positive Selection Intersections
335
282
21
Elephant+hyrax
Golden moles
PSGs
(ii)
Positive Selection Intersections
702
261
40
African mole-rats
Guinea pig
PSGs
(iii)
Positive Selection Intersections
431
248
31
Spalax
Mouse+rat
PSGs
(iv)
Positive Selection Intersections
701
421
62
Star-nosed mole
Common shrew
PSGs

Fig. S4B

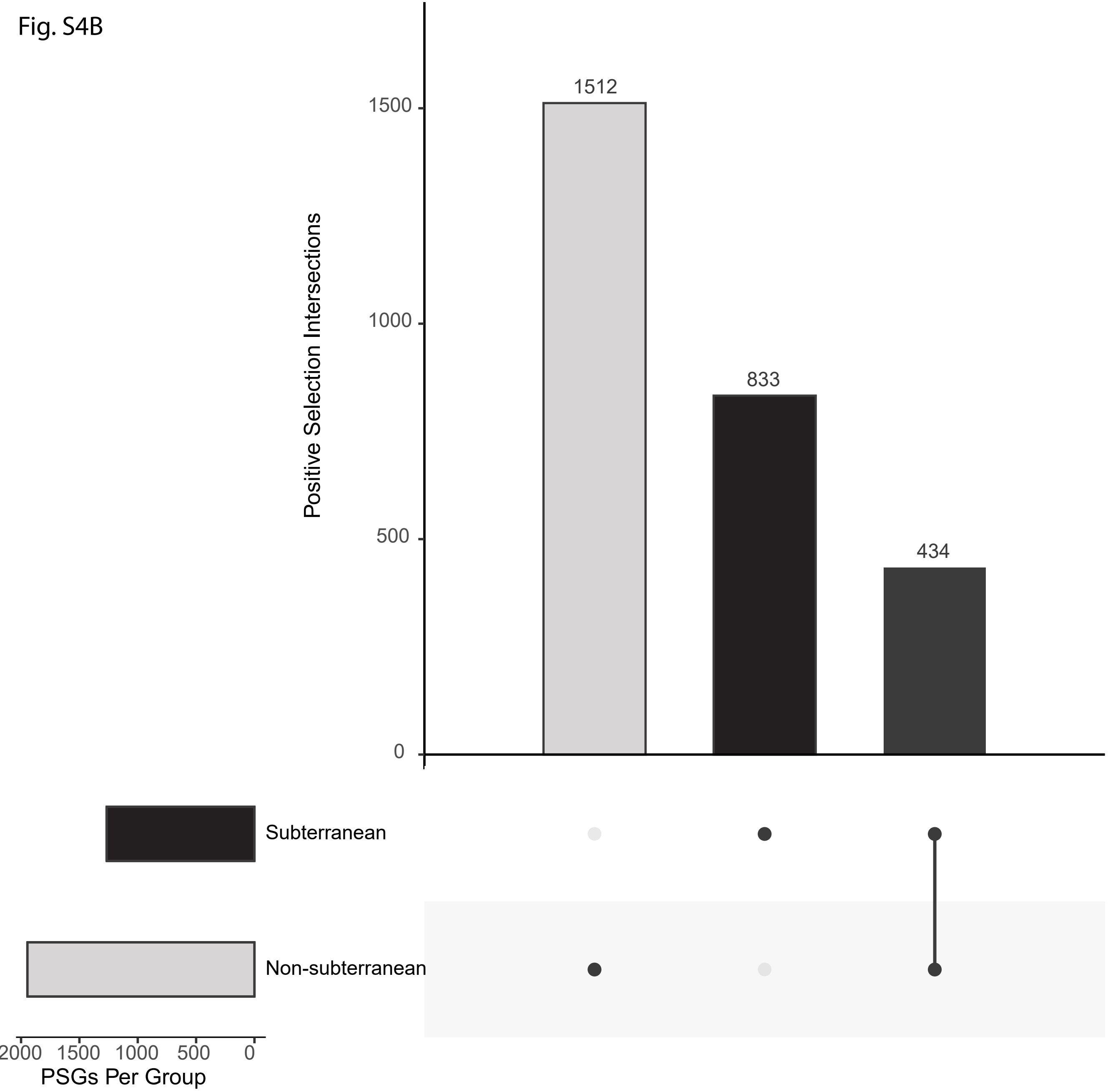

Positive Selection Intersections
1500
1000
500
0
1512
833
434
Subterranean
Non-subterranean
2000
1500
1000
500
0
PSGs Per Group

Fig. S4C

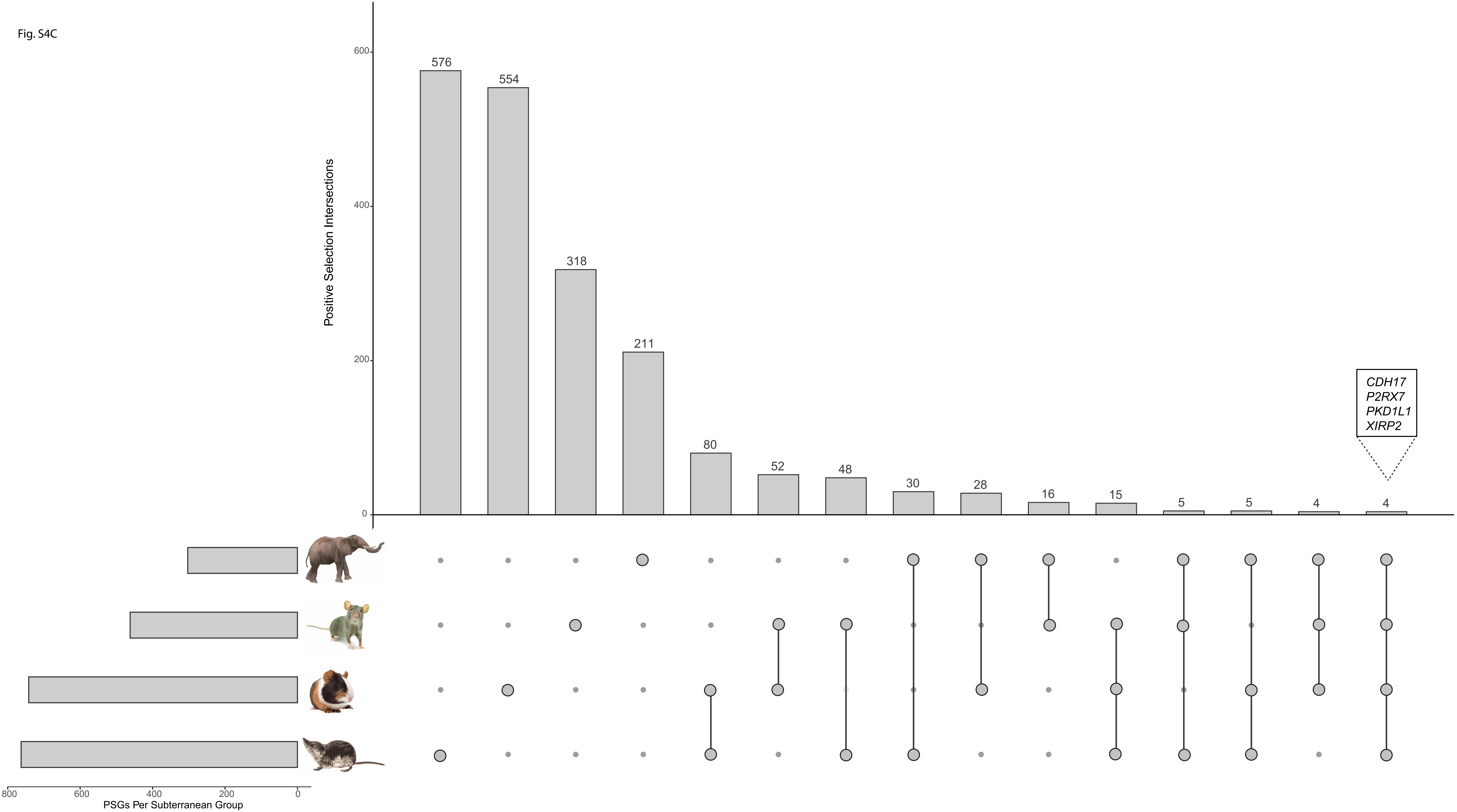

Positive Selection Intersections
600
400
200
0
576
554
318
211
80
52
48
30
28
16
15
5
5
4
4
CDH17
P2RX7
PKD1L1
XIRP2
800
600
400
200
0
PSGs Per Subterranean Group

Fig. S5A

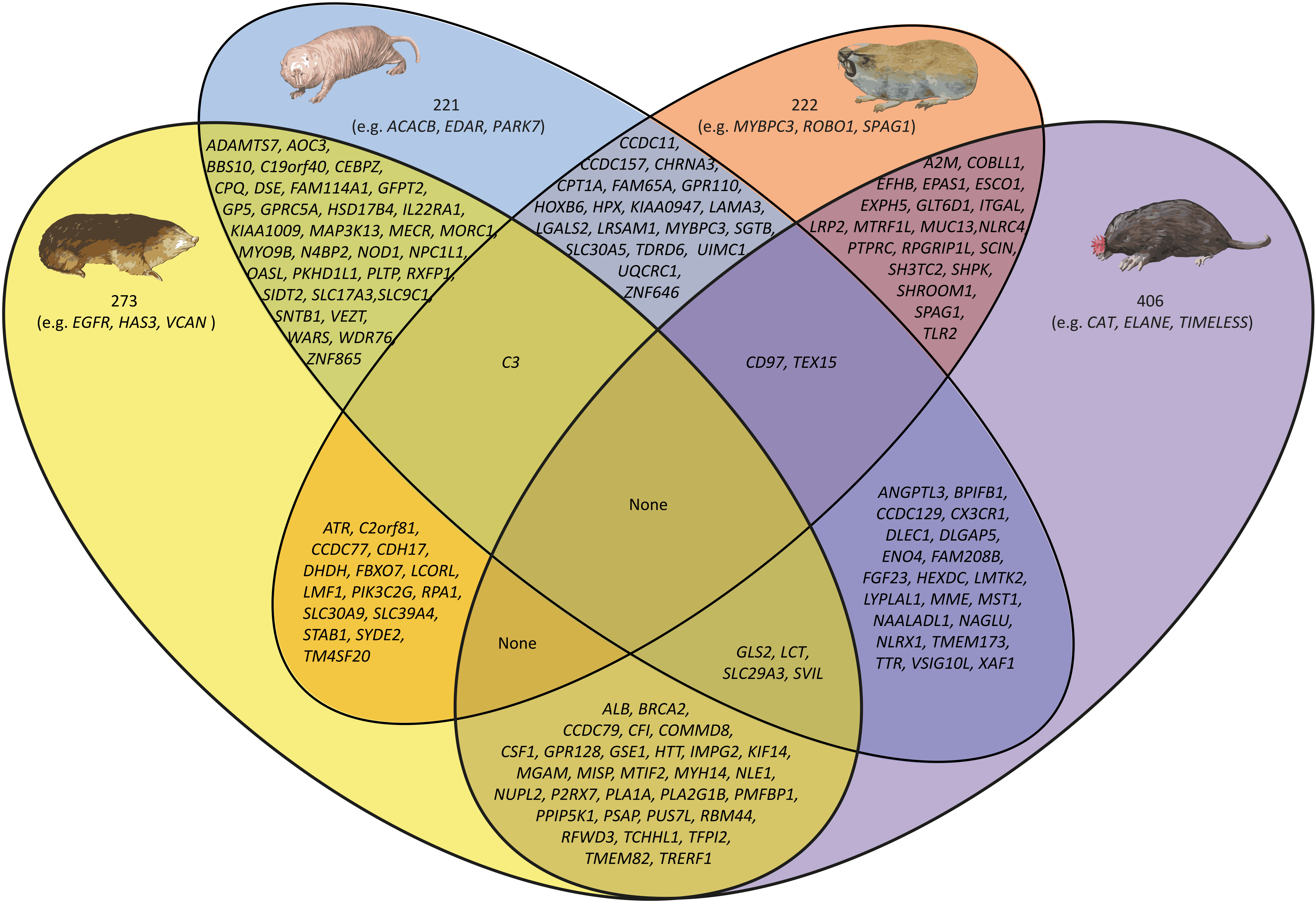
221
(e.g. ACACB, EDAR, PARK7)
222
(e.g. MYBPC3, ROBO1, SPAG1)
273
(e.g. EGFR, HAS3, VCAN)
406
(e.g. CAT, ELANE, TIMELESS)
ADAMTS7, AOC3, BBS10, C19orf40, CEBPZ, CPQ, DSE, FAM114A1, GFPT2, GP5, GPRC5A, HSD17B4, IL22RA1, KIAA1009, MAP3K13, MECR, MORC1, MYO9B, N4BP2, NOD1, NPC1L1, OASL, PKHD1L1, PLTP, RXFP1, SIDT2, SLC17A3,SLC9C1, SNTB1, VEZT, WARS, WDR76, ZNF865
CCDC11, CCDC157, CHRNA3, CPT1A, FAM65A, GPR110, HOXB6, HPX, KIAA0947, LAMA3, LGALS2, LRSAM1, MYBPC3, SGTB, SLC30A5, TDRD6, UIMC1, UQCRC1, ZNF646
A2M, COBLL1, EFHB, EPAS1, ESCO1, EXPH5, GLT6D1, ITGAL, LRP2, MTRF1L, MUC13,NLRC4, PTPRC, RPGRIP1L, SCIN, SH3TC2, SHPK, SHROOM1, SPAG1, TLR2
C3
CD97, TEX15
None
ANGPTL3, BPIFB1, CCDC129, CX3CR1, DLEC1, DLGAP5, ENO4, FAM208B, FGF23, HEXDC, LMTK2, LYPLAL1, MME, MST1, NAALADL1, NAGLU, NLRX1, TMEM173, TTR, VSIG10L, XAF1
ATR, C2orf81, CCDC77, CDH17, DHDH, FBXO7, LCORL, LMF1, PIK3C2G, RPA1, SLC30A9, SLC39A4, STAB1, SYDE2, TM4SF20
None
GLS2, LCT, SLC29A3, SVIL
ALB, BRCA2, CCDC79, CFI, COMMD8, CSF1, GPR128, GSE1, HTT, IMPG2, KIF14, MGAM, MISP, MTIF2, MYH14, NLE1, NUPL2, P2RX7, PLA1A, PLA2G1B, PMFBP1, PPIP5K1, PSAP, PUS7L, RBM44, RFWD3, TCHHL1, TFPI2, TMEM82, TRERF1

Fig. S5B

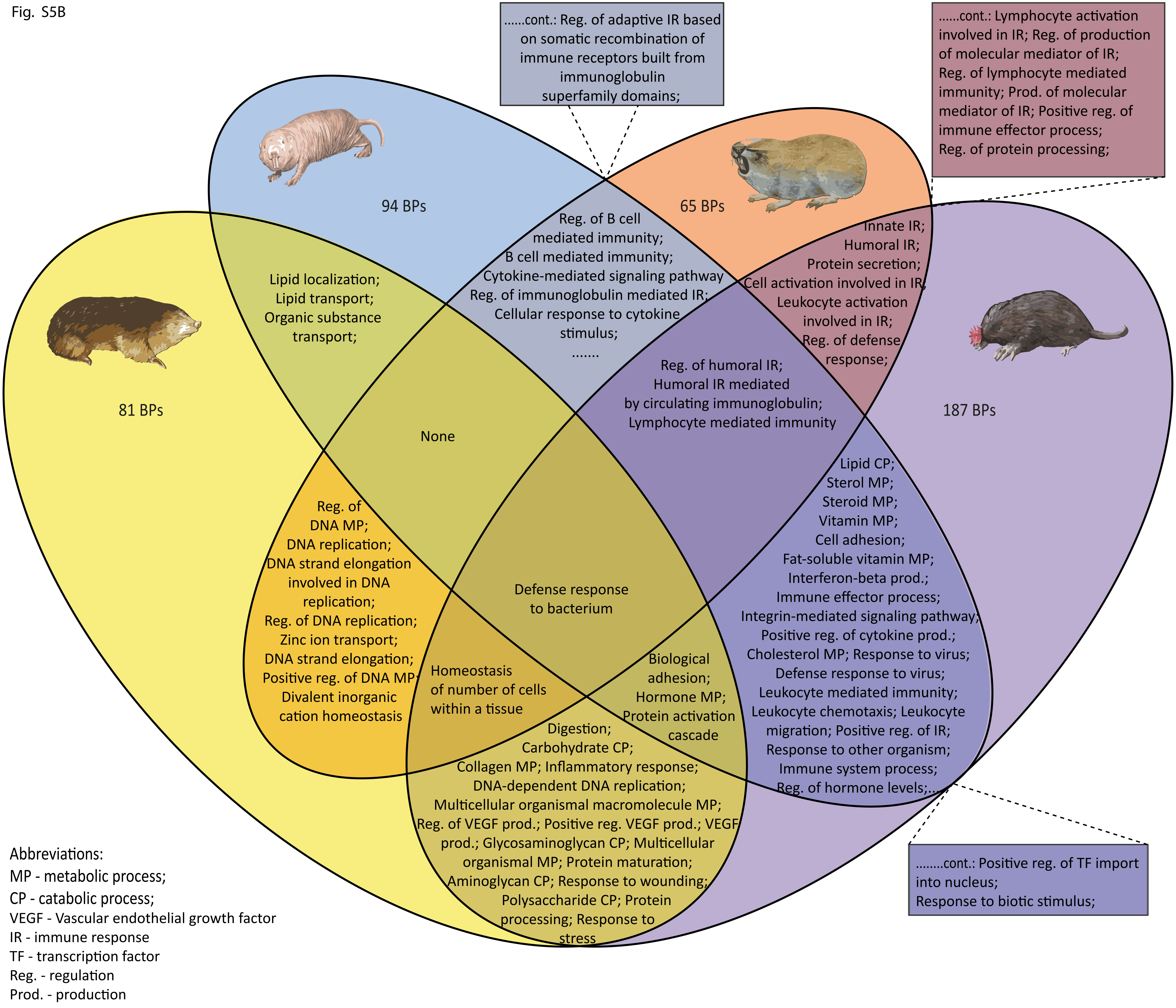

......cont.: Reg. of adaptive IR based on somatic recombination of immune receptors built from immunoglobulin superfamily domains;
......cont.: Lymphocyte activation involved in IR; Reg. of production of molecular mediator of IR; Reg. of lymphocyte mediated immunity; Prod. of molecular mediator of IR; Positive reg. of immune effector process; Reg. of protein processing;
94 BPs
65 BPs
81 BPs
187 BPs
Lipid localization; Lipid transport; Organic substance transport;
Reg. of B cell mediated immunity; B cell mediated immunity; Cytokine-mediated signaling pathway Reg. of immunoglobulin mediated IR; Cellular response to cytokine stimulus; ....
Innate IR; Humoral IR; Protein secretion; Cell activation involved in IR; Leukocyte activation involved in IR; Reg. of defense response;
None
Reg. of humoral IR; Humoral IR mediated by circulating immunoglobulin; Lymphocyte mediated immunity
Lipid CP; Sterol MP; Steroid MP; Vitamin MP; Cell adhesion; Fat-soluble vitamin MP; Interferon-beta prod.; Immune effector process; Integrin-mediated signaling pathway; Positive reg. of cytokine prod.; Cholesterol MP; Response to virus; Defense response to virus; Leukocyte mediated immunity; Leukocyte chemotaxis; Leukocyte migration; Positive reg. of IR; Response to other organism; Immune system process; Reg. of hormone levels;....
Reg. of DNA MP; DNA replication; DNA strand elongation involved in DNA replication; Reg. of DNA replication; Zinc ion transport; DNA strand elongation; Positive reg. of DNA MP; Divalent inorganic cation homeostasis
Defense response to bacterium
Homeostasis of number of cells within a tissue
Biological adhesion; Hormone MP; Protein activation cascade
Digestion; Carbohydrate CP; Collagen MP; Inflammatory response; DNA-dependent DNA replication; Multicellular organismal macromolecule MP; Reg. of VEGF prod.; Positive reg. VEGF prod.; VEGF prod.; Glycosaminoglycan CP; Multicellular organismal MP; Protein maturation; Aminoglycan CP; Response to wounding; Polysaccharide CP; Protein processing; Response to stress
........cont.: Positive reg. of TF import into nucleus; Response to biotic stimulus;
Abbreviations:
MP - metabolic process;
CP - catabolic process;
VEGF - Vascular endothelial growth factor
IR - immune response
TF - transcription factor
Reg. - regulation
Prod. - production

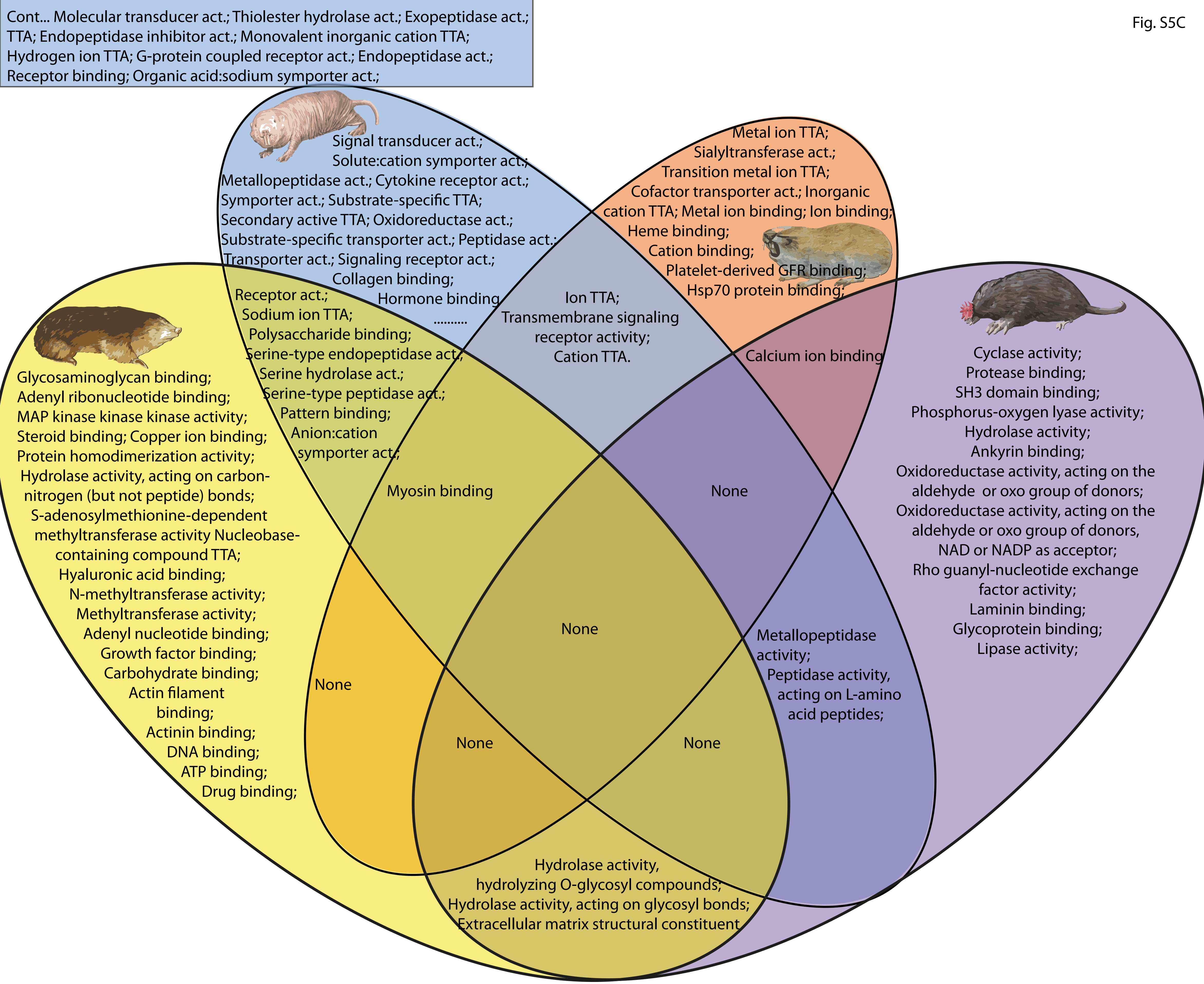

Fig. S5C
Cont... Molecular transducer act.; Thiolester hydrolase act.; Exopeptidase act.; TTA; Endopeptidase inhibitor act.; Monovalent inorganic cation TTA; Hydrogen ion TTA; G-protein coupled receptor act.; Endopeptidase act.; Receptor binding; Organic acid:sodium symporter act.;
Signal transducer act.; Solute:cation symporter act.; Metallopeptidase act.; Cytokine receptor act.; Symporter act.; Substrate-specific TTA; Secondary active TTA; Oxidoreductase act.; Substrate-specific transporter act.; Peptidase act.; Transporter act.; Signaling receptor act.; Collagen binding; Hormone binding ....
Metal ion TTA; Sialyltransferase act.; Transition metal ion TTA; Cofactor transporter act.; Inorganic cation TTA; Metal ion binding; Ion binding; Heme binding; Cation binding; Platelet-derived GFR binding; Hsp70 protein binding;
Ion TTA; Transmembrane signaling receptor activity; Cation TTA.
Calcium ion binding
Receptor act.; Sodium ion TTA; Polysaccharide binding; Serine-type endopeptidase act.; Serine hydrolase act.; Serine-type peptidase act.; Pattern binding; Anion:cation symporter act.;
Glycosaminoglycan binding; Adenyl ribonucleotide binding; MAP kinase kinase kinase activity; Steroid binding; Copper ion binding; Protein homodimerization activity; Hydrolase activity, acting on carbon-nitrogen (but not peptide) bonds; S-adenosylmethionine-dependent methyltransferase activity Nucleobase-containing compound TTA; Hyaluronic acid binding; N-methyltransferase activity; Methyltransferase activity; Adenyl nucleotide binding; Growth factor binding; Carbohydrate binding; Actin filament binding; Actinin binding; DNA binding; ATP binding; Drug binding;
Cyclase activity; Protease binding; SH3 domain binding; Phosphorus-oxygen lyase activity; Hydrolase activity; Ankyrin binding; Oxidoreductase activity, acting on the aldehyde or oxo group of donors; Oxidoreductase activity, acting on the aldehyde or oxo group of donors, NAD or NADP as acceptor; Rho guanyl-nucleotide exchange factor activity; Laminin binding; Glycoprotein binding; Lipase activity;
Myosin binding
None
None
None
None
None
Metallopeptidase activity; Peptidase activity, acting on L-amino acid peptides;
Hydrolase activity, hydrolyzing O-glycosyl compounds; Hydrolase activity, acting on glycosyl bonds; Extracellular matrix structural constituent

Fig.S5D

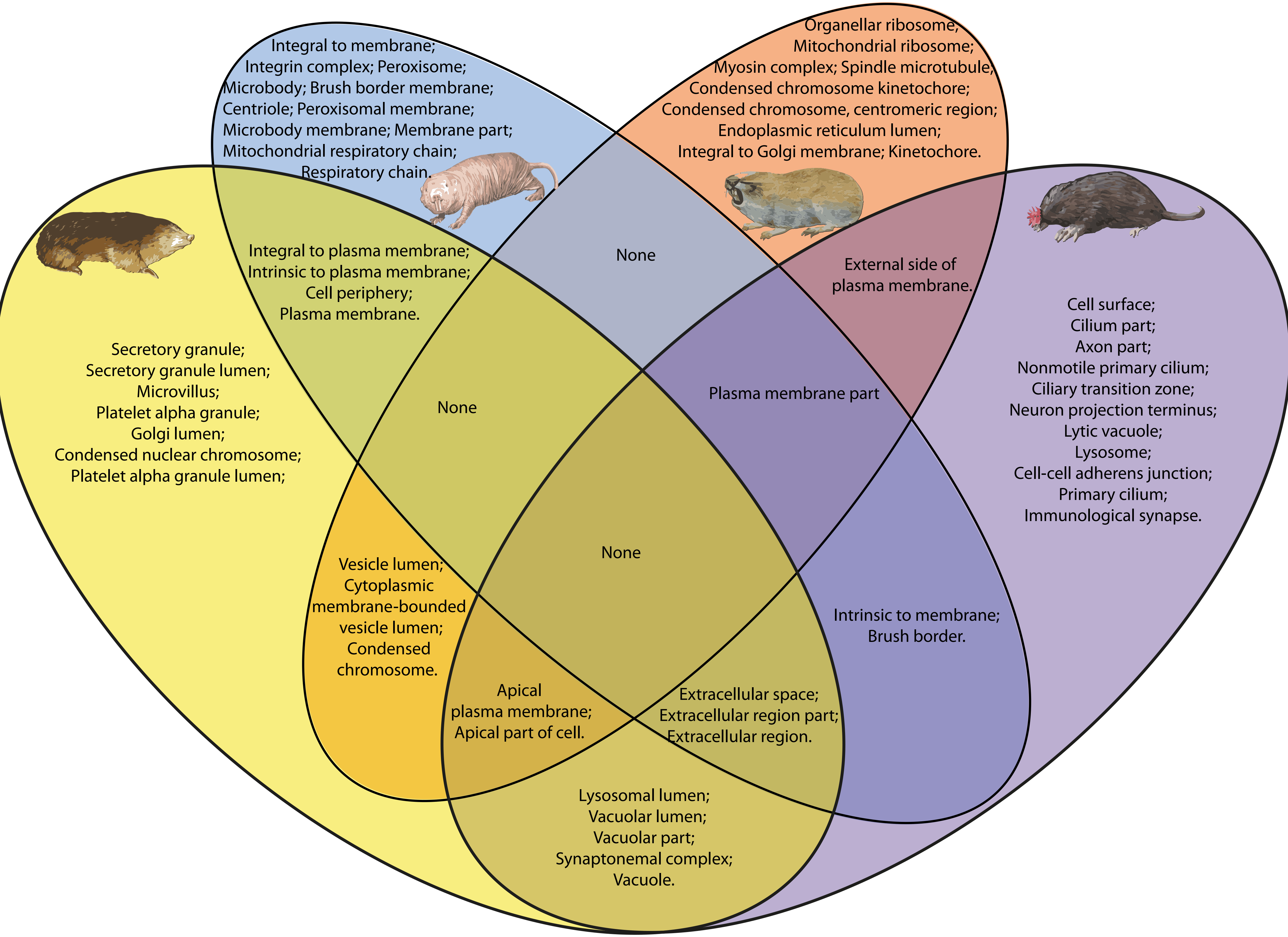

Integral to membrane;
Integrin complex; Peroxisome;
Microbody; Brush border membrane;
Centriole; Peroxisomal membrane;
Microbody membrane; Membrane part;
Mitochondrial respiratory chain;
Respiratory chain.
Organellar ribosome;
Mitochondrial ribosome;
Myosin complex; Spindle microtubule;
Condensed chromosome kinetochore;
Condensed chromosome, centromeric region;
Endoplasmic reticulum lumen;
Integral to Golgi membrane; Kinetochore.
None
Integral to plasma membrane;
Intrinsic to plasma membrane;
Cell periphery;
Plasma membrane.
External side of
plasma membrane.
Cell surface;
Cilium part;
Axon part;
Nonmotile primary cilium;
Ciliary transition zone;
Neuron projection terminus;
Lytic vacuole;
Lysosome;
Cell-cell adherens junction;
Primary cilium;
Immunological synapse.
Secretory granule;
Secretory granule lumen;
Microvillus;
Platelet alpha granule;
Golgi lumen;
Condensed nuclear chromosome;
Platelet alpha granule lumen;
None
Plasma membrane part
None
Vesicle lumen;
Cytoplasmic
membrane-bounded
vesicle lumen;
Condensed
chromosome.
Intrinsic to membrane;
Brush border.
Apical
plasma membrane;
Apical part of cell.
Extracellular space;
Extracellular region part;
Extracellular region.
Lysosomal lumen;
Vacuolar lumen;
Vacuolar part;
Synaptonemal complex;
Vacuole.

Fig. S6

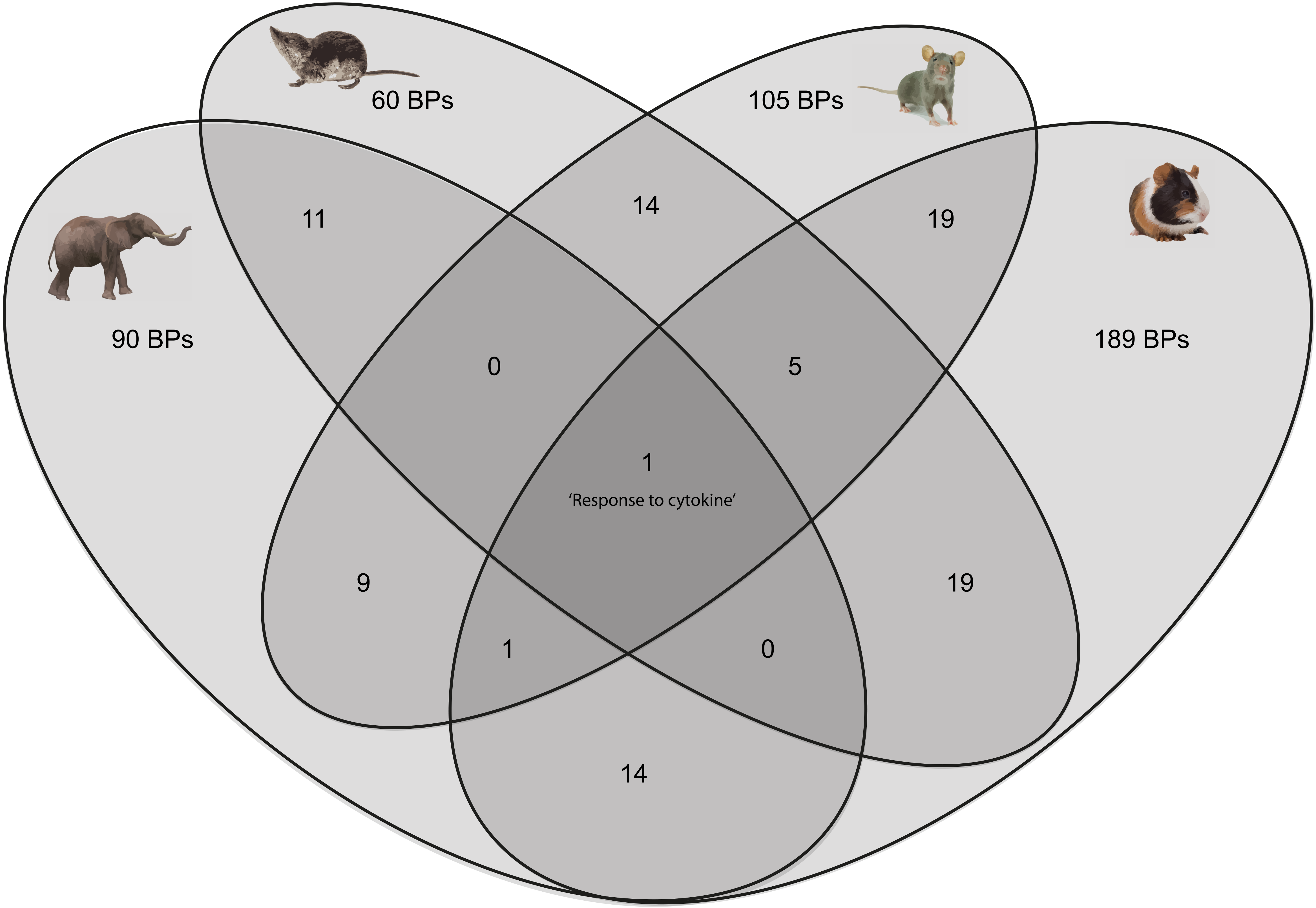

60 BPs
105 BPs
90 BPs
189 BPs
11
14
19
0
5
1
'Response to cytokine'
9
19
1
0
14

Fig. S7A

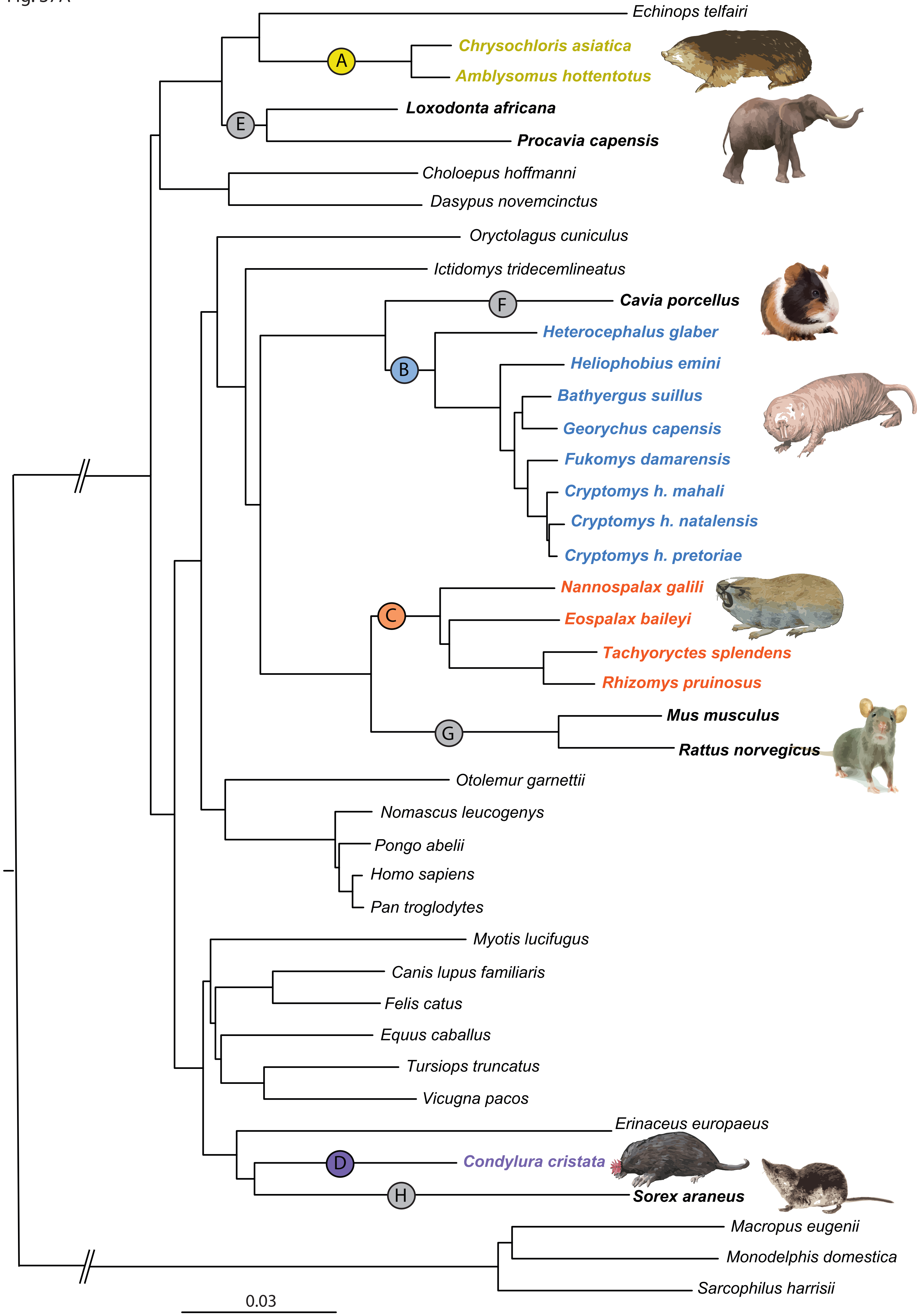

Echinops telfairi
A
Chrysochloris asiatica
Amblysomus hottentotus
E
Loxodonta africana
Procavia capensis
Choloepus hoffmanni
Dasypus novemcinctus
Oryctolagus cuniculus
Ictidomys tridecemlineatus
F
Cavia porcellus
Heterocephalus glaber
B
Heliophobius emini
Bathyergus suillus
Georychus capensis
Fukomys damarensis
Cryptomys h. mahali
Cryptomys h. natalensis
Cryptomys h. pretoriae
C
Nannospalax galili
Eospalax baileyi
Tachyoryctes splendens
Rhizomys pruinosus
G
Mus musculus
Rattus norvegicus
Otolemur garnettii
Nomascus leucogenys
Pongo abelii
Homo sapiens
Pan troglodytes
Myotis lucifugus
Canis lupus familiaris
Felis catus
Equus caballus
Tursiops truncatus
Vicugna pacos
Erinaceus europaeus
D
Condylura cristata
H
Sorex araneus
Macropus eugenii
Monodelphis domestica
Sarcophilus harrisii
0.03

Fig. S7B

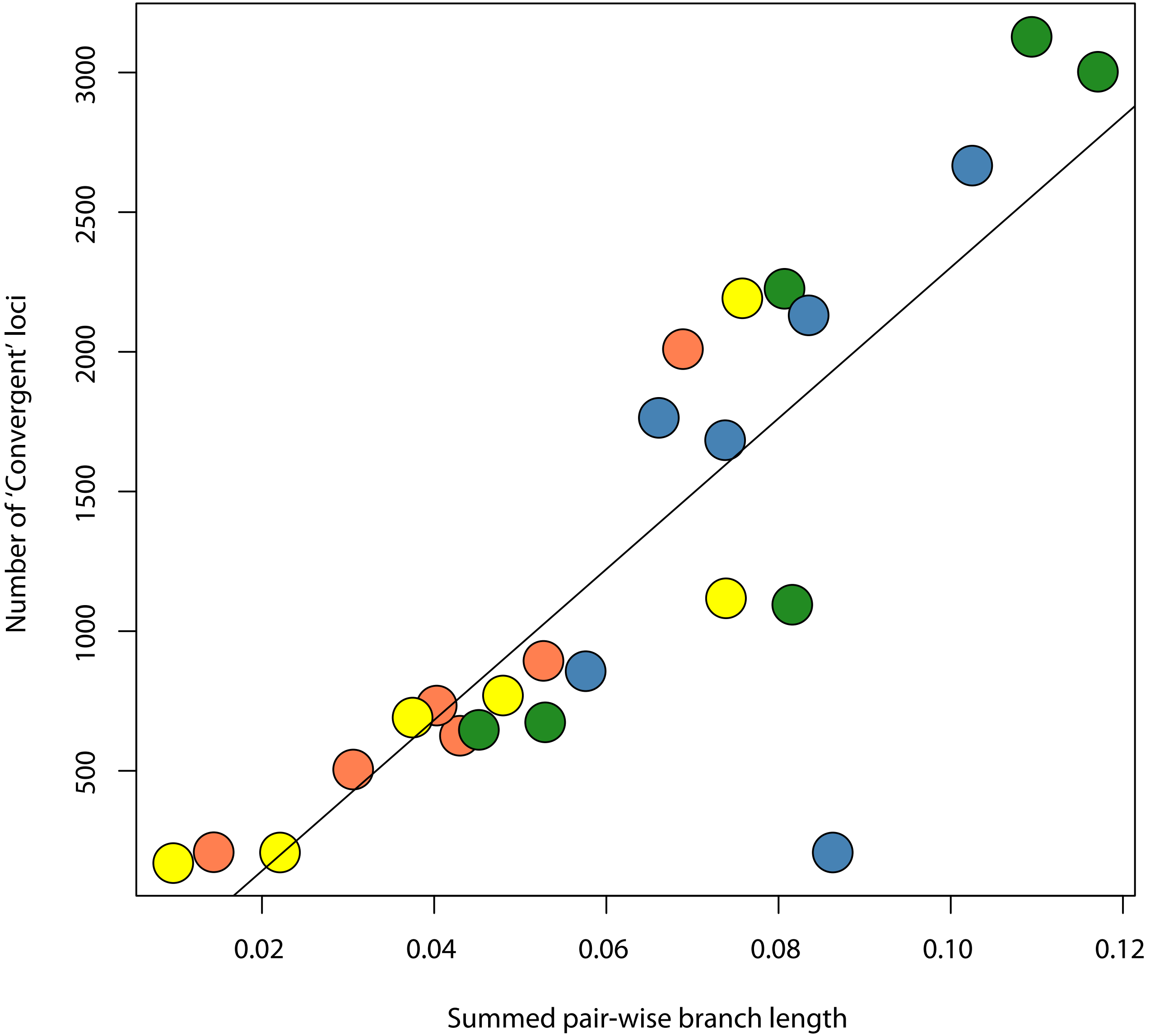

0.02
0.04
0.06
0.08
0.10
0.12
Summed pair-wise branch length
500
1000
1500
2000
2500
3000
Number of ‘Convergent’ loci

Fig. S8A

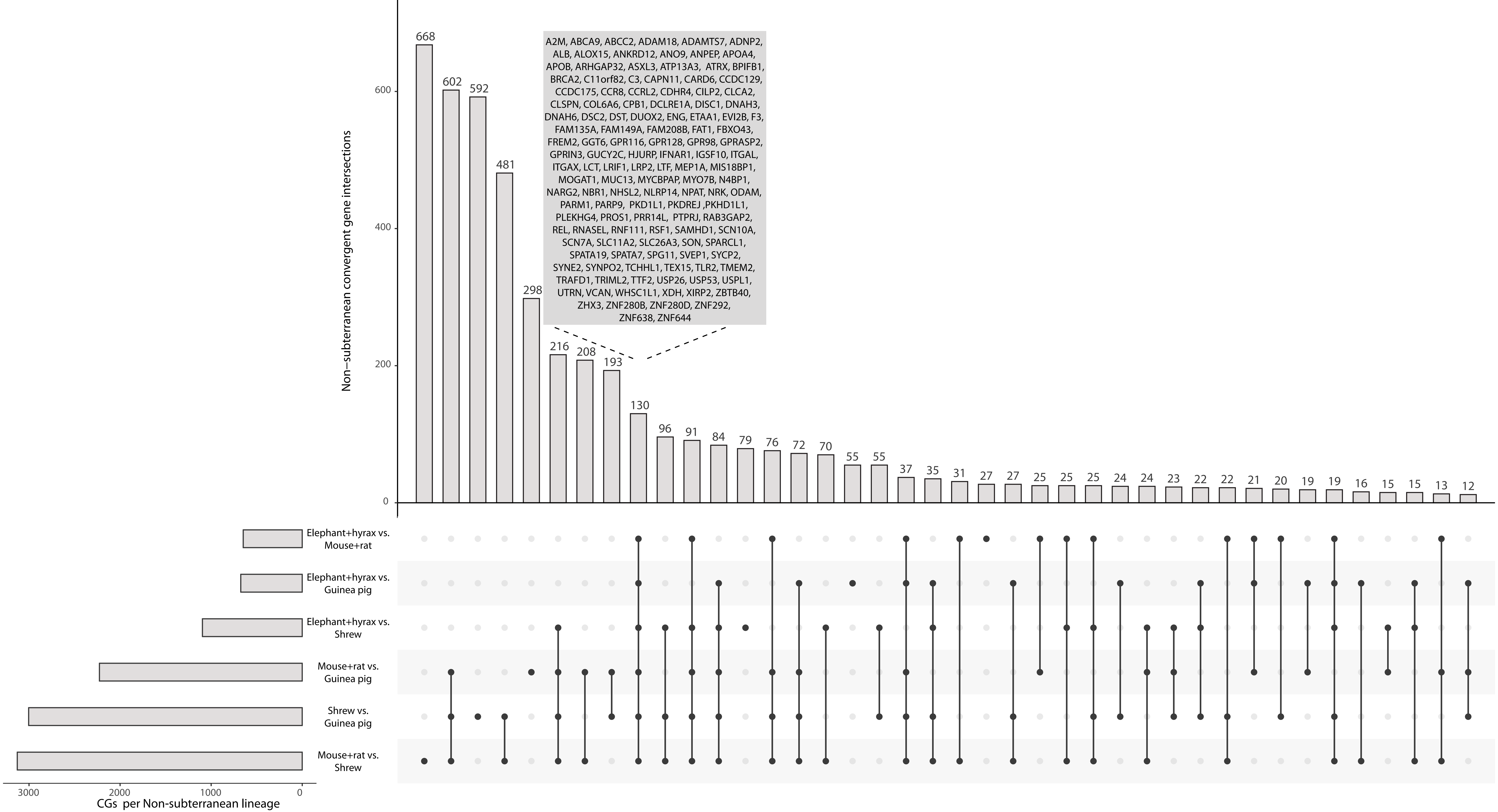

Non–subterranean convergent gene intersections
0
200
400
600
668
602
592
481
298
216
208
193
130
96
91
84
79
76
72
70
55
55
37
35
31
27
27
25
25
25
24
24
23
22
22
21
20
19
19
16
15
15
13
12
A2M, ABCA9, ABCC2, ADAM18, ADAMTS7, ADNP2,
ALB, ALOX15, ANKRD12, ANO9, ANPEP, APOA4,
APOB, ARHGAP32, ASXL3, ATP13A3, ATRX, BPIFB1,
BRCA2, C11orf82, C3, CAPN11, CARD6, CCDC129,
CCDC175, CCR8, CCRL2, CDHR4, CILP2, CLCA2,
CLSPN, COL6A6, CPB1, DCLRE1A, DISC1, DNAH3,
DNAH6, DSC2, DST, DUOX2, ENG, ETAA1, EVI2B, F3,
FAM135A, FAM149A, FAM208B, FAT1, FBXO43,
FREM2, GGT6, GPR116, GPR128, GPR98, GPRASP2,
GPRIN3, GUCY2C, HJURP, IFNAR1, IGSF10, ITGAL,
ITGAX, LCT, LRIF1, LRP2, LTF, MEP1A, MIS18BP1,
MOGAT1, MUC13, MYCBPAP, MYO7B, N4BP1,
NARG2, NBR1, NHSL2, NLRP14, NPAT, NRK, ODAM,
PARM1, PARP9, PKD1L1, PKDREJ ,PKHD1L1,
PLEKHG4, PROS1, PRR14L, PTPRJ, RAB3GAP2,
REL, RNASEL, RNF111, RSF1, SAMHD1, SCN10A,
SCN7A, SLC11A2, SLC26A3, SON, SPARCL1,
SPATA19, SPATA7, SPG11, SVEP1, SYCP2,
SYNE2, SYNPO2, TCHHL1, TEX15, TLR2, TMEM2,
TRAFD1, TRIML2, TTF2, USP26, USP53, USPL1,
UTRN, VCAN, WHSC1L1, XDH, XIRP2, ZBTB40,
ZHX3, ZNF280B, ZNF280D, ZNF292,
ZNF638, ZNF644
Elephant+hyrax vs. Mouse+rat
Elephant+hyrax vs. Guinea pig
Elephant+hyrax vs. Shrew
Mouse+rat vs. Guinea pig
Shrew vs. Guinea pig
Mouse+rat vs. Shrew
3000
2000
1000
0
CGs per Non-subterranean lineage

Fig. S8B

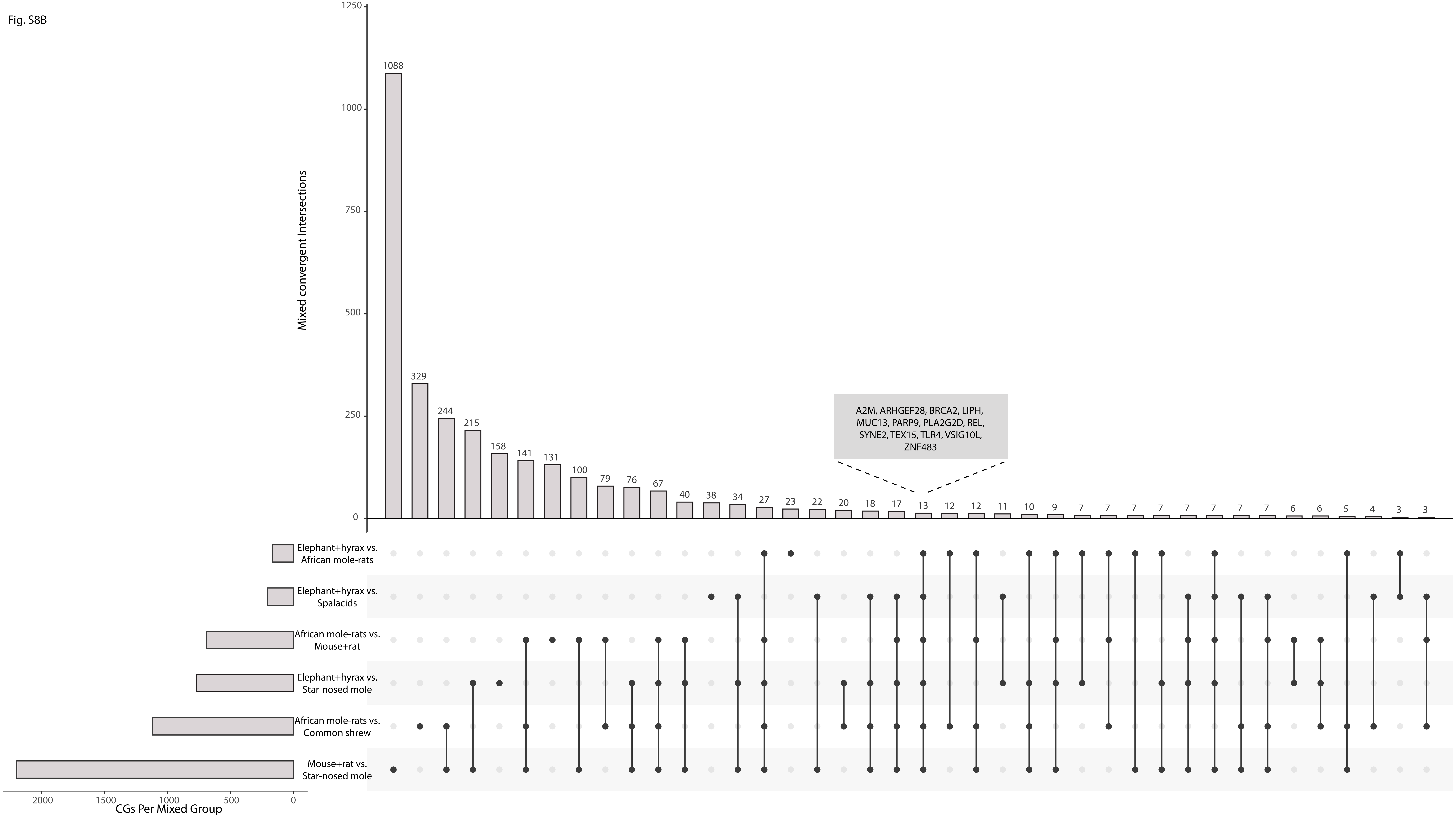

Mixed convergent Intersections
1250
1000
750
500
250
0
1088
329
244
215
158
141
131
100
79
76
67
40
38
34
27
23
22
20
18
17
13
12
12
11
10
9
7
7
7
7
7
7
7
7
6
6
5
4
3
3
A2M, ARHGEF28, BRCA2, LIPH, MUC13, PARP9, PLA2G2D, REL, SYNE2, TEX15, TLR4, VSIG10L, ZNF483
Elephant+hyrax vs. African mole-rats
Elephant+hyrax vs. Spalacids
African mole-rats vs. Mouse+rat
Elephant+hyrax vs. Star-nosed mole
African mole-rats vs. Common shrew
Mouse+rat vs. Star-nosed mole
2000
1500
1000
500
0
CGs Per Mixed Group

Fig. S8C

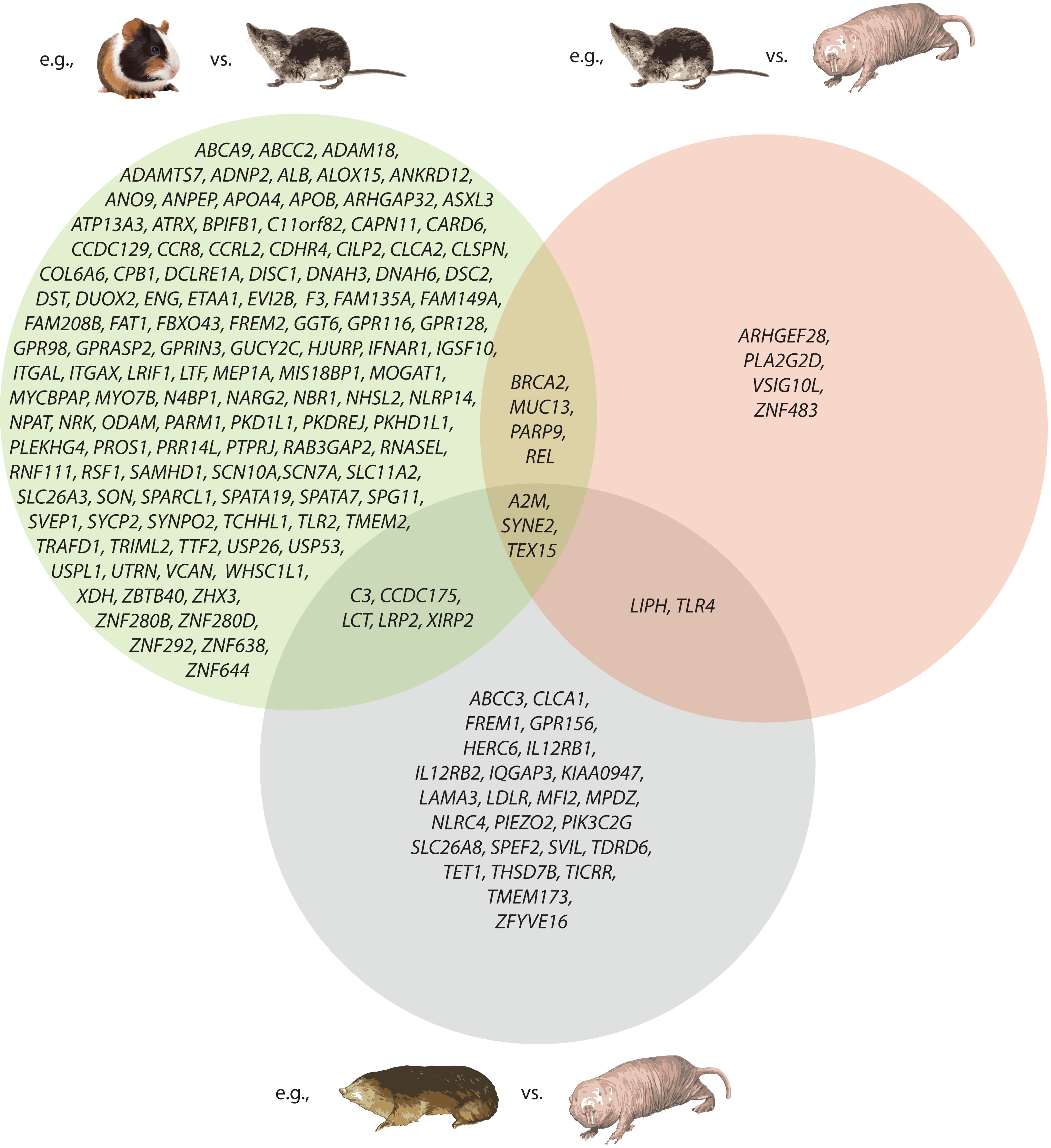
e.g.,
vs.
e.g.,
vs.
ABCA9, ABCC2, ADAM18, ADAMTS7, ADNP2, ALB, ALOX15, ANKRD12, ANO9, ANPEP, APOA4, APOB, ARHGAP32, ASXL3 ATP13A3, ATRX, BPIFB1, C11orf82, CAPN11, CARD6, CCDC129, CCR8, CCRL2, CDHR4, CILP2, CLCA2, CLSPN, COL6A6, CPB1, DCLRE1A, DISC1, DNAH3, DNAH6, DSC2, DST, DUOX2, ENG, ETAA1, EVI2B, F3, FAM135A, FAM149A, FAM208B, FAT1, FBXO43, FREM2, GGT6, GPR116, GPR128, GPR98, GPRASP2, GPRIN3, GUCY2C, HJURP, IFNAR1, IGSF10, ITGAL, ITGAX, LRIF1, LTF, MEP1A, MIS18BP1, MOGAT1, MYCBPAP, MYO7B, N4BP1, NARG2, NBR1, NHSL2, NLRP14, NPAT, NRK, ODAM, PARM1, PKD1L1, PKDREJ, PKHD1L1, PLEKHG4, PROS1, PRR14L, PTPRJ, RAB3GAP2, RNASEL, RNF111, RSF1, SAMHD1, SCN10A,SCN7A, SLC11A2, SLC26A3, SON, SPARCL1, SPATA19, SPATA7, SPG11, SVEP1, SYCP2, SYNPO2, TCHHL1, TLR2, TMEM2, TRAFD1, TRIML2, TTF2, USP26, USP53, USPL1, UTRN, VCAN, WHSC1L1, XDH, ZBTB40, ZHX3, ZNF280B, ZNF280D, ZNF292, ZNF638, ZNF644
ARHGEF28, PLA2G2D, VSIG10L, ZNF483
BRCA2, MUC13, PARP9, REL
A2M, SYNE2, TEX15
C3, CCDC175, LCT, LRP2, XIRP2
LIPH, TLR4
ABCC3, CLCA1, FREM1, GPR156, HERC6, IL12RB1, IL12RB2, IQGAP3, KIAA0947, LAMA3, LDLR, MFI2, MPDZ, NLRC4, PIEZO2, PIK3C2G SLC26A8, SPEF2, SVIL, TDRD6, TET1, THSD7B, TICRR, TMEM173, ZFYVE16
e.g.,
vs.

Fig. S9

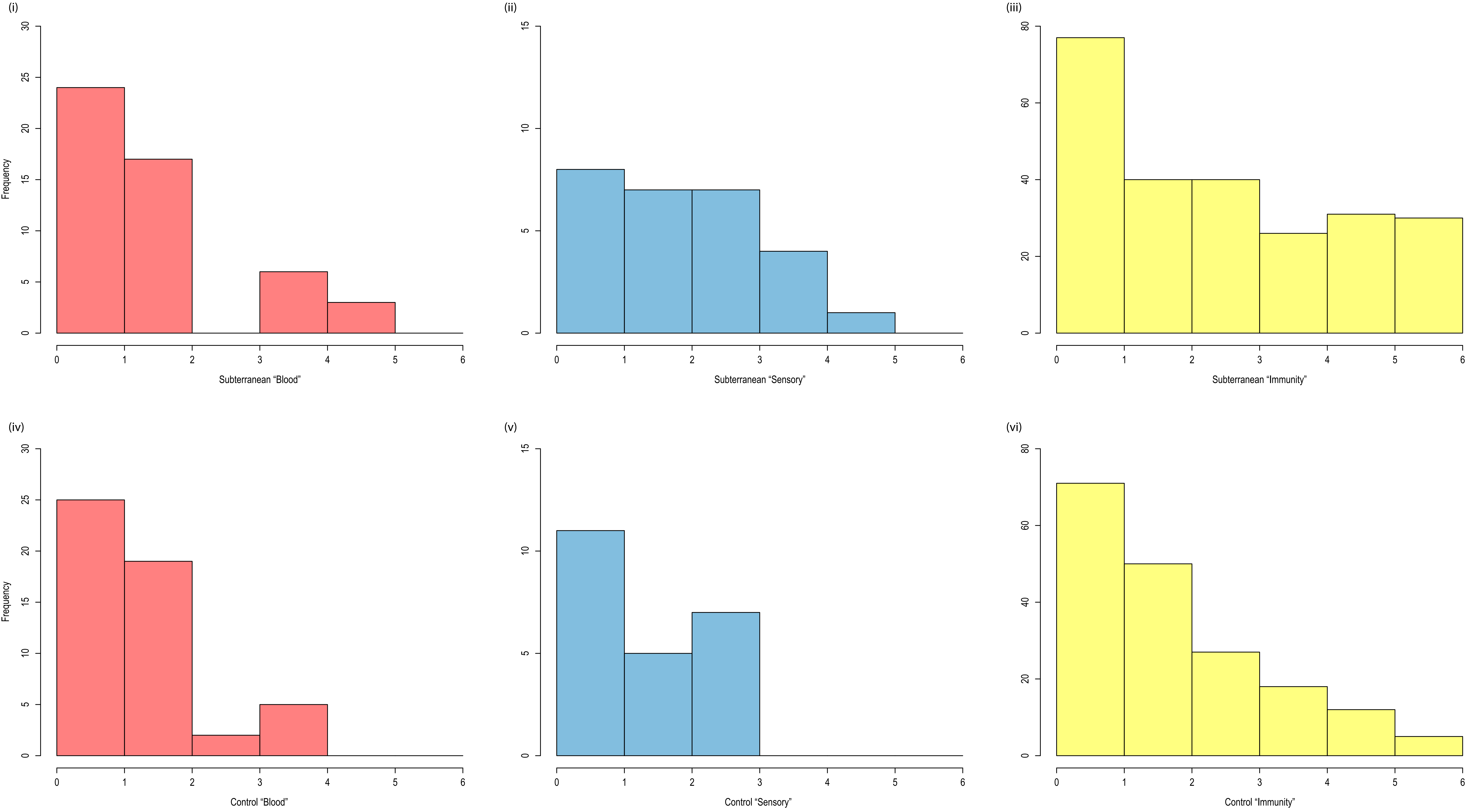
(i)
(ii)
(iii)
(iv)
(v)
(vi)
Frequency
Frequency
Subterranean “Blood”
Subterranean “Sensory”
Subterranean “Immunity”
Control “Blood”
Control “Sensory”
Control “Immunity”

Fig. S10A

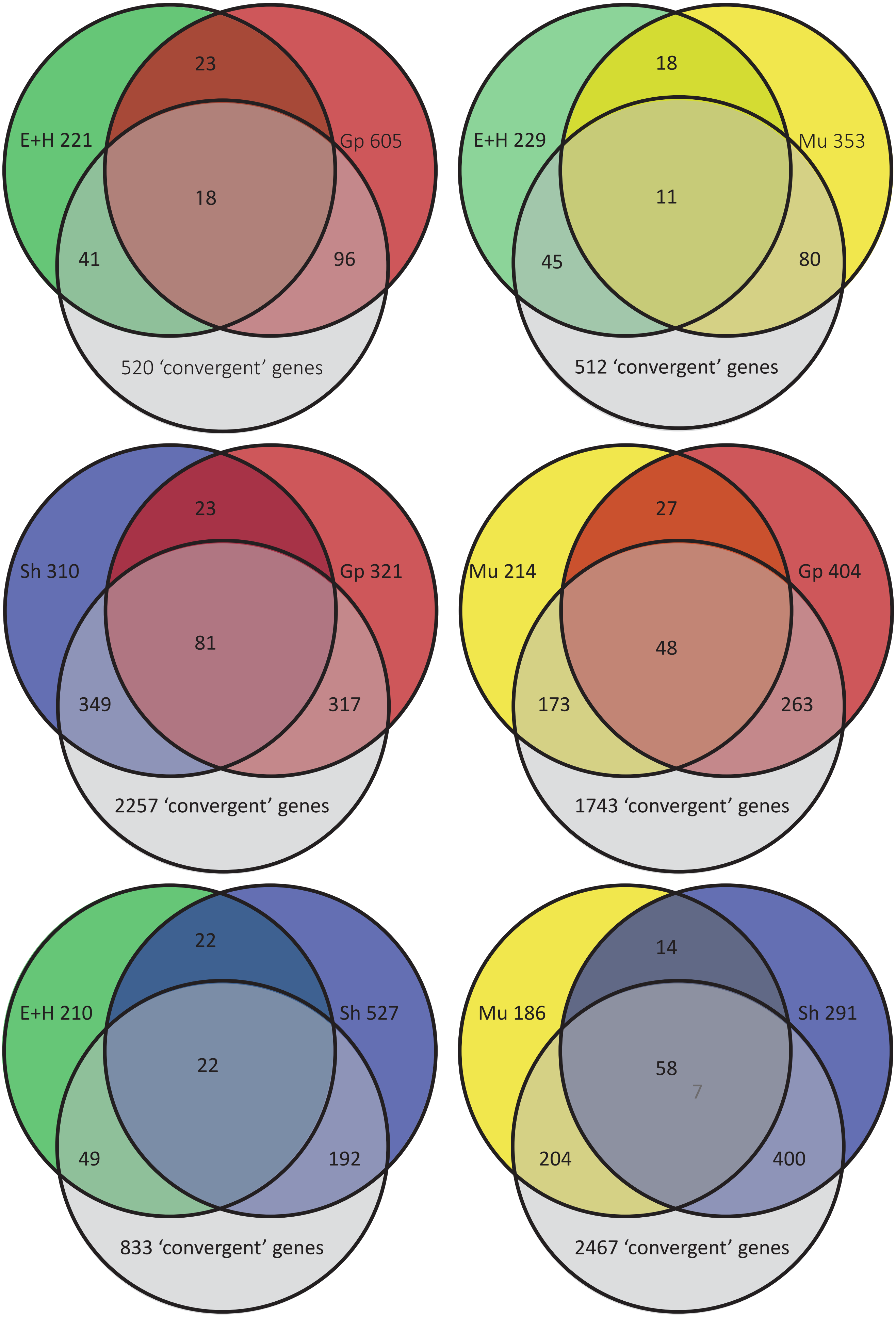
23
E+H 221
Gp 605
18
41
96
520 'convergent' genes
18
E+H 229
Mu 353
11
45
80
512 'convergent' genes
23
Sh 310
Gp 321
81
349
317
2257 'convergent' genes
27
Mu 214
Gp 404
48
173
263
1743 'convergent' genes
22
E+H 210
Sh 527
22
49
192
833 'convergent' genes
14
Mu 186
Sh 291
58
7
204
400
2467 'convergent' genes

Fig. S10B

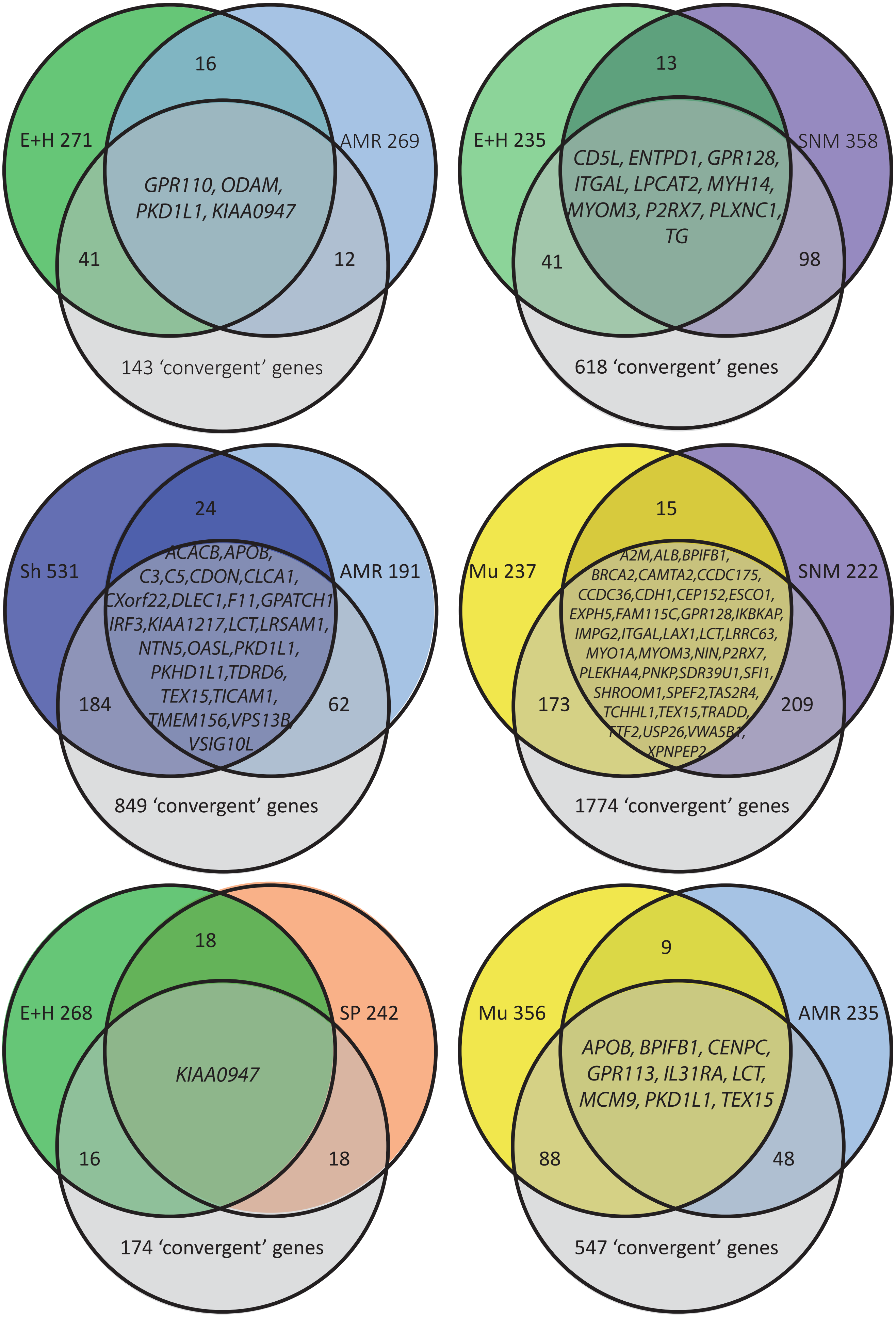

16
E+H 271
AMR 269
GPR110, ODAM,
PKD1L1, KIAA0947
41
12
143 ‘convergent’ genes
13
E+H 235
SNM 358
CD5L, ENTPD1, GPR128,
ITGAL, LPCAT2, MYH14,
MYOM3, P2RX7, PLXNC1,
TG
41
98
618 ‘convergent’ genes
24
Sh 531
AMR 191
ACACB,APOB,
C3,C5,CDON,CLCA1,
CXorf22,DLEC1,F11,GPATCH1
IRF3,KIAA1217,LCT,LRSAM1,
NTN5,OASL,PKD1L1,
PKHD1L1,TDRD6,
TEX15,TICAM1,
TMEM156,VPS13B,
VSIG10L
184
62
849 ‘convergent’ genes
15
Mu 237
SNM 222
A2M,ALB,BPIFB1,
BRCA2,CAMTA2,CCDC175,
CCDC36,CDH1,CEP152,ESCO1,
EXPH5,FAM115C,GPR128,IKBKAP,
IMPG2,ITGAL,LAX1,LCT,LRRC63,
MYO1A,MYOM3,NIN,P2RX7,
PLEKHA4,PNKP,SDR39U1,SFI1,
SHROOM1,SPEF2,TAS2R4,
TCHHL1,TEX15,TRADD,
TTF2,USP26,VWA5B1,
XPNPEP2
173
209
1774 ‘convergent’ genes
18
E+H 268
SP 242
KIAA0947
16
18
174 ‘convergent’ genes
9
Mu 356
AMR 235
APOB, BPIFB1, CENPC,
GPR113, IL31RA, LCT,
MCM9, PKD1L1, TEX15
88
48
547 ‘convergent’ genes
